# mHealth intervention “ImTeCHO” to improve delivery of maternal, neonatal, and child care services—A cluster-randomized trial in tribal areas of Gujarat, India

**DOI:** 10.1371/journal.pmed.1002939

**Published:** 2019-10-24

**Authors:** Dhiren Modi, Nishith Dholakia, Ravi Gopalan, Sethuraman Venkatraman, Kapilkumar Dave, Shobha Shah, Gayatri Desai, Shamim A. Qazi, Anju Sinha, Ravindra Mohan Pandey, Ankit Anand, Shrey Desai, Pankaj Shah

**Affiliations:** 1 Community Health Department, SEWA Rural, Jhagadia, Bharuch, Gujarat, India; 2 Commissionerate of Health, Government of Gujarat, Gandhinagar, Gujarat, India; 3 Argusoft India Ltd., Gandhinagar, Gujarat, India; 4 Department of Maternal, Newborn, Child and Adolescent Health, World Health Organization, Geneva, Switzerland; 5 Division of Reproductive Biology, Maternal & Child Health, Indian Council of Medical Research, New Delhi, India; 6 Department of Biostatistics, All India Institute of Medical Sciences, New Delhi, India; 7 Population Research Centre, Institute of Social and Economic Change, Bengaluru, India; University of Manchester, UNITED KINGDOM

## Abstract

**Background:**

The coverage of community-based maternal, neonatal, and child health (MNCH) services remains low, especially in hard-to-reach areas. We evaluated the effectiveness of a mobile-phone–and web-based application, Innovative Mobile-phone Technology for Community Health Operations (ImTeCHO), as a job aid to the government’s Accredited Social Health Activists (ASHAs) and Primary Health Center (PHC) staff to improve coverage of MNCH services in rural tribal communities of Gujarat, India.

**Methods and findings:**

This open cluster-randomized trial was conducted in 22 PHCs in six tribal blocks of Bharuch and Narmada districts in India. The ImTeCHO mobile-phone–and web-based application included various technology-based job aids to facilitate scheduling of home visits, screening for complications, counseling during home visits, and supportive supervision by PHC staff. Primary outcome indicators were a composite index calculated based on coverage of important MNCH services and coverage of at least two home visitations by ASHA within the first week of birth. Primary analysis was intention to treat (ITT). Generalized Estimating Equation (GEE) was used to account for clustering. Eleven PHCs each were randomly allocated to the intervention (280 ASHAs, population: 234,134) and control (281 ASHAs, population: 242,809) arms. The intervention was implemented from February, 2016 to January, 2017. At the end of the implementation, 6,493 mothers were surveyed. Most of the surveyed women were tribal (5,571, 85.8%), and reported having a government-issued certificate for living below poverty line (4,916, 75.7%). The coverage of at least two home visits within first week of birth was 32.4% in the intervention clusters compared to 22.9% in the control clusters (adjusted effect size 10.2 [95% CI: 6.4, 14.0], *p* < 0.001). Mean number of home visits within first week of birth was 1.11 and 0.80 for intervention and control clusters, respectively (adjusted effect size 0.34 [95% CI: 0.23, 0.45], *p* < 0.001). The composite coverage index was 43.0% in the intervention clusters compared to 38.5% (adjusted effect size 4.9 [95% CI: 0.2, 9.5], *p* = 0.03) in the control clusters. There were substantial improvements in coverage home visits by ASHAs during antenatal period (adjusted effect size 15.7 [95% CI: 11.0, 20.4], *p* < 0.001), postnatal period (adjusted effect size 6.4, [95% CI: 3.2, 9.6], *p* <0.001), early initiation of breastfeeding (adjusted effect size 7.8 [95% CI: 4.2, 11.4], *p* < 0.001), and exclusive breastfeeding (adjusted effect size 13.4 [95% CI: 8.9, 17.9], *p* < 0.001). Number of infant and neonatal deaths was similar in the two arms in the ITT analysis. The limitations of the study include potential risk of inaccuracies in reporting events that occurred during pregnancy by the mothers and the duration of intervention being 12 months, which might be considered short.

**Conclusions:**

In this study, we found that use of ImTeCHO mobile- and web-based application as a job aid by government ASHAs and PHC staff improved coverage and quality of MNCH services in hard-to-reach areas. Supportive supervision, change management, and timely resolution of technology-related issues were critical implementation considerations to ensure adherence to the intervention.

**Trial registration:**

Study was registered at the Clinical Trial Registry of India (www.ctri.nic.in). Trial number: CTRI/2015/06/005847. The trial was registered (prospective) on 3 June, 2015. First enrollment was done on 26 August, 2015.

## Abstract 2

Shrey Desai and colleagues reveal the benefits of mobile phone and online information to help improve maternal, neonatal, and child health in hard-to-reach areas in India.

## Author summary

**Why was this study done?:**

- Delivery of community-based maternal, neonatal, and child health (MNCH) services can improve health outcomes and reduce preventable infant deaths. To improve the coverage of proven MNCH services, a new cadre of village-based frontline workers, called Accredited Social Health Activists (ASHAs), was introduced by the government of India in 2005. However, the coverage and quality of MNCH services delivered by ASHAs remains low, especially in hard-to-reach tribal areas.
- Mobile-phone-technology–based health (mHealth) solutions are promising, innovative strategies with the potential to improve performance of frontline health workers in controlled settings. However, there is a lack of robust evidence about the effectiveness of mHealth solutions implemented within the public health system as a job aid to frontline health workers towards improving coverage of MNCH services.

**What did researchers do and find?:**

- Twenty-two Primary Health Centers (PHCs) were randomly allocated to usual care from public health providers from the government with (11 PHCs, 280 ASHAs, population: 234,134) or without (11 PHCs, 281 ASHAs, population: 242,809) an mHealth intervention. The mHealth intervention included a package of various mobile-phone-technology–based job aids to facilitate longitudinal tracking, scheduling of health services, screening for complications, counseling for behavior change communication, and real-time monitoring and supportive supervision by supervisory staff at PHCs. Twelve months after the implementation, mothers were surveyed.
- We found that coverage and quality of most of the MNCH services were significantly higher among PHCs that were served by ASHAs who used mHealth as a job aid compared to those who did not. The coverage of at least two home visits within first week of birth was 32.4% in the mHealth intervention group, compared to 22.9% in the control group. A composite index, which was calculated based on coverage of multiple key MNCH services, was 43.0% in the mHealth intervention group compared to 38.5% in the nonintervention group.
- The ASHAs regularly used and adhered to the mHealth intervention; however, its use was low among the PHC staff.

**What do these findings mean?:**

- To our knowledge, this is the one of the first studies of its kind that assessed effectiveness of multiple mHealth job aids implemented in a low- and middle-income country (LMIC) through frontline health workers in an existing public health system throughout the continuum of care to improve wide range of MNCH outcomes among hard-to-reach populations using a robust research methodology. Adequate supportive supervision, change management, and ongoing technology assistance is crucial to ensure satisfactory adherence to the intervention.
- Our findings support the scale-up of mobile-phone-technology–based interventions as a job aid to frontline health workers to improve health outcomes.

## Introduction

Every year, there are 303,000 maternal deaths and 5.4 million child deaths worldwide [1,2]. Many of these deaths are preventable by increasing coverage and quality of proven, cost-effective maternal, neonatal, and child health (MNCH) services; quite a few are community-based services delivered by frontline health workers [3–5]. Mobile-phone-technology–based health (mHealth) solutions are promising, innovative strategies with the potential to increase effective coverage of some of the MNCH services through improving performance of frontline health workers in controlled settings on a limited scale [6–8]. However, there is a lack of good-quality evidence about the effectiveness of mHealth solutions implemented within the public health system towards improving the performance of frontline health workers throughout the continuum of care for MNCH, especially among hard-to-reach populations having poor coverage of services using robust implementation research methodologies [9–11].

India has made significant progress through its well-established primary healthcare system to deliver community-based services. A Primary Health Center (PHC) is a second-level health facility providing a range of curative and preventive primary healthcare services, covering populations of 20,000–25,000 in tribal areas, and is at the core of India’s primary healthcare system [12]. India, because of its large population, has a huge burden of malnutrition and maternal and child mortality [13–15]. The government of India introduced a cadre of village-based health workers, Accredited Social Health Activists (ASHAs), in 2005 to improve coverage of preventive MNCH services [16]. Some evidence-based community-based MNCH services delivered or facilitated by ASHAs through home visits and community mobilization activities include promotion to seek Antenatal care (ANC), iron and folic acid supplementation for pregnant women, promotion of facility-based delivery by skilled birth attendants, home visits for postnatal care of mothers and newborns, promotion of exclusive breastfeeding for first six months of life, immunization and complementary feeding, vitamin A supplementation, and identification of common childhood illnesses like fever, diarrhea, and pneumonia and their management [3,4,17].

In hard-to-reach and tribal areas, the performance of ASHAs and coverage of MNCH services have been reported as suboptimal, mainly because of inadequate training and insufficient supportive supervision and motivation [16,18–21]. To address these issues, we developed an mHealth application that consisted of a variety of technology-based job aids for the different levels of health workers involved in the delivery of health services. We called it "Innovative Mobile-phone Technology for Community Health Operations" (ImTeCHO) [22,23].

The objective of our trial was to implement and evaluate the effectiveness of ImTeCHO in tribal and rural areas of Gujarat to increase coverage of community-based MNCH services [23]. We hypothesized that ImTeCHO would lead to improved supervision, support, and motivation of ASHAs and PHC staff, resulting in improved coverage of proven MNCH services, including recommended number of home visits by ASHAs within first week of delivery and a composite outcome consisting of services across the continuum of care.

## Methods

The details about development of ImTeCHO and protocol of the study (S1 Text) has been already published earlier [22,23]. We provide some salient features below.

### Study design and setting

This open cluster-randomized implementation research trial was conducted within the existing public health system involving 22 PHCs serving a total population of 4,76,943 in predominantly tribal areas in the southern part of Gujarat, India. Eleven PHCs were randomized to the intervention and 11 to the control arm. Each PHC served a population of approximately 22,000. Cluster randomization was chosen because this was a complex intervention to be delivered at the community level. PHCs were selected as a cluster to reduce contamination because participants of the trial included ASHAs and staff of the PHC. Each PHC had a team of two medical doctors/officers, one Auxiliary Nurse Midwife (ANM), and one male multipurpose worker for every 4,000 to 6,000 people; one female and male health supervisor and one ASHA for approximately 1,000 people; and administrative staff. An ASHA is a female village-based frontline health worker who is a native of the village, has at least eight years of formal education, spends 3–5 hours working in the community every day, and is provided incentives based on performance [24]. An ANM is a qualified health provider who provides MNCH services (immunization, ANC, delivery, etc.) at the community and facility level. The medical officer is a medical doctor who leads the PHC team [12].

The study area included six blocks of the Bharuch and Narmada districts identified as high-priority blocks by the state government because of poor health and development indicators compared to other areas and relatively lower literacy of 65% and inhabited by a tribe called *Bhil* or *Vasava* [25–28].

Twenty-six PHCs (except two PHCs where ImTeCHO was implemented already as part of an earlier pilot) were eligible to be included in these six blocks, having a 100% rural population and with at least 45% of the population consisting of scheduled tribe caste. Four PHCs that had more than 10% villages without availability of internet through mobile data were excluded, considering the study’s need for at least a few minutes every day for intervention to work.

This study was conducted by SEWA Rural, which is a nongovernmental, voluntary grassroots organization located at the border of the study area in active partnership with the Department of Health and Family Welfare, Government of Gujarat, and an information technology (IT) partner, Argusoft India Ltd. The Department of Health and Family Welfare, Government of Gujarat provided written approval and formal notifications for conducting the study within existing public health system, and one of the senior state-level officers joined the team of investigators. No major changes were made to the design of the study after its commencement.

### Interventions

We used Medical Research Council (Swindon, UK) framework and user-centered design to develop and evaluate a complex health intervention in the form of a mobile-phone-technology–based job aid for the ASHAs and PHC staff with an aim to make their work easier and more effective [29]. A formative evaluation, as part of a pilot, was done in 45 villages in 2014 in a location outside the study area [22]. An online video is available that provides a short demonstration of the intervention [30].

Table 1 shows various mHealth strategies included in the application, and S1 Table shows details of service delivery in the intervention and control arms [23]. ASHAs were given Samsung Galaxy Star Pro (GT-S7262) android smart phone (cost US $86) with a postpaid data plan costing US $3 per month per ASHA. ASHAs registered all existing and new pregnant women and children under the age of two years in their villages using the ImTeCHO mobile phone application throughout the study period. Subsequently, the software prepared an entire schedule of home visits as per the national standards and sent reminders to ASHAs. Every day, ASHAs logged in to the application to review their schedule, conducted a home visit based on the task list, and completed and submitted a digital form during the home visits to close out the scheduled task. To emphasize key health and wellness messages, ASHAs showed short video clips on the mobile phone to family members during the home visits. The digital forms had checklists to screen for complications. The application had a decision support system to show probable diagnosis with risk stratification to ASHAs based on the entries made in the digital forms and a customized management plan that included tools to call emergency transport vehicles and suggested home-based remedies along with names and doses of drugs in compliance with national standards for the ASHA program. Additionally, ASHAs recorded details of the services, including immunization, delivered by the ANMs during monthly Village Health and Nutrition Days (VHNDs) in the ImTeCHO application as well. Based on the data entered by an ASHA throughout a month, her performance report was generated and shared with each ASHA on the first day of the subsequent month through the ImTeCHO application, and performance-based incentives were calculated automatically to ensure timely payment. The data transfer happened through the internet; however, data entry was possible even offline. Each medical officer was provided with a Samsung Galaxy Tab3V (cost US $165) tablet to use the ImTeCHO web interface. The medical officers and PHC staff used the web interface to track high-risk cases, view reports, and manage incentives and supplies.

**Table 1:** Intervention strategies of ImTeCHO.

	mHealth Strategy as Intervention Activity	Type of Exposure	Intervention Provider
A	Mobile phone as job aid to ASHA to increase coverage MNCH care		
	Longitudinal digital tracking of pregnant women and infants’ health status and services	Individual	ASHA
	Scheduling and activity planning in form of reminder to ASHAs to make home visits and mobilize for due services during VHND	Group	ASHA
	Decision support in form of digital checklist to encourage ASHAs to adhere to protocols during home visits. This included assessing and addressing barriers for behavior change at household level (e.g., birth preparedness, complication readiness)	Individual	ASHA
	Targeted client communication using multimedia to transmit targeted health information and improve counseling for behavior change communication	Group	ASHA
	Manage electronic health record of pregnant women and infants	Group	ASHA
	Notify stock levels and stock-out of health commodities	Group	ASHA
	Receive training content in form of multimedia files	Group	ASHA
B	Mobile phone as job aid to ASHA and ANM to facilitate care for mother, newborn, and child with complications		
	Decision support in form of digital checklist and inbuilt algorithms to screen and risk-stratify a case with complications	Individual	ASHA
	Referral coordination to facilitate referral to functional facility and emergency transport	Individual	ASHA
	Communication to ANM and medical officer once complicated case is identified by ASHA in form of SMS and notification alert on medical officer dashboard	Group	ASHA, ANM, medical officer
	Decision support in form of display of customized management guidelines on mobile phone and web interface to help ASHA and medical officer manage complicated cases	Individual	ASHA, medical officer
	A counselor using a helpline dashboard provided telemedicine services. The helpline dashboard enabled the counselor to remotely monitor health data and provide remote consultation for case management to ASHAs, pregnant women, and mothers	Individual	Helpline counselor
C	Web interface to provide timely information and tools to medical officer and PHC staff to facilitate monitoring and supporting ASHA program		
	Human resource management in form of list of health workforce cadres and monitor performance monitoring of ASHAs	Group	Medical officer
	Digital tracking of selected high-risk cases	Group	Medical officer
	Registration of birth and death events	Group	Medical officer
	Data synthesis and aggregation to provide monthly reports	Group	Medical officer
	Manage inventory and distribution of health commodities	Group	Medical officer
	Calculation and timely payment of incentive to ASHAs	Group	Medical officer
	Mass broadcast of motivational messages and training content to ASHAs using announcement feature	Group	Project team

**Abbreviations:** ANM, Auxiliary Nurse Midwife; ASHA, Accredited Social Health Activist; ImTeCHO, Innovative Mobile-phone Technology for Community Health Operations; MNCH, maternal, neonatal, and child health; PHC, Primary Health Center; SMS, Short Message Service; VHND, Village Health and Nutrition Day.

Complete enumeration of study population and the preintervention baseline survey in the study area was conducted from February, 2015 to July, 2015 [23]. SEWA Rural and government staff conducted training using audiovisual training tools and hands-on practice in groups from August, 2015 to January, 2016. The intervention was implemented from February, 2016 to January, 2017. The postintervention endline survey was conducted from February, 2017 to July, 2017, and its methodology was published as part of the protocol [23].

### Control arm (comparator)

The government and other providers continued to provide usual health services in the control area. A refresher training (three days) and one-time supply of commodities were provided to ASHAs from the control and intervention area [23].

### Participants

All health providers (ASHAs, ANMs, medical officers, and PHC support staff) belonging to PHCs in the intervention and control arms were the participants of the study. All pregnant women, neonates, and infants from study area were the participants. The mHealth facilitator and helpline (description in S1 Table) were only present in the intervention arm. The mHealth facilitators (one per 50 ASHAs) and one counselor at helpline were part of the project team from SEWA Rural [23].

### Management

The project team stationed at the headquarters included an implementation coordinator and project associate who supervised the team of mHFs and helpline counselor. It intervened in cases of emergencies, organized a training at the beginning of the trial, paid additional token monetary incentives to intervention ASHAs (ranging from US $4 to $11 monthly per ASHA based on performance), managed SIM cards, and coordinated with partner organizations.

The project team did not conduct community mobilization, regular field-level supervision, or separate meetings with ASHAs other than participating in regular monthly PHC meetings. In addition, they also did not deliver any health services at the community level or provide any other kind of support to ASHAs not listed in S1 Table or ongoing provision of commodities after initial training.

The district- and state-level government officers actively participated in proposal development workshops, provided feedback about the software, participated in regular review meetings, and led implementation jointly with project management team. The IT partner carried out regular software maintenance and ensured timely resolution of any related issues on an ongoing basis.

### Outcomes

The primary and secondary outcomes of interest were coverage of various health services to be delivered or facilitated by ASHAs, which were expected to change as a result of ImTeCHO intervention, along with process indicators to measure fidelity to the intervention. The two primary outcomes of interest were (i) the proportion of neonates/mothers visited at home by ASHA at least two times within one week after delivery, and (ii) modified ASHA-centric composite coverage index (MACCI). The details of MACCI have been published elsewhere [23]. See S2 Text for the definition and rationale for the choice of primary outcomes of interests.

The ImTeCHO program data were used to obtain results of the process indicators to assess adherence to the intervention. The definitions of process indicators and secondary outcomes of interest were included in the published protocol [23]. There were no changes in trial outcomes after its commencement.

### Recruitment of respondents, and measurement of outcomes

An endline household survey was conducted from February, 2017 to July, 2017 to measure the primary as well as secondary outcomes of interest. The household survey tools used standard methodology for the district-level health and facility survey (DLFHS-4) [31]. There were two kinds of respondents.

1. For maternal and newborn health outcomes (respondent type A): All women who were natives of the study villages and were mothers of a one- to four-month–old infant at the time of survey were eligible. Women whose infants died before the survey were excluded.
2. For child health outcomes (respondent type B): All women who were natives of study villages and were mothers of a six- to eight-month–old infant at the time of survey were considered eligible. Women whose infants died before the survey were excluded.

All pregnancies, their outcomes, and infant and maternal deaths from the entire study area were counted prospectively from 2016 onwards as part of ongoing pregnancy and mortality surveillance. The information from pregnancy registration and mortality surveillance system was used to identify and enroll type A and type B respondents for the endline survey.

The data were entered into an in-house–developed data entry software, different from the ImTeCHO application, through smart phones that had inbuilt provisions to avoid illogical data entries and missing data. A data administrator checked for any inconsistencies, which were clarified with data collectors and corrected. The endline survey was conducted jointly by an independent evaluation team comprising trained data collectors, who worked separately and were not involved in the implementation activities, and an independent research organization, Population Research Centre (PRC), Vadodara, Gujarat. PRC validated data on a randomly selected 3% of respondents through household survey using a truncated questionnaire.

### Sample size

We calculated sample size for both the primary outcomes of interest and used the larger one. Eleven PHCs each in the intervention and control clusters were included to address both questions [23]. Based on the results of an evaluation of an earlier pilot conducted prior to the trial, we assumed that ASHAs would visit 46% of neonates/mothers at least twice at home within first week of delivery and MACCI would be 36% in control area [32]. We assumed there would be 25 ASHAs associated with one PHC. We assumed intraclass correlation (ICC) to be 0.02 in light of no existing information available regarding ICC. We estimated that there might be loss of one cluster per arm and three ASHAs per PHC over the course of the study period. For a power of 80% and 5% two-sided significance level, we estimated that six PHCs/clusters (150 ASHAs) per arm were required to detect 20% absolute improvement in the proportion of neonates/mothers who received at least two postnatal home visits within first week of delivery by an ASHA in the intervention arm compared to the control arm at endline survey. Similarly, we estimated that 11 PHCs/clusters (275 ASHAs) per arm were required for detecting 15% absolute improvement in MACCI in the intervention arm compared to the control arm at endline survey.

### Randomization

An independent senior statistician not involved in study implementation carried out randomization and assigned 11 PHCs to the intervention and 11 to the control arm. MACCI was calculated for each PHC after the baseline survey; the mean value of MACCI was calculated, which was then used as a cutoff to allocate each PHC into one of the two strata. The PHCs in each of the two strata were randomly allocated to the intervention and control groups in a 1:1 ratio to ensure that MACCI was similar at the baseline for the intervention and control arms using the software nQuery. Blinding was not possible because this was a community-based intervention. All pregnant women and infants identified through complete enumeration and identification of new pregnant women along with their newborn babies throughout the study period in the study area were eligible to participate.

### Ethical review

The Multi-institutional Ethics Committee (Mumbai), Institutional Ethics Committee of SEWA Rural, and Ethics Review Committee of WHO approved the study. All respondents provided written informed consent to trained data collectors prior to the household survey. Every respondent who participated in the endline survey was assigned a unique identification number, and personally identifiable information was removed. Standard security and data encryption practices were used by the ImTeCHO application system for accepting, transmitting, processing, and storing data.

The results were reported in accordance with CONSORT statement for reporting cluster-randomized trials (see S2 Table).

### Statistical methods

Primary analysis was intention to treat (ITT) and secondary analysis was per protocol (PP) at the ASHA level. A Generalized Estimating Equation (GEE) was used to account for clustering and to adjust for maternal age, caste, parity, poverty status, and education. For ITT analysis, all enrolled respondents were included in the analysis irrespective of whether they received the treatment/intervention or not. However, there is a common local custom for a large proportion of pregnant women to move out of their own homes and spend the last trimester and first few weeks after delivery at their maternal village, which might be out of the study cluster and might not have been completely exposed to the treatment arm. Therefore, PP analysis was done for indicators related to antenatal and postnatal services (respondent type A).

Two-level random-effects regression models were applied for each of the primary and secondary outcomes. For continuous and categorical outcomes, cluster-level means and proportion were used, respectively. In the case of proportion, the mean of proportion by cluster was calculated and reported. Analysis was done at the ASHA level. The differences with effect size and *p*-value for each outcome were also reported. The effect size was adjusted for maternal age, caste, parity, below poverty line (BPL) card status, and women’s education level. The denominator for outcomes related to pregnancy and postnatal services included respondents of type A. The denominator for outcomes related to postneonatal services included respondents of type B. STATA 13.0 was used for data analysis.

### Trial registration

This trial was registered with the Clinical Trial Registry of India (www.ctri.nic.in, registration number CTRI/2015/06/005847, date of registration 3 June, 2015).

### Data safety monitoring board

The data safety and monitoring board, comprising one scientist, one ethicist, one community representative, and one statistician, monitored the trial during annual meetings.

## Results

**Fig 1:**
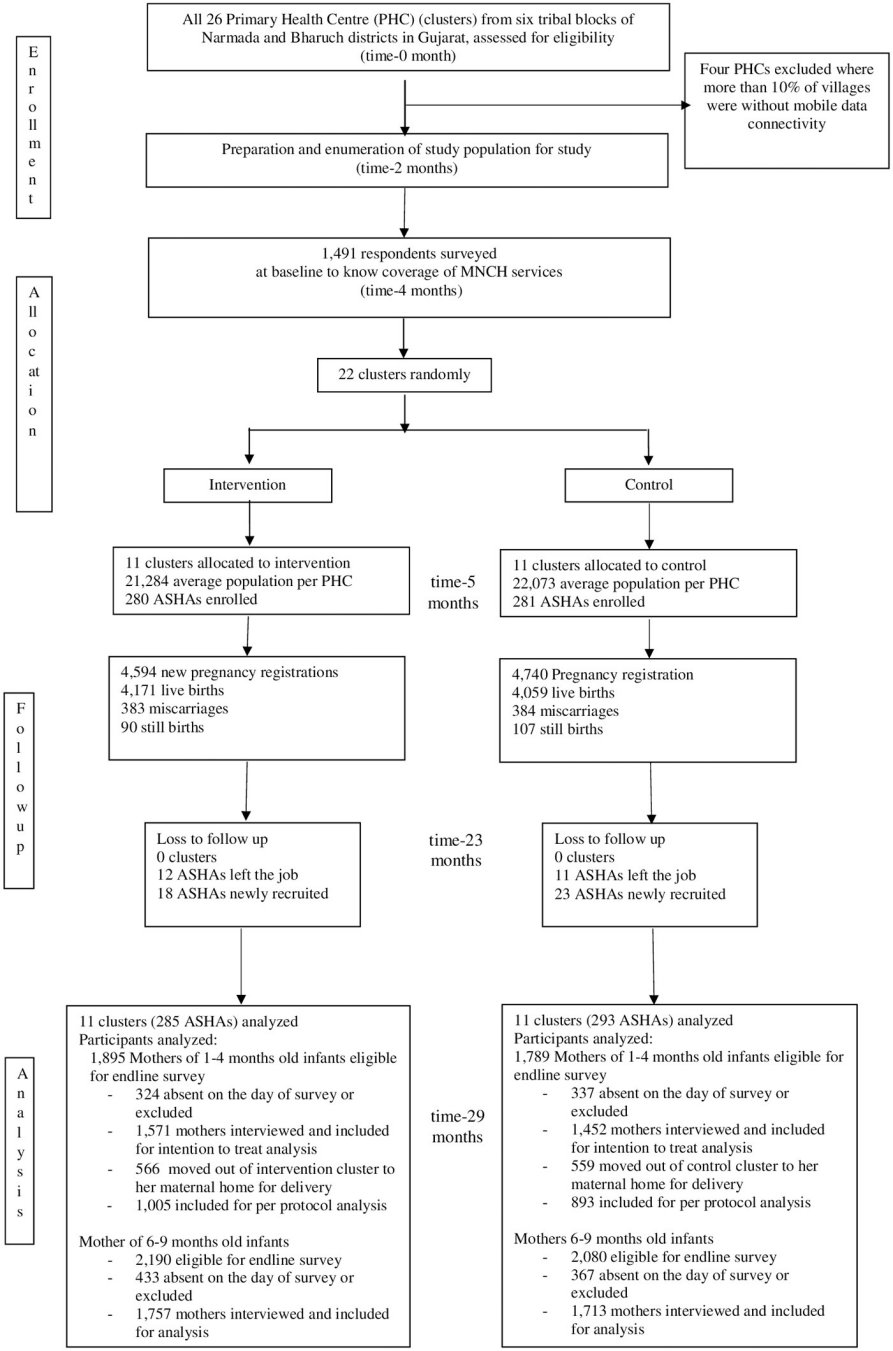
Trial profile. ASHA, Accredited Social Health Activist; MNCH, maternal, neonatal, and child health; PHC, Primary Health Center.

Fig 1 shows trial profile. The 11 randomly allocated intervention clusters from six tribal blocks had a population of 234,124 with 280 ASHAs, and the 11 control clusters had a population of 242,803 with 281 ASHAs at baseline. All 22 clusters were followed until the end of the trial. 9,334 new pregnancies and 8,230 live births were recorded in the study area during the implementation period from February, 2016 to January, 2017.

During the baseline survey, 1,419 respondents were interviewed (Table 2). The sociodemographic profile, coverage of MNCH services, and cluster-level characteristics were largely similar in the intervention and control arms at the time of the baseline survey in 2015 except for caste (79.0% women from scheduled tribe in intervention clusters versus 87.3% in control), place of delivery (73.9% women delivered at a hospital in the intervention clusters versus 85.0% in control), low standard of living index (37.6% in intervention versus 28.6% in control), and at least two home visits within first week (19% in the intervention clusters versus 14% in control). There were more vacancies in ASHA positions at baseline in the control clusters (7.5% vacancies in control clusters versus 5.0% in intervention clusters). All 11 PHCs in the control clusters had a functional delivery facility compared to seven PHCs in the intervention clusters.

**Table 2:** Characteristics of mothers, coverage of MNCH services, and cluster-level characteristics at baseline.

		Intervention Arm	Control Arm
Cluster-level characteristics			
Number of PHCs		11	11
Average population of PHC/cluster		21,284	22,073
Number of subcenters		91	85
Number of villages		274	255
Number of ASHAs		280	281
Number of ASHAs who had more than 10 years of formal education		170 (60.7%)	206 (73.3%)
Mean years of experience of ASHA		5.33 (3.11)	6.03 (2.96)
Mean age of ASHAs (years)		36 (7.35)	34 (7.31)
Proportion of vacant ASHA positions		14 (5.0%)	21 (7.5%)
Proportion of vacant ANM positions		6 (6.6%)	7 (8.2%)
Proportion of PHCs without a medical officer (MBBS or AYUSH)		0%	0%
Proportion of PHCs with functional delivery facility		4 out of 11 (63.6%)	11 out of 11 (100%)
Proportion of villages that are not accessible by road during monsoon		12 (4.3%)	14 (5.4%)
Sociodemographic characteristics			
Number of respondents at baseline		785	706
Maternal education	No formal education	166 (21.1%)	109 (15.4%)
	1–8 standard	354 (45.1%)	312 (44.2%)
	>8th standard	265 (33.8%)	285 (40.4%)
Gravida	1	256 (32.6%)	239 (33.9%)
	2	292 (37.2%)	287 (40.7%)
	≥3	237 (30.2%)	180 (25.5%)
Maternal caste	Scheduled tribe	620 (79.0%)	616 (87.3%)
	Scheduled caste/other backward caste	113 (14.4%)	41 (5.8%)
	Other	52 (6.6%)	49 (6.9%)
Age of the mother, mean years		24.4 (3.63)	24.3 (3.46)
Place of delivery	Hospital	580 (73.9%)	600 (85.0%)
	Home	194 (24.7%)	96 (13.6%)
	On the way	11 (1.4%)	10 (1.4%)
Standard of living index	Low	295 (37.6%)	202 (28.6%)
	Medium	260 (33.1%)	244 (34.6%)
	High	230 (29.3%)	260 (36.8%)
Coverage of MNCH services during last pregnancy, postnatal period, and early infancy			
Number of respondents at baseline		785	706
Early registration of pregnancy		632 (80.5%)	588 (83.3%)
Full antenatal check-up* during the last pregnancy		394 (50.2%)	369 (52.3%)
Four or more ANC examinations by ANM/doctor including at least one examination in last trimester		622 (79.2%)	576 (81.6%)
At least one injection ofTT during the last pregnancy		771 (98.2%)	694 (98.3%)
ASHA visited at home at least three times during last pregnancy, including at least one visit during last trimester		195 (24.8%)	163 (23.1%)
Delivered at an institution/hospital		580 (73.9%)	600 (85.0%)
ASHA present during delivery		267 (34.0%)	267 (37.8%)
Breastfed within 1 hour of birth		400 (51.0%)	345 (48.9%)
ASHA visited the mother and neonate at home within 24 hours of delivery (in case of home delivery) or within 24 hours of return to home from hospital in case of hospital delivery		394 (50.2%)	369 (52.3%)
ASHA visited the mother and neonate at their home at least twice within the first week of delivery		149 (19.0%)	99 (14.0%)
ASHA visited the mother and neonate at their home at least five times within the first month of delivery, of which at least two visits were made within first week of delivery		44 (5.6%)	37 (5.2%)
Developed neonatal complications within first month of last delivery		270 (34.4%)	285 (40.4%)
Developed neonatal complications within first month of last delivery and sought care from ASHA		68 (25.2%)	63 (22.1%)
Exclusive breastfeeding for the first six months after delivery		151 (19.2%)	95 (13.5%)
Received solid, semisolid, or soft foods during the previous day		630 (80.3%)	597 (84.6%)
Developed ARI/fever within last two weeks from the day of survey		237 (30.2%)	233 (33%)
Developed ARI/fever within last two weeks from the day of survey and sought care from ASHA		56 (23.6%)	42 (18.0%)
Suffered from diarrhea within last two weeks from the day of survey		98 (12.5%)	94 (13.3%)
Suffered from diarrhea within last two weeks from the day of survey and received ORS from ASHA		8 (8.2%)	6 (6.4%)
Received all three doses of pentavalent vaccine		558 (71.1%)	533 (75.5%)
MACCI**		31%	31%

Data are n (%) and mean (SD) unless specified otherwise.

*Full antenatal check-up defined as at least four antenatal examination by ANM/doctor, at least one injection of TT, and consumption of at least 100 tablets of iron and folic acid during the pregnancy.

**Data presented are a composite score with range from 0 to 100. Please see formula used to calculate composite index in S2 Text. ANC, Antenatal care; ANM, Auxiliary Nurse Midwife; ARI, Acute Respiratory Infection; ASHA, Accredited Social Health Activist; AYUSH, Ayurveda, Yoga & Naturopathy, Unani, Siddha, Sowa Rigpa, and Homoeopathy; MACCI, modified ASHA-centric composite coverage index; MBBS, Bachelor of Medicine, Bachelor of Surgery; MNCH, maternal, neonatal, and child health; ORS, Oral Rehydration Solution; PHC, Primary Health Center; TT, Tetanus Toxoid.

Throughout of the study period, uptake and adherence to the ImTeCHO mobile phone application was satisfactory among ASHAs (Table 3). The ASHAs logged into the ImTeCHO mobile phone application on 85% of days and marked 79% of all scheduled home visits as complete using the application. However, the PHC staff and PHC medical officers’ use of ImTeCHO web interface was less than expected (Table 3). Throughout the study period, 17 mobile phones (285 ASHAs) were lost, stolen, or irreparably damaged. 96 software issues were escalated to and addressed by Argusoft India Ltd over 12 months. Three out of 11 PHCs disbursed ASHA incentives as per the ImTeCHO system. The incremental cost per birth was US $54. The results of the cost-effectiveness study, nested within this trial, will be reported in a separate publication.

**Table 3:** Adherence to intervention by ASHAs and PHC staff.

Adherence to ImTeCHO Intervention by ASHAs	Mean for 12 Months (SD)
Proportion of days ASHAs logged in ImTeCHO mobile phone (mean for 12 months)*	85% (2.8)
Proportion of scheduled home visit reminders marked completed by ASHAs in ImTeCHO mobile phone application (mean for 12 months)*	78% (3.0)
Proportion of deliveries reported in ImTeCHO by ASHAs on the day of delivery	41% (5.1)
**Adherence to ImTeCHO Intervention by the PHC Staff**	
Proportion of days the PHC medical officer logged in ImTeCHO web interface (mean for 12 months)*	24% (9.6)
Proportion of scheduled tasks** marked completed in ImTeCHO web interface by the PHC medical officers (mean for 12 months)*	34% (24.9)
Stock-out rate as reported by ASHAs using ImTeCHO application (mean for 12 months)*	11% (2.7)

*Percentages are based on cluster averages. First, mean for every PHC was calculated based on the value for each ASHA. Then, mean for entire intervention arm was calculated based on value for all 11 PHCs for every month throughout the study duration. Subsequently, mean for entire study duration (12 months) was calculated.

**Reminders for complicated cases, verbal autopsy in case of death, stock-outs, payment of incentives to ASHAs.

**Abbreviations:** ASHA, Accredited Social Health Activist; ImTeCHO, Innovative Mobile-phone Technology for Community Health Operations; PHC, Primary Health Center.

At the time of the end line survey, 3,023 type A respondents and 3,470 type B respondents were interviewed and analyzed. Most of the respondents were tribal (5,571, 85.8%), and reported having a government-issued certificate for living BPL (4,916, 75.7%). 868 (13.3%) women had not received any formal education. All 22 PHCs (578 ASHAs) were included in the analysis. The proportion of neonates who were visited at least twice during the first week of delivery and MACCI was significantly higher in the intervention arm compared to the control arm in both ITT and PP analysis (Table 4). In ITT analysis, the coverage of at least two home visits within first week of birth was 32.4% in the intervention clusters, compared to 22.9% in the control clusters (adjusted effect size 10.2 [95% CI: 6.4, 14.0], *p* < 0.001). There were baseline differences in the place of delivery and coverage of at least two home visits within the first week of birth. After adjusting for baseline differences in place of delivery, the coverage of at least two home visits within the first week of birth remained significantly higher in the intervention clusters compared to the control clusters (adjusted effect size 10.2 [95% CI: 6.4, 13.9], *p* < 0.001). Similarly, the coverage of at least two home visits within the first week of birth remained significantly higher in the intervention clusters compared to the control clusters (adjusted effect size 6.6 [95% CI: 2.5, 11.7], *p* < 0.001) after adjusting for baseline differences for this primary outcome. The mean number of home visits within the first week of birth was 1.11 and 0.80 for the intervention and control clusters, respectively (adjusted effect size 0.34 [95% CI: 0.23, 0.45], *p* < 0.001). The mean number of home visits within the first month of birth was 2.70 and 2.00 for the intervention and control clusters, respectively (adjusted effect size 0.75 [95% CI: 0.47, 1.04], *p* < 0.001). The composite coverage index was 43.0% in the intervention clusters compared to 38.5% (adjusted effect size 4.9 [95% CI: 0.2, 9.5], *p* = 0.03) in the control clusters. Table 5 reports the results of secondary outcome indicators. Exclusive breastfeeding, home visitations during antenatal period, satisfactory counseling during antenatal period, early initiation of breastfeeding, ASHAs’ visitation at home within 24 hours of delivery (in case of home delivery) or within 24 hours of return to home from hospital in case of hospital delivery, satisfactory counseling during postnatal period, home visitations during postnatal period, newborn examination during home visits, and administration of Oral Rehydration Solution (ORS) in case of diarrhea were significantly higher in the intervention clusters compared to the control clusters. Care-seeking for antenatal and neonatal complications was also significantly higher in the intervention clusters compared to the control clusters. However, there were no differences in the rates of full ANC provision by the ANM, facility delivery, care-seeking from ASHA for pneumonia, and Diphtheria, Pertussis, Tetanus-3 (DPT-3) vaccination coverage. The concordance rate of responses between the data collectors and team of independent quality assessors from PRC was 89% during the endline survey.

**Table 4:** Primary outcomes of interest by study arm at endline survey.

	ITT						PP					
	Intervention, 11 clusters (285 ASHAs), mean (95% CI)	Control, 11 clusters (293 ASHAs), mean (95% CI)	Unadjusted effect size (95% CI)	*p*-value	Adjusted* effect size (95% CI)	*p*-value	Intervention mean (95% CI)	Control mean (95% CI)	Unadjusted effect size (95% CI)	*p*-value	Adjusted* effect size (95% CI)	*p*-value
At least two home visits by ASHA within first week after delivery**	32.4 (29.7, 35.1)	22.9 (20.2, 25.6)	9.5 (5.7, 13.3)	<0.001	10.2 (6.4, 14.0)	<0.001	39.7 (36.2, 43.3)	27.6 (23.9, 31.2)	12.2 (7.1, 17.3)	<0.001	12.4 (7.2, 17.5)	<0.001
MACCI**	43.0 (39.7, 46.3)	38.5 (34.5, 41.6)	4.6 (0.2, 9.3)	0.057	4.9 (0.2, 9.5)	0.037	44.2 (40.9, 47.5)	39.1 (35.8, 42.4)	5.1 (0.4, 9.8)	0.033	5.3 (0.7, 10.0)	0.024

*Adjusted for maternal age, education, parity, caste, and BPL card.

**Data are weighted mean of clusters (95% CI).

**Abbreviations:** ASHA, Accredited Social Health Activist; BPL, below poverty line; ITT, intention to treat; MACCI, modified ASHA-centric composite coverage index; PP, per protocol.

**Table 5:** Comparison of coverage and quality of maternal and neonatal services among type A respondents (mothers of infants who were one to four months old) and young infant care services among type B respondents (mothers of infants who were six to nine months old) by study arm at endline survey.

	ITT						PP					
	Intervention, 11 clusters (285 ASHAs), mean (95% CI)*	Control, 11 clusters (293 ASHAs), mean (95% CI)*	Unadjusted effect size	*p*-value	Adjusted** effect size	*p*-value	Intervention, 11 clusters (285 ASHAs), mean (95% CI)*	Control, 11 clusters (293 ASHAs), mean (95% CI)*	Unadjusted effect size	*p*-value	Adjusted** effect size	*p*-value
Coverage and quality of maternal health services during pregnancy (type A respondents)												
First antenatal examination by ANM/doctor during first trimester	80.8 (78.6, 83.0)	82.8 (80.6, 85.1)	−1.9 (−5.1, 1.2)	0.217	−2.3 (−5.3, 0.8)	0.151	78.8 (76.1, 81.5)	80.6 (77.8, 83.4)	−1.8 (−5.7, 2.1)	0.367	−1.9 (−5.7, 1.9)	0.329
Mothers who were visited at home by ASHA at least three times during last pregnancy, including at least one visit during last trimester	53.7 (50.4, 57.0)	39.2 (35.8, 42.5)	14.5 (9.8, 19.2)	<0.001	15.7 (11.0, 20.4)	<0.001	58.8 (54.7, 62.9)	40.8 (36.7, 44.9)	18.0 (12.2 to 23.8)	<0.001	18.9 (13.1, 24.7)	<0.001
Satisfactory ANC counseling regarding at least five out of six desired practices, including birth planning and complication readiness, expected date of delivery, danger signs of pregnancy, examination by ANM/doctor, institutional delivery, and early essential newborn practices	51.0 (47.8, 54.2)	35.4 (32.1, 38.6)	15.6 (11.0, 20.2)	<0.001	16.9 (12.3, 21.5)	<0.001	55.1 (51.1, 59.1)	37.2 (33.2, 41.2)	17.9 (12.2 to 23.6)	<0.001	19.0 (13.3, 24.7)	<0.001
Knowledge about danger signs of pregnancy (able to state at least three danger signs)	6.3 (4.7, 7.7)	7.2 (5.8, 8.6)	−0.9 (−2.9, 1.1)	0.385	−0.5 (−2.5, 1.4)	0.595	5.2 (3.7, 6.8)	6.9 (5.3, 8.5)	−1.7 (−3.9, 0.6)	0.146	−1.3 (−3.5, 8.4)	0.230
At least four antenatal check-ups by ANM or doctor with at least one check-up in last trimester	89.5 (87.6, 91.3)	88.7 (86.8, 90.6)	0.8 (−1.8, 3.4)	0.564	1.2 (−1.3, 3.7)	0.342	88.5 (86.1, 90.8)	88.6 (86.2, 90.9)	−0.1 (−3.4, 3.2)	0.940	0.7 (−2.5, 3.9)	0.679
Received at least one dose of injected TT during last pregnancy	98.2 (97.4, 98.9)	96.8 (96.0, 97.6)	1.3 (0.0, 2.4)	0.02	1.6 (0.5, 2.7)	0.005	98.0 (97.0, 99.0)	97.0 (95.9, 97.9)	1.1 (−0.4, 2.5)	0.143	1.4 (−0.1, 2.8)	0.065
Consumed at least 100 IFA tablets during pregnancy	66.1 (60.0, 72.2)	66.3 (63.3, 69.5)	1.4 (−2.9, 5.8)	0.52	2.10 (−2.2, 6.4)	0.341	65.8 (62.0, 69.6)	64.8 (61.0, 68.6)	1.0 (−4.4, 6.3)	0.719	1.5 (−3.9, 6.8)	0.587
Full ANC (3 ANC + 1 TT + 100 IFA tablets)	65.6 (62.5, 68.7)	63.6 (60.5, 66.7)	2.0 (−0.2, 6.4)	0.377	2.7 (−1.7, 7.0)	0.228	64.8 (61.0, 68.6)	63.6 (59.8, 67.5)	1.2 (−4.2, 6.6)	0.673	1.6 (−3.7, 7.0)	0.550
Delivery at hospital	83.2 (80.4, 85.9)	84.9 (82.1, 87.6)	−1.7 (−5.7, 2.2)	0.402	−1.4 (−4.9, 2.1)	0.432	80.1 (76.6, 83.6)	83.0 (79.5, 86.5)	−3.0 (−7.9, 2.0)	0.248	−2.6 (−7.2, 2.0)	0.267
Coverage and quality of neonatal and maternal health services during postnatal period (type A respondents)												
Practice of newborn care immediately after delivery												
Early initiation (within 1 hour) of breastfeeding	68.6 (65.9, 71.2)	61.8 (59.2, 64.5)	6.7 (3.0, 10.5)	<0.001	7.8 (4.2, 11.4)	<0.001	70.8 (67.7, 74.0)	65.0 (61.8, 68.3)	5.8 (1.3, 10.3)	0.012	6.7 (2.3, 11.2)	0.003
Colostrum was fed	75.7 (73.2, 78.1)	74.6 (72.1, 77.1)	1.1 (−2.4, 4.6)	0.551	1.6 (, 1.71, 5.0)	0.334	75.9 (72.9, 78.9)	71.9 (68.9, 75.1)	3.9 (, 0.4, 8.3)	0.076	4.3 (0.1, 8.6)	0.047
Bathing delayed after first day	92.8 (91.0, 94.7)	91.1 (89.2, 93.0)	1.7 (−0.9, 4.3)	0.200	2.2 (, 0.3, 4.8)	0.089	92.9 (90.5, 95.4)	89.7 (86.1, 93.2)	3.2 (0.2, 6.6)	0.068	3.5 (0.2, 6.8)	0.039
No prelacteal feed administered	92.6 (89.5, 92.9)	92.5 (90.9, 94.2)	1.4 (−1.0, 3.7)	0.259	2.3 (0.0, 4.5)	0.053	94.2 (92.4, 96.0)	92.0 (90.1, 93.8)	2.3 (−0.3, 4.8)	0.082	2.7 (0.1, 5.2)	0.039
At least two home visits by ASHA within first week after delivery	32.4 (29.7, 35.1)	22.9 (20.2, 25.6)	9.5 (5.7, 13.3)	<0.001	10.2 (6.4, 14.0)	<0.001	39.7 (36.2, 43.3)	27.6 (23.9, 31.2)	12.2 (7.1, 17.3)	<0.001	12.4 (7.2, 17.5)	<0.001
Number of ASHA home visits in first week	1.11 (1.03 to 1.19)	0.80 (0.72 to 0.88)	0.31 (0.20 to 0.42)	<0.001	0.34 (0.23 to 0.45)	<0.001	1.33 (1.22, 1.43)	0.94 (0.84, 1.04)	0.39 (0.24, 0.53)	<0.001	0.40 (0.25, 0.55)	<0.001
ASHA visited at home within 24 hours of delivery (in case of home delivery) or within 24 hours of return to home from hospital in case of hospital delivery	55.4 (52.2, 58.6)	43.5 (40.2, 46.7)	9.5 (5.7, 13.3)	<0.001	13.3 (8.8, 17.8)	<0.001	64.2 (60.1, 68.3)	50.2 (46.0, 54.4)	14.1 (8.1, 19.9)	<0.001	14.6 (8.7, 20.4)	<0.001
ASHA visited at home at least five times within first month after last delivery, and at least two of those visits were within first week after delivery	20.7 (18.5, 23.0)	15.0 (12.6, 17.2)	5.8 (2.5, 9.0)	<0.001	6.4 (3.2, 9.6)	<0.001	25.8 (22.8, 28.8)	17.5 (14.4, 20.6)	8.3 (4.0, 12.6)	<0.001	8.6 (4.2, 12.9)	<0.001
Number of ASHA home visits in first month	2.70 (2.50 to 2.90)	2.00 (1.79 to 2.20)	0.70 (0.41 to 0.99)	<0.001	0.75 (0.47 to 1.04)	<0.001	3.25 (3.00, 3.52)	2.24 (1.97 to 2.52)	1.00 (0.62 to 1.40)	<0.001	1.04 (0.65 to 1.43)	<0.001
Satisfactory counseling regarding at least five out of nine desired newborn care practices, including exclusive breastfeeding, proper attachment for breastfeeding, keeping baby warm by covering in cloth, delaying first bath, KMC, caring for umbilical cord, always washing hands before handling baby, vaccination, and danger signs of newborn	52.4 (46.5, 58.4)	37.4 (31.5, 43.4)	16.5 (12.0, 21.1)	<0.001	18.1 (13.5, 22.6)	<0.001	62.6 (58.4, 66.9)	41.7 (37.4, 46.0)	20.9 (14.9, 27.0)	<0.001	21.8 (15.8, 27.9)	<0.001
ASHA performed satisfactory newborn examination involving at least three out of five recommended examinations, including measuring temperature of newborn, weighting newborn, examining skin and umbilicus, washing hands before examining, and observing breastfeeding the baby in her presence	34.1 (31.2, 37.1)	20.2 (17.2, 23.1)	14.0 (9.8, 18.2)	<0.001	14.8 (10.6, 19.1)	<0.001	42.3 (38.2, 46.4)	24.0 (19.9, 28.1)	18.2 (12.5, 24.0)	<0.001	18.4 (12.7, 24.2)	<0.001
Knowledge about danger signs of newborn (able to correctly state at least three danger signs of newborn)	24.8 (22.5, 27.1)	20.1 (17.7, 22.4)	4.7 (1.4, 8.0)	0.006	5.1 (1.8, 8.3)	0.003	23.3 (20.6, 25.9)	18.7 (15.9, 21.5)	4.5 (0.7, 8.4)	0.022	4.8 (1.0, 8.6)	0.014
Mother/family practiced following at home during first month after delivery												
KMC	15.8 (13.6, 18.0)	16.7 (14.5, 18.9)	−0.9 (−4.1, 2.2)	0.541	−0.3 (−3.4, 2.8)	0.834	16.9 (14.2, 19.6)	16.4 (13.6, 19.1)	0.5 (−3.3, 4.4)	0.797	1.1 (−2.8, 4.9)	0.591
Did not apply anything on cord	78.9 (76.3, 81.6)	78.5 (75.8, 81.2)	0.5 (−3.4, 4.3)	0.817	1.1 (−2.8, 4.9)	0.589	81.1 (77.9, 84.4)	78.1 (74.8, 81.3)	3.0 (−1.5, 7.6)	0.192	3.2 (−1.3, 7.8)	0.169
Washed hands before handling baby	28.4 (25.7, 31.1)	24.9 (22.1, 27.6)	3.6 (−0.3, 7.4)	0.069	3.3 (−0.5, 7.2)	0.095	29.4 (26.5, 32.5)	23.8 (20.7, 26.9)	5.6 (1.3, 10.0)	0.011	5.8 (1.4, 10.2)	0.010
Exclusive breastfeeding in last 24 hours	58.3 (55.2, 61.5)	48.0 (44.8, 51.2)	10.3 (5.9, 14.7)	<0.001	11.6 (7.5, 15.7)	0.000	64.4 (60.9, 67.8)	48.7 (45.2, 52.3)	15.6 (10.7, 20.6)	<0.001	16.3 (11.5, 21.0)	<0.001
Care-seeking for maternal and neonatal complications (type A respondents)												
Suffered from at least one antenatal complication during last pregnancy	49.8 (46.5, 53.1)	52.8 (49.4, 56.1)	−3.1 (−6.9, 0.7)	0.114	−4.1 (−7.8, −0.4)	0.031	47.7 (44.3, 50.9)	49.8 (46.3, 53.2)	−2.1 (−6.9, 2.7)	0.388	−2.5 (−7.3, 2.2)	0.294
Sought help from ASHA for antenatal complication	49.9 (46.0, 53.9)	42.5 (38.5, 46.4)	7.5 (1.9, 13.0)	0.009	10.0 (4.7, 15.4)	<0.001	56.5 (51.6, 61.5)	47.8 (42.9, 52.9)	8.7 (1.6, 15.7)	0.016	10.7 (3.7, 17.6)	0.003
Sought care from qualified health worker	73.5 (70.8, 76.9)	77.3 (72.0, 82.5)	−3.8 (−8.5, 0.9)	0.119	−3.7 (−8.3, 1.0)	0.122	70.9 (66.4, 75.3)	76.4 (71.8, 80.9)	−5.6 (11.9, 8.5)	0.089	−4.7 (−10.9, 1.7)	0.148
Suffered from at least one postnatal complication within six weeks of last delivery	26.1 (23.8, 28.3)	28.0 (25.7, 30.3)	−1.9 (−5.1, 1.4)	0.255	−2.4 (−5.7, 0.8)	0.140	26.9 (24.1, 29.5)	25.1 (22.2, 28.0)	1.8 (−2.2, 5.7)	0.377	1.1 (−2.9, 5.1)	0.592
Sought care from qualified health worker	52.3 (47.2, 57.4)	54.3 (49.2, 59.4)	−2.0 (−9.2, 5.2)	0.583	−3.8 (−10.9, 3.4)	0.302	49.5 (43.2, 55.8)	56.7 (50.0, 63.6)	−7.3 (16.5, 19.9)	0.124	−8.7 (−18.0, 0.1)	0.067
Sought help from ASHA for postnatal complication	29.7 (25.2, 34.1)	24.1 (19.7, 28.6)	5.5 (−0.7, 11.8)	0.084	7.3 (1.1, 13.6)	0.022	33.3 (27.7, 39.0)	27.0 (20.9, 33.2)	6.3 (−2.1, 14.7)	0.140	8.1 (−0.3, 16.4)	0.059
Mothers who suffered from at least one serious complication during last pregnancy and sought care from a qualified health personnel	73.1 (69.4, 76.9)	77.6 (73.9, 81.3)	−4.5 (−9.8, 0.8)	0.095	−4.0 (−9.2, 1.2)	0.135	70.8 (65.9, 75.7)	77.6 (72.6, 82.5)	−6.7 (13.7, 0.2)	0.058	−5.6 (−12.5, 1.4)	0.118
Mothers who suffered from at least one serious complication within 6 weeks of last delivery and sought care from a qualified health personnel	41.2 (33.4, 48.9)	53.2 (45.5, 60.9)	−12.1 (−22.9, −1.1)	0.030	−12.8 (−23.4, −2.0)	0.021	41.1 (32.9, 49.3)	61.1 (51.6, 70.6)	−20.0 (−32.5, −7.5)	0.002	−20.6 (33.1, 8.2)	0.001
Suffered from at least one newborn complication within 2 weeks of last delivery	28.8 (26.4, 31.0)	30.8 (26.4, 31.0)	−2.1 (−5.5, 1.2)	0.209	−1.8 (−5.1, 1.6)	0.308	28.5 (25.7, 31.3)	28.2 (25.2., 31.2)	0.3 (−3.8, 4.4)	0.883	0.4 (−3.7, 4.5)	0.835
Sought help from ASHA for newborn complication	32.6 (28.0, 37.3)	26.0 (21.4, 30.6)	6.7 (0.1, 13.1)	0.046	9.2 (2.8, 15.6)	0.005	39.4 (33.5, 45.3)	28.4 (22.3, 34.5)	11.0 (2.5, 19.5)	0.011	13.3 (4.9, 21.6)	0.002
Sought care from qualified health worker for newborn complications	61.2 (56.8, 65.7)	65.3 (60.9, 69.8)	−4.1 (−10.4, 2.2)	0.201	−4.8 (−10.8, 1.1)	0.108	56.4 (51.0, 61.8)	58.8 (52.9, 64.7)	−2.4 (−10.4, 5.6)	0.555	−2.8 (−10.7, 5.0)	0.479
Low birth weight (≤2.0 kg) at the time of the birth	3.5 (2.3, 4.7)	6.6 (5.4, 7.8)	−3.1 (−4.8, −1.4)	0.000	−3.1 (−4.8, −1.3)	0.000	3.4 (2.0, 4.9)	6.8 (5.2, 8.4)	−3.4 (−5.5, −1.2)	0.002	−3.4 (−5.5, 1.2)	0.003
Mothers who provided KMC to their low-birth–weight babies (≤2.0 kg) within first month of last delivery	53.7 (39.5, 68.0)	52.0 (40.7, 63.3)	1.7 (−16.4, 19.8)	0.852	3.3 (−15.1, 21.8)	0.722	58.2 (40.8, 75.6)	55.1 (41.5, 68.6)	3.1 (−18.9, 25.2)	0.782	7.6 (−15.6, 30.9)	0.520
Harmed by any medicine given by ASHA	0.2 (0.0, 0.5)	0.7 (0.4, 1.0)	−0.5 (−1.0, −0.1)	0.032	−0.5 (−0.9, −0.1)	0.045						
Counseling mothers of infants age six to nine months by ASHA for following (type B respondents)												
Counseled to initiate complementary food at six months	80.4 (77.6, 83.2)	70.5 (67.7, 73.4)	9.9 (5.9, 13.9)	<0.001	11.2 (7.2, 15.2)	<0.001						
Counseled to add oil, sugar, and jaggery to food	37.2 (34.4, 40.0)	27.5 (24.8, 30.3)	9.7 (5.7, 13.6)	<0.001	10.2 (.2, 14.1)	<0.001						
Informed of status of child on WHO growth chart (green, yellow or red) within last 3 months	21.1 (18.8, 23.4)	15.5 (13.2, 17.8)	5.6 (2.3, 8.8)	0.001	6.0 (2.9, 9.2)	<0.001						
Counseled to attend VHND within last 3 months	92.4 (90.5, 94.2)	87.2 (85.3, 89.0)	5.2 (2.6, 7.8)	<0.001	5.3 (2.7, 8.0)	<0.001						
Motivated mother to contact her in case the child suffers from diarrhea, fever, or pneumonia	70.7 (67.6, 73.8)	58.7 (55.7, 61.9)	11.9 (7.6, 16.3)	<0.001	13.0 (8.5, 17.4)	<0.001						
Nutritional outcomes among children age six to nine months (type B respondents)												
Practiced exclusive breastfeeding until just under 6 months of age	57.4 (54.1, 60.8)	45.1 (41.8, 48.4)	12.4 (7.7, 17.0)	<0.001	13.4 (8.9, 17.9)	<0.001						
Child was weighed at least once during last 3 months	94.6 (92.9, 96.3)	88.8 (87.1, 90.5)	5.6 (3.4, 8.3)	<0.001	6.0 (3.6, 8.4)	<0.001						
Child who received solid, semisolid, or soft foods during previous day	86.6 (84.8, 88.4)	84.7 (82.9, 86.5)	1.9 (, 0.6, 4.5)	0.141	2.1 (, 0.4, 4.6)	0.097						
Child was fed solid, semisolid, or soft food at least twice within last 24 hours (minimum meal frequency)	74.5 (72.0, 77.0)	73.9 (71.4, 76.4)	0.7 (, 2.9, 4.2)	0.719	0.4 (, 2.9, 3.8)	0.815						
Child was fed solid, semisolid, or soft food with added oil, jaggery, or sugar at least once during previous day	9.8 (8.2, 11.4)	9.6 (7.9, 11.2)	0.2 (, 2.1, 2.6)	0.833	−0.3 (−2.5, 1.8)	0.753						
Mother knew status of child on WHO growth chart (green, yellow, or red)	18.4 (16.0, 20.8)	17.7 (15.3, 20.0)	0.7 (, 2.6, 4.1)	0.669	2.0 (−1.2, 5.2)	0.226						
Health seeking and vaccination among children age six to nine months (type B respondents)												
Visited ANM or doctor at least once within last 3 months (at VHND or any health facility)	93.9 (92.4, 95.5)	89.1 (87.5, 90.7)	4.8 (2.6, 7.1)	<0.001	5.4 (3.2 to 7.6)	<0.001						
Mother knew that she could contact ASHA for help in case child suffers from diarrhea, fever, or pneumonia	79.9 (77.0, 82.9)	70.5 (67.6, 73.4)	9.4 (5.3, 13.4)	<0.001	10.1 (6.0 to 14.2)	<0.001						
Received all three doses of pentavalent vaccines	73.0 (70.3, 75.8)	73.6 (70.9, 76.4)	0.6 (, 3.3, 4.5)	0.763	1.1 (−2.7, 4.9)	0.589						
Suffered from diarrhea within last two weeks	16.3 (14.2, 18.4)	16.3 (14.2, 18.4)	−0.6 (−3.6, 2.4)	0.699	−0.5 (−3.4, 2.5)	0.766						
Child received ORS	30.2 (24.9, 35.5)	24.2 (18.9, 29.4)	6.1 (, 1.3, 13.5)	0.108	8.0 (0.6, 15.4)	0.033						
Child received ORS and ORS was supplied by ASHA	19.6 (15.1, 24.0)	13.2 (8.8, 17.6)	6.3 (0.1, 12.6)	0.048	6.4 (0.1, 12.6)	0.047						
Suffered from pneumonia/fever within last two weeks	35.3 (32.8, 37.8)	31.2 (28.8, 33.7)	4.1 (0.5, 7.6)	0.002	3.6 (0.1, 7.1)	0.047						
Sought help from ASHA for pneumonia/fever	19.9 (16.7, 23.3)	18.1 (14.6, 21.6)	1.8 (, 3.0, 6.6)	0.453	2.4 (, 2.4, 7.2)	0.326						

**Abbreviations:** ANC, Antenatal care; ANM, Auxiliary Nurse Midwife; ASHA, Accredited Social Health Activist; IFA, iron–folic acid; ITT, intention to treat; KMC, Kangaroo Mother Care; ORS, Oral Rehydration Solution; PP, per protocol; TT, Tetanus Toxoid; VHND, Village Health and Nutrition Day.

There were 4,171 live births in intervention clusters and 4,059 in control clusters during 1 year of the study period. There were 233 infant deaths in intervention clusters and 236 in control clusters. 90 stillbirths occurred in intervention clusters, and 107 occurred in control clusters. The number of neonatal deaths was 104 in intervention clusters and 102 in control clusters.

## Discussion

The coverage and quality of many of the MNCH health services improved in the intervention arm compared to the control arm. Additionally, the mHealth strategies provided support to the ASHAs and encouraged them to adhere to protocols. The targeted client communication in the form of short video clips were found to be effective. The strategies involving longitudinal digital tracking and scheduling resulted in an improvement in the coverage of recommended number of home visits by ASHAs during antenatal and postnatal periods. Furthermore, there was an improvement in the quality of home visits with regards to behavior change communication and clinical examination, early initiation of and exclusive breastfeeding, care-seeking from ASHAs for antenatal and neonatal complications, and use of ORS for diarrhea. The uptake of the ImTeCHO intervention was satisfactory among ASHAs as reflected in high login and task completion rate, whereas it was lower than expected among the PHC staff. As expected, ImTeCHO intervention was not found to be effective towards increasing coverage of MNCH health services in which the ASHA was not the primary provider and relied on other cadres or infrastructure of the health system (such as full ANC, vaccination, or institutional delivery).

### Contribution of study to existing literature

To our knowledge, this is one of the first randomized controlled trials of its kind that assessed effectiveness of multiple mHealth strategies implemented in a low- and middle-income country (LMIC) through frontline health workers in an existing public health system throughout the continuum of care to improve a wide range of MNCH outcomes among hard-to-reach populations using a robust research methodology. The ImTeCHO intervention targeted the entire scope of potential users of mHealth-based solutions, including health providers, beneficiaries, and health system managers. Published data have shown improvement in outcomes related to only one phase of RMNCH life cycle or use of only one mHealth strategy (most commonly Short Message Service [SMS]-based) or in controlled settings outside a government-supported public health system and targeted either beneficiary or health providers [10,33]. A study in rural Ethiopia, which did not involve random allocation of treatment, concluded that mHealth strategies, in the form of reminders through SMS to frontline health workers, resulted in an increase in the coverage of antenatal visits and facility delivery [34]. A randomized trial in Tanzania, conducted in a controlled setting by a nongovernmental organization, reported that mHealth strategies implemented during the antenatal period resulted in an increase in facility delivery [8]. Another randomized trial conducted in Bihar, India using similar mHealth strategies reported significant improvement in the coverage of a wide range of MNCH services delivered by frontline health workers; however, there were intensive efforts to strengthen the health system in addition to the deployment of the mHealth intervention [35]. Systematic reviews have stated a low quality of evidence and advocated randomized trials using robust research methodologies in uncontrolled settings [10,36–38]. Randomized trials in Ethiopia and Democratic Republic of Congo reported that a safe delivery mobile phone application as a training tool for health workers resulted in significant improvement in health workers’ knowledge and skills about neonatal resuscitation [39, 40].

ASHAs and PHC staff faced some challenges to adopt ImTeCHO on a consistent basis: first, to balance competing priorities in the context of various other programs. The uptake of ImTeCHO web application among PHC staff was less than expected, especially in light of ImTeCHO not being mainstreamed in the public health system in the state at the time of study and other competing priority public health programs; second, to deal with the perception of increase in workload due to introduction of ImTeCHO; third, to overcome technology problems involving internet coverage, hardware issues with mobile phones, and resolving bugs in the software in a timely manner. ASHAs and PHC staff from eight intervention PHCs did not use ImTeCHO’s incentive management system because they were concerned that digitization of incentive calculation might take away the flexibility of a paper-based system and might reduce the amount of incentive they might get. We established systems and processes to overcome these challenges by ensuring health system preparedness, effective change management, training, supportive supervision, motivation, and timely technology support. In addition to the existing components of ImTeCHO, the use of automated voice calls for targeted client communication, verification of data quality, digitization of birth and death registration, and expansion of the user base to involve other cadres of frontline health workers may help us further improve coverage of health services [41].

### Study strengths and limitations

Our study had a few strengths. First, we aligned programmatic objectives (i.e., increasing coverage of MNCH services) with the personal priorities of ASHAs (e.g., calculation of performance-based incentives using ImTeCHO for timely disbursement). Second, we took health workers’ legitimate concerns into consideration about the reasons for their suboptimal performance and used technology as a job aid (not job add) to make their tasks more efficient, effective, and easy. These approaches resulted in a higher uptake of technology-based interventions and improved coverage of MNCH services. Finally, we identified critical operational requirements that improved adherence to the intervention; this included supportive supervision, timely resolution of technology problems, and change management, including monetary and/or nonmonetary incentives depending on the context. Such operational requirements could be an integral part for an mHealth program to succeed at scale.

Our study also had some potential limitations. First, the outcomes were reported by the mothers, and there was a risk of recall bias. The mean number of days between date of birth and date of questionnaires completed during the endline survey was 116 days and 112 days for the intervention and control areas, respectively. However, standard data collection methodologies were used, and the methodology was the same across the study arms to negate any problems with recall. There was high concordance (89%) between the data collected by the data collectors and the team of independent quality assessors. Second, there was a higher proportion of women from the scheduled tribe and from the lowest standard of living index in the intervention arm. These differences were adjusted in the final model. Finally, the total duration of the intervention was 12 months, which might be considered short. The choice of duration of the intervention was affected by experiences of the earlier pilot, logistic concerns related to expected events in future such as upcoming state elections making survey activities difficult, and possible reorganization of PHCs resulting in reorganization of clusters. Some of the indicators showed improvement over the course of study period even in the control clusters (at least two home visits by ASHA during first week of delivery improved from 14.0% at baseline to 22.9% at endline). This might indicate overall strengthening of ASHA performance, and additional benefits from the intervention could be viewed in this context.

### Conclusions

In conclusion, our data show that mHealth strategies can improve the coverage of proven MNCH services, especially in hard-to-reach populations, if there is adequate supportive supervision, change management, and ongoing technology assistance to ensure satisfactory adherence to the intervention. Encouraged by the results of the study and the demonstrated feasibility in the existing public health system, the Gujarat state government is now leading the scale-up of the modified ImTeCHO intervention in the entire state. The scope of the intervention is being expanded to include other health domains and health cadres to prepare one integrated health IT platform. Many of the activities conducted by the project team are being taken over by another nonprofit organization, GVK EMRI, which is currently providing free emergency ambulance services across the state as part of a public–private partnership, thus improving the chances of a successful scale-up while minimizing the dilution of effectiveness of the intervention [42]. We recommend that it would be very useful to evaluate the scale-up after a longer period of implementation.

## Supporting information

S1 Table Service delivery by study arm. (DOCX)

S2 Table CONSORT 2010 checklist of information to include when reporting a cluster-randomized trial. (DOCX)

S1 Text Protocol. (DOC)

S2 Text Definition and rationale for primary outcomes. (DOCX)

## Abbreviations

ANC: Antenatal care
ANM: Auxiliary Nurse Midwife
ARI: Acute Respiratory Infection
ASHA: Accredited Social Health Activist
AYUSH: Ayurveda, Yoga & Naturopathy, Unani, Siddha, Sowa Rigpa, and Homoeopathy
BPL: below poverty line
DLFHS: district-level health and facility survey
DPT-3: Diphtheria, Pertussis, Tetanus-3
GEE: Generalized Estimating Equation
ICC: intraclass correlation
ICMR: Indian Council of Medical Research
ImTeCHO: Innovative Mobile-phone Technology for Community Health Operations
IT: information technology
ITT: intention to treat
KMC: Kangaroo Mother Care
LMIC: low- and middle-income country
MACCI: modified ASHA-centric composite coverage index
MBBS: Bachelor of Medicine, Bachelor of Surgery
mHealth: mobile-phone-technology–based health
MNCH: maternal, neonatal, and child health
ORS: Oral Rehydration Solution
PHC: Primary Health Center
PP: per protocol
PRC: Population Research Centre
SMS: Short Message Service
TT: Tetanus Toxoid
VHND: Village Health and Nutrition Day

## References

[1] Alkema L, Chou D, Hogan D, Zhang S, Moller A, Gemmill A, et al Global, regional, and national levels and trends in maternal mortality between 1990 and 2015, with scenario-based projections to 2030: a systematic analysis by the UN Maternal Mortality Estimation Inter-Agency Group. Lancet. 2016; 387: 462–74. 10.1016/S0140-6736(15)00838-7 26584737 PMC5515236

[2] United Nations Inter-agency Group for Child Mortality Estimation (UN IGME). Levels & Trends in Child Mortality: Report 2018, Estimates Developed by the UN Inter-agency Group for Child Mortality Estimation. New York, NY: United Nations Children’s Fund; 2018.

[3] Lassi ZS, Haider BA, Bhutta ZA. Community-based intervention packages for reducing maternal and neonatal morbidity and mortality and improving neonatal outcomes. Cochrane Database Syst Rev. 2010 11 10;(11):CD007754 10.1002/14651858.CD007754.pub2 21069697

[4] Darmstadt GL, Bhutta ZA, Cousens S, Adam T, Walker N, deBernis L. Neonatal Survival 2 Evidence-based, cost-effective interventions: how many newborn babies can we save? Lancet. 2005; 365: 977–88. 10.1016/S0140-6736(05)71088-6 15767001

[5] Campbell OMR, Graham WJ.; Lancet Maternal Survival Series steering group. Strategies for reducing maternal mortality: getting on with what works. Lancet. 2006; 368: 1284–99. 10.1016/S0140-6736(06)69381-1 17027735

[6] McConnell M, Ettenger A, Rothschild W, Muigai F, Cohen J. Can a community health worker administered postnatal checklist increase health-seeking behaviors and knowledge?: evidence from a randomized trial with a private maternity facility in Kiambu County, Kenya. BMC Pregnancy Childbirth. 2016; 16: 136 10.1186/s12884-016-0914-z 27260500 PMC4893209

[7] Braun R, Catalani C, Wimbush J, Israelski D. Community Health Workers and Mobile Technology: A Systematic Review of the Literature. PLoS ONE. 2013; 8: e65772 10.1371/journal.pone.0065772 23776544 PMC3680423

[8] Hackett K, Lafleur C, Nyella P, Ginsburg O, Lou W, Sellen D. Impact of smartphone-assisted prenatal home visits on women’s use of facility delivery: Results from a cluster-randomized trial in rural Tanzania. PLoS ONE. 2018; 13(6): e0199400 10.1371/journal.pone.0199400 29912954 PMC6005474

[9] Chen H, Chai Y, Dong L, Niu W, Zhang P. Effectiveness and Appropriateness of mHealth Interventions for Maternal and Child Health: Systematic Review. JMIR mHealth uHealth. 2018; 6(1): e7 10.2196/mhealth.8998 29317380 PMC5780618

[10] Amoakoh-Coleman M, Borgstein AB-J, Sondaal SF, Grobbee DE, Miltenberg AS, Verwijs M, et al. Effectiveness of mHealth Interventions Targeting Health Care Workers to Improve Pregnancy Outcomes in Low- and Middle-Income Countries: A Systematic Review. J Med Internet Res. 2016; 18(8): e226 10.2196/jmir.5533 27543152 PMC5010646

[11] Sondaal SFV, Browne JL, Amoakoh-Coleman M, Borgstein A, Miltenberg AS, Verwijs M, et al Assessing the Effect of mHealth Interventions in Improving Maternal and Neonatal Care in Low- and Middle-Income Countries: A Systematic Review. PLoS ONE. 2016; 11(5): e0154664 10.1371/journal.pone.0154664 27144393 PMC4856298

[12] Ministry of Health and Family Welfare. Indian Public Health Standards (IPHS) Guidelines for Primary Health Centres Revised. New Delhi: Government of India, 2012.

[13] Hogan MC, Foreman KJ, Naghavi M, Ahn SY, Wang M, Makela SM, et al Maternal mortality for 181 countries, 1980–2008: a systematic analysis of progress towards Millennium Development Goal 5. Lancet. 2010; 375: 1609–23. 10.1016/S0140-6736(10)60518-1 20382417

[14] Black R, Causens S, Jhonson H. Global, Regional and National Causes of Child Mortality in 2008. Lancet. 2010; 375: 1969–87. 20466419 10.1016/S0140-6736(10)60549-1

[15] International Institute for Population Sciences I. IIPS and Macro International. National Family Health Survey (NFHS-3), 2005–06: India: volume I. Mumbai, India [Internet; cited 2018 Nov 13]. http://rchiips.org/nfhs/nfhs3.shtml.

[16] Bajpai N, Dholakia RH. Improving the Performance of Accredited Social Health Activists in India. Work Papers Series Columbia Glob Centers 2011. South Asia, Columbia University, Mumbai, India.

[17] Maternal and Child Health Integrated Program (MCHIP). India’s Reproductive, Maternal, Newborn, Child, and Adolescent Health (RMNCH+A) Strategy [Internet]. USAID; 2014 [cited 2017 June 17]. https://www.mchip.net/sites/default/files/RMNCH+A%20in%20India.pdf.

[18] National Health Systems Resource Centre (NHSRC). ASHA Which Way Forward: Evaluation of ASHA program 2010–11 Report [Internet; cited 2019 Jan 23]. New Delhi: National Institute of Health and Family Welfare; 2011. http://www.nipccd-earchive.wcd.nic.in/sites/default/files/PDF/Evaluation_of_ASHA_Program_2010-11_Executive_Summary.pdf.

[19] Ministry of Tribal Affairs. Report of the high level committee on socioeconomic, health and educational status of tribal communities of India. New Delhi: Government of India; 2014.

[20] Ministry of Health and Family Welfare. District Level Household and Facility Survey -3 (DLHS-3). International Institute for Population Sciences I. Mumbai, India, 2014 [Internet; cited 2019 Jan 23]. http://rchiips.org/PRCH-3.html.

[21] National Health Systems Resource Centre (NHSRC). Evaluation theory and evaluation practice _ in the NRHM context. Berkeley, CA: Center for effective global action, University of California Berkeley; 2014 [cited 2019 Jan 23]. http://cega.berkeley.edu/assets/cega_events/38/Evaluations_of_the_National_Rural_Health_Mission.pdf.

[22] Modi D, Gopalan R, Shah S, Venkatraman S, Desai G, Desai S, et al Development and formative evaluation of an innovative mHealth intervention for improving coverage of community-based maternal, newborn and child health services in rural areas of India. Glob Health Action. 2015; 8: 26769 10.3402/gha.v8.26769 25697233 PMC4335194

[23] Modi D, Desai S, Dave K, Shah S, Desai G, Dholakia N, et al Cluster randomized trial of a mHealth intervention “ImTeCHO” to improve delivery of proven maternal, neonatal, and child care interventions through community-based Accredited Social Health Activists (ASHAs) by enhancing their motivation and strengthening. Trials. 2017; 18: 270. 28599674 10.1186/s13063-017-1998-0 PMC5466719

[24] National Health Systems Resource Centre (NHSRC). Update on ASHA program [Internet; cited 2019 Jan 23]. New Delhi, India: National Health Mission, Government of India; 2017 http://nhsrcindia.org/sites/default/files/UpdateonASHAProgramme-January-2017.pdf.

[25] National Rural Health Mission Ministry of Health & Family Welfare. Guidance note for implementation of RMNCH+A interventions in High Priority Districts. New Delhi: Government of India; 2013 [cited 2018 Aug 18]. http://www.venturecenter.co.in/collab/health/wp-content/uploads/2015/11/High-priority-districts.pdf.

[26] Government of India. List of High Priority Talukas in Gujarat–Community Action for Health. National Health Mission. New Delhi: Government of India; 2014 [cited 2018 Aug 18]. https://nrhmcommunityaction.org/list-of-high-priority-talukas-in-gujarat/.

[27] Government of India. Census of India 2011 [Internet; cited 2018 Sept 29]. http://censusindia.gov.in.

[28] Singh KS. People of India: Gujarat Part one Volume XXII. Mumbai: Popular Prakashan; 2003.

[29] Campbell M, Fitzpatrick R, Haines A, Kinmonth AL, Sandercock P, Spiegelhalter D, et al: Framework for the design and evaluation of complex interventions to improve health. British Medical Journal 2000, 321:694–696. 10.1136/bmj.321.7262.694 10987780 PMC1118564

[30] SEWA Rural. ImTeCHO Demonstration Video. SEWA Rural. Jhagadia [cited 2018 Oct 1]. https://www.youtube.com/watch?v=gdZ1vgC6gFo.

[31] International Institute for Population Sciences. District Level Household and Facility Survey (DLHS-4). Government of India. 2011. http://rchiips.org/pdf/DLHS4 Bid for field agency at 1IIPS,Mumbai..pdf. Accessed 18 August 2018.

[32] Shah P, Madhiwala N, Shah S, Desai G, Dave K, Dholakia N, et al. High Acceptability and Uptake of an Innovative Mobile-Phone Application among Community Health Workers in Rural Areas of India: An Implementation Research Study. National Medical Journal of India. Forthcoming. 10.4103/0970-258X.295956 32985439

[33] Källander K, Tibenderana JK, Akpogheneta OJ, Strachan DL, Hill Z, ten Asbroek AH, et al. Mobile Health (mHealth) Approaches and Lessons for Increased Performance and Retention of Community Health Workers in Low- and Middle-Income Countries: A Review. J Med Internet Res. 2013; 15: e17 10.2196/jmir.2130 23353680 PMC3636306

[34] Atnafu A, Otto K, Herbst CH. The role of mHealth intervention on maternal and child healthservice delivery: findings from a randomized controlled field trial in rural Ethiopia. mHealth. 2017; 3: 39–39. 10.21037/mhealth.2017.08.04 29184891 PMC5682387

[35] Borkum E, Sivasankaran A, Sridharan S, Rotz D, Sethi S, Manoranjini M, et al Evaluation of the Information and Communication Technology (ICT) Continuum of Care Services (CCS) Intervention in Bihar. Princeton: Mathemetica Policy Research, 2015.

[36] Lewin S, Munabi-Babigumira S, Glenton C, Daniels K, Bosch-Capblanch X, van Wyk BE, et al Lay health workers in primary and community health care for maternal and child health and the management of infectious diseases. Cochrane Database Syst Rev. 2010;(3): CD004015 10.1002/14651858.CD004015.pub3 20238326 PMC6485809

[37] Ciapponi A, Lewin S, Herrera CA, Opiyo N, Pantoja T, Paulsen E, et al Delivery arrangements for health systems in low-income countries: an overview of systematic reviews. Cochrane Database Syst Rev. 2017;9: CD011083 10.1002/14651858.CD011083.pub2 28901005 PMC5621087

[38] Lee SH, Nurmatov UB, Nwaru BI, Mukherjee M, Grant L, Pagliari C. Effectiveness of mHealth interventions for maternal, newborn and child health in low–and middle–income countries: Systematic review and meta–analysis. J Glob Health. 2016; 6: 010401 10.7189/jogh.06.010401 26649177 PMC4643860

[39] Lund S, Boas IM, Bedesa T, Fekede W, Nielsen HS, Sorensen BL. Association between the safe delivery app and quality of care and perinatal survival in Ethiopia: A Randomized Clinical Trial. JAMA Pediatr. 2016; 170(8): 765–771. 10.1001/jamapediatrics.2016.0687 27322089

[40] Bolan NE, Sthreshley L, Ngoy B, Ledy F, Ntayingi M, Makasy D et al mLearning in the Democratic Republic of the Congo: A mixed-methods feasibility and pilot cluster randomized trial using the safe delivery app. Glob Health Sci Pract. 2018;6(4):693–710. 10.9745/GHSP-D-18-00275 30591577 PMC6370362

[41] World Health Organization (WHO). WHO guideline recommendations on digital health interventions for health system strengthening. Geneva: WHO; 2019 [cited 2019 Jul 28]. https://www.who.int/reproductivehealth/publications/digital-interventions-health-system-strengthening/en/. 31162915

[42] Prime Minister’s Office. PM visits Vadnagar, launches Intensified Mission Indradhanush, addresses public meeting. Government of India. Bhubaneshwar. Press Information Bureau. 8 October, 2017 [cited 2018 Oct 2]. http://www.pib.gov.in/PressReleseDetail.aspx?PRID=1505368.

