# Supplementary material for: mHealth intervention “ImTeCHO” to improve delivery of maternal, neonatal, and child care services—A cluster-randomized trial in tribal areas of Gujarat, India
Source: PLoS Med. 2019 Oct 24;16(10):e1002939. doi: 10.1371/journal.pmed.1002939 (PMC6812744; doi:10.1371/journal.pmed.1002939)
Supplement: S1 Table — (DOCX) [file pmed.1002939.s001.docx]

|  | **Intervention** | **Control** | **Timing** |
| --- | --- | --- | --- |
| Refresher training (Three-days) of ASHAs for maternal, neonatal and child care along with one time provision of commodities | Yes | Yes | August, 2015 to January, 2016 |
| Four-day class room training by trainer from SEWA Rural and government followed by field mentoring of ASHAs for two weeks for use of ImTeCHO mobile phone application. Half a day classroom training of PHC staff, including medical officer for use of ImTeCHO web application | Yes | No |  |
| Use of ImTeCHO mobile and web based application by ASHAs, and PHC staff (Table 1 for detailed description) | Yes | No | February, 2016 to January, 2017 |
| Telephone care by a helpline counselor. Once an ASHA identified a complicated case, the counselor received notification on her ImTeCHO web interface. Subsequently, the counselor provided telephone care to families and ASHAs to manage complicated cases over phone. The support provided by the counselor involved confirmation of probable diagnosis made by ASHA, promotion of household level practices to care for complications, advice ASHAs for optimal management of complications, counsel families to seek care at higher referral facility if required, facilitate referral and alert PHC medical officer in case of emergencies. | Yes | No |  |
| Facilitation and technology support through mHealth facilitators (one per 50 ASHAs) who solved first level technology problems, ensured adherence to the ImTeCHO intervention by monitoring innovative process indicators using a facilitator dashboard available through ImTeCHO web, managed housekeeping functions in ImTeCHO web interface whenever ASHAs reported migration, death, and duplicate registration of a case, attended monthly PHC meetings, visited ASHAs occasionally to provide ongoing training/supervision/motivation to those ASHAs whose performance parameters within ImTeCHO was consistently poor, and sent SMS to ANMs and medical officer to inform about high risk cases diagnosed by ASHAs. | Yes | No |  |
| Implementation of ASHA program as per routine | Yes | Yes |  |
| Token, additional incentive to ASHAs (ranging from US $ 4 to 11 monthly per ASHA) for using ImTeCHO application | Yes | No |  |
| Change management activities aimed at improving acceptability of the intervention among participants | Yes | No |  |
| Software maintenance | Yes | No |  |
