# Supplementary material for: mHealth intervention “ImTeCHO” to improve delivery of maternal, neonatal, and child care services—A cluster-randomized trial in tribal areas of Gujarat, India
Source: PLoS Med. 2019 Oct 24;16(10):e1002939. doi: 10.1371/journal.pmed.1002939 (PMC6812744; doi:10.1371/journal.pmed.1002939)
Supplement: S1 Text — (DOC) [file pmed.1002939.s003.doc]

**IMPLEMENTATION RESEARCH PROPOSAL**

**Cluster Randomized Trial of a mHealth Intervention “*ImTeCHO*” to Improve Delivery of Proven Maternal, Newborn and Child Care Interventions through Community Based Accredited Social Health Activists (ASHAs) by Enhancing Their Motivation and Strengthening Supervision in Tribal Areas of Gujarat, India**

**IMPLEMENTATION RESEARCH PROPOSAL**

**TITLE OF THE STUDY**:

Cluster Randomized Trial of a mHealth Intervention “ImTeCHO” to Improve Delivery of Proven Maternal, Newborn and Child Care Interventions through Community Based Accredited Social Health Activists (ASHAs) by Enhancing Their Motivation and Strengthening Supervision in Tribal Areas of Gujarat, India

**ORGANIZATION RESPONSIBLE FOR THIS STUDY:**

SEWA Rural (Society for Education, Welfare and Action, Rural), Jhagadia, Dist: Bharuch, Gujarat. 393110. Phone number: 02645-220021. Fax: 02645-220313. Email: sewarural@ymail.com

**PARTNER ORGANIZATIONS:**

1. Health and Family Welfare Department, Government of Gujarat.
2. Argusoft India Ltd, Gandhinagar, Gujarat.
3. ICMR and WHO

**PRINCIPAL INVSTIGATOR:**

Pankaj Shah, MD, Director, Community Health, SEWA Rural, Jhagadia, Dist: Bharuch, Gujarat, 393110. Phone number: 02645-220021, 09426120316. Fax: 02645-220313. Email: pankaj8892@yahoo.co.in, sewarural@ymail.com

**CO- INVESTIGATORS**:

(1) Dhiren Modi, MD, Community Health Physician, SEWA Rural.

(2) Gayatri Desai, MD, Head, Obstetrics and Gynecology Department, SEWA Rural hospital

(3) Ravi Gopalan, CEO, Argusoft India Ltd

(4) Shrey Desai, MD, MPH, Research coordinator, SEWA Rural

(5) Shobha Shah, PGDPHM, Training coordinator, SEWA Rural

(6) Kapil Dave, BHMS, PGDHHM, Research associate, SEWA Rural

(6) Nishith Dholakia, MD, Additional Director, Health and Family Welfare Department, Govt of Gujarat

**DURATION OF STUDY:**

1. Period for collecting baseline data, implementing intervention and collecting endline data and analyzing data: 36 months

**Primary funder:**

The Indian Council of Medical Research (ICMR)

P.O. Box No. 4911
Ansari Nagar
New Delhi - 110029
India

***“The difference between what we do and what we are capable of doing would suffice to solve most...problems.”***

Mahatma Gandhi

Table of content

1. Summary [9](#__RefHeading___Toc418709075)

2. Context and Back ground: [10](#__RefHeading___Toc418709076)

2.1 Suboptimal delivery of proven interventions and inequitable utilization of health services/outcome [10](#__RefHeading___Toc418709077)

2.2 ASHA program [11](#__RefHeading___Toc418709078)

2.3 Online Mother and Child Tracking System (MCTS) [12](#__RefHeading___Toc418709079)

3. Problem statement: [12](#__RefHeading___Toc418709080)

4. Rationale for intervention [19](#__RefHeading___Toc418709081)

5. Objectives [22](#__RefHeading___Toc418709082)

5.1 Primary objectives and research questions [22](#__RefHeading___Toc418709083)

5.2 Secondary objectives and research questions [25](#__RefHeading___Toc418709084)

5.3 Summary of participants, intervention, comparator and outcomes (PICO) [28](#__RefHeading___Toc418709085)

6. Study design [29](#__RefHeading___Toc418709086)

6.1 Design [29](#__RefHeading___Toc418709087)

6.2 Rationale for design [30](#__RefHeading___Toc418709088)

6.3 Inclusion and exclusion criteria [31](#__RefHeading___Toc418709089)

7. Study setting [31](#__RefHeading___Toc418709090)

8. Sample size calculation [33](#__RefHeading___Toc418709091)

9. Study participants [35](#__RefHeading___Toc418709092)

9.1 ASHA [35](#__RefHeading___Toc418709093)

9.2 ASHA facilitator (AF) [35](#__RefHeading___Toc418709094)

9.3 Auxiliary Nurse Midwife(ANM) [35](#__RefHeading___Toc418709095)

9.4 Medical officer [35](#__RefHeading___Toc418709096)

9.5 mHealth facilitator (mHF) [35](#__RefHeading___Toc418709097)

9.6 PHC support staff [36](#__RefHeading___Toc418709098)

9.7 SEWA Rural helpline/ telephone care [36](#__RefHeading___Toc418709099)

10. Description of intervention and how it contributes to reducing mortality and morbidity through improving delivery of MNCH interventions [36](#__RefHeading___Toc418709100)

10.1 Conceptual framework [36](#__RefHeading___Toc418709101)

10.1.1 Mobile phone as job aid to increase coverage of MNCH care [36](#__RefHeading___Toc418709102)

10.1.2 Mobile phone as job-aid to facilitate care to complicated cases [37](#__RefHeading___Toc418709103)

10.2 Intervention: [39](#__RefHeading___Toc418709104)

10.1.1 Mobile phone as job aid to ASHAs to increase coverage of maternal, newborn and child care [40](#__RefHeading___Toc418709105)

10.1.2 Mobile phone as job aid to ASHAs and ANMs to facilitate care of cases with complications [41](#__RefHeading___Toc418709106)

10.1.3 Web interface as job aid to medical officer and PHC staff for monitoring and supporting program [42](#__RefHeading___Toc418709107)

10.3 Phases of intervention [43](#__RefHeading___Toc418709108)

11 Control arm (Comparator) [45](#__RefHeading___Toc418709109)

12 Outcomes and their measurement [45](#__RefHeading___Toc418709110)

13 Project plan and management [47](#__RefHeading___Toc418709111)

13.1 Implementation of intervention [47](#__RefHeading___Toc418709112)

13.1.1 Pre-intervention baseline household survey [47](#__RefHeading___Toc418709113)

13.1.2 Training and intervention maturation [47](#__RefHeading___Toc418709114)

13.1.4 Implementation of ImTeCHO intervention and field organization [48](#__RefHeading___Toc418709115)

13.2 Timeline [50](#__RefHeading___Toc418709116)

13.3 Output of the project and deliverables [50](#__RefHeading___Toc418709117)

15. Data management and statistical analysis [51](#__RefHeading___Toc418709118)

15.1 Defining data needs and designing data collection tool [51](#__RefHeading___Toc418709119)

15.2 Data flow process [51](#__RefHeading___Toc418709120)

15.3 Data Safety and Monitoring Board [52](#__RefHeading___Toc418709121)

15.4 Analysis plan [52](#__RefHeading___Toc418709122)

16. Quality assurance [53](#__RefHeading___Toc418709123)

16.1 For home surveys [53](#__RefHeading___Toc418709124)

16.2 Service to be provided by ASHAs, ANMs and medical officer [53](#__RefHeading___Toc418709125)

16.3 Mobile application and server [53](#__RefHeading___Toc418709126)

16.4 General study implementation [54](#__RefHeading___Toc418709127)

17. Ethical considerations [56](#__RefHeading___Toc418709128)

17.1 Areas of risk and potential solution [56](#__RefHeading___Toc418709129)

17.2 Areas of benefit [57](#__RefHeading___Toc418709130)

17.3 Ethical review [57](#__RefHeading___Toc418709131)

18. Outcomes and policy implications of study: [57](#__RefHeading___Toc418709132)

18.1 Uniqueness of ImTeCHO compared to other models [57](#__RefHeading___Toc418709133)

18.2 Implications on policy [58](#__RefHeading___Toc418709134)

18.3 Plan for dissemination [58](#__RefHeading___Toc418709135)

19. Planning for un-expected: [59](#__RefHeading___Toc418709136)

20. Strengths of research team and collaborations [59](#__RefHeading___Toc418709137)

20.1 About investigators [59](#__RefHeading___Toc418709138)

20.2 Roles and responsibilities of all investigators [60](#__RefHeading___Toc418709139)

20.3 About organizations involved in the study [60](#__RefHeading___Toc418709140)

20.3.1 SEWA Rural [60](#__RefHeading___Toc418709141)

20.3.2 Argusoft India Ltd. [61](#__RefHeading___Toc418709142)

20.3.3 Department of Health and Family Welfare, Government of Gujarat [61](#__RefHeading___Toc418709143)

21. Feasibility of completing the study in time [61](#__RefHeading___Toc418709144)

22. Abbreviations [63](#__RefHeading___Toc418709145)

23 Appendices [64](#__RefHeading___Toc418709146)

# 1. Summary

To facilitate delivery of proven maternal and newborn interventions, a new cadre of village-based Community Health Workers, called Accredited Social Health Activist (ASHA), was created in 2005 under the aegis of the National Rural Health Mission in India. Evaluations have noted that coverage of selected maternal, newborn and child health (MNCH) services, to be delivered by ASHAs is low. Reasons for low coverage are inadequate supervision and support to ASHAs apart from insufficient skills, poor quality of training, and complexity of tasks to be performed.

Proposed study aims to implement, and evaluate an innovative intervention based on mobile-phone technology to improve performance of ASHAs through better supervision and support in predominantly tribal and rural communities of Gujarat, India. The intervention which is a newly-built mobile-phone application will be used and evaluated in three ways: (1) Mobile-phone as a job-aid to ASHAs to increase coverage of maternal and newborn care services (2) Mobile-phone as a job-aid to ASHAs and Auxiliary Nurse Midwives (ANMs) to increase coverage of care among maternal and newborn cases with complications by facilitating referral, if indicated and home-based-care (3) Web-interface as job-aid for medical-officers to improve supervision and support to ASHA program.

This will be a two-arm cluster randomized trial of 36 months duration. There will be eleven Primary Health Centers (with a population of approximately 20,000 populations each) in each arm. Primary outcome measures include coverage of selected MNCH services and care received by complicated cases. Outcomes will be measured by conducting household surveys at baseline, and post-intervention which will be compared with usual practice in control area where current level of services provided by the government will continue.

Primary analysis will be done by “intention to treat”. For each of the primary and secondary outcome, effect size (95% confidence interval), after adjustment if required, would be computed.

# 2. Context and Back ground:

## 2.1 Suboptimal delivery of proven interventions and inequitable utilization of health services/outcome

*The Lancet* produced series of articles in 2003 and 2005 to guide efforts to reduce maternal and child mortality. One of the most important conclusions of the series was “*Although research on interventions is plentiful, little is known about the characteristics of delivery strategies capable of achieving and maintaining high coverage for specific interventions in various epidemiological, health system, and cultural contexts*.” [[1]](#footnote-2). Data from the District Level Household and Facility Survey-3 (DLHFS-3) supports above observation which showed that coverage of complete antenatal check up, institutional delivery and complete immunization in India was only 18%, 47% and 54% respectively in 2008. For Gujarat, coverage of complete antenatal check up, institutional delivery and complete immunization was 20%, 56%, and 55% respectively in 2008. There are many reasons to explain suboptimal delivery of proven interventions such as inadequate governance, finances, monitoring, human resources, information and infrastructure[[2]](#footnote-3).

The coverage of maternal and child health (MCH) interventions and outcomes are also inequitably distributed among different Indian states and among various ethnicities. The tribal community has worse health indicators compared to Non tribal as seen in Table 1.

**Table 1 Comparison of selected health and coverage indicators for tribal community and India**

| **Health indicator** | **India ( over all)** | **India-tribal** |
| --- | --- | --- |
| **Under 5 Mortality Rate** | 59 | 96 |
| **Infant Mortality Rate** | 49 | 62 |
| **No ANC visit** | 27.9% | 43.1% |
| **Institutional delivery rate** | 41% | 19% |
| **No Tetanus toxoid injection taken** | 19.2% | 38.7% |

Source: NHFS-2 (1998-99) and NHFS-3(2005-06)

Such disparity has been observed among project villages of SEWA Rural too (Full description of SEWA Rural to follow in Section 20.2). SEWA Rural implemented a safe motherhood and newborn survival project from April-2003 to March-2011 among 168 villages of Jhagadia block, Gujarat consisting of total population of 171,000. Though maternal, newborn and child health outcomes improved significantly overall, there was a large disparity between villages with mostly tribal population compared to villages with mixed population[[3]](#footnote-4). Table 2 compares health indicators among these villages.

**Table 2 Maternal and neonatal health indicators and health services coverage in Jhagadia block**

| **Indicator** | **23 villages (mostly tribal)** | **145 villages (mixed population)** |
| --- | --- | --- |
| **Maternal Mortality Ratio (MMR)** | 374 | 116 |
| **Newborn Mortality Rate (NMR)** | 47 | 26 |
| **Institutional delivery rate** | 44% | 69% |

Source: SEWA Rural’s in-service data (April 2011)

## 2.2 ASHA program

To overcome above challenge, a new cadre of village based Community Health Workers (CHWs), called Accredited Social Health Activist (ASHA), was created under the aegis of the National Rural Health Mission (NHRM)[[4]](#footnote-5). An ASHA is native of a particular village and is selected by the gram sabha with help of local government health staff. ASHAs are expected to be literate and have educational qualification of at least 8^th^ grade. ASHAs are expected to contribute few hours (3-5 hours) every day for her activities. She is expected to receive performance based incentive from the PHC[[5]](#footnote-6).

Many of the community based MNCH interventions are expected to be implemented or facilitated by ASHA during her home visits. As per ASHA training module 6 and 7, the maternal care services that she is expected to deliver include counselling pregnant women, ensuring complete antenatal care through home visits and enabling care at monthly Village Health and Nutrition Days (VHND), assisting households to make birth plan, identification and referral of complicated cases along with supporting households for safe delivery. Regarding newborn care, ASHAs are expected to undertake at least 6 post-partum visits, (5 in case of hospital delivery), counsel and problem solve breastfeeding problems, keep the baby warm, identify and perform basic management of Low Birth Weight (LBW) and pre-term babies, conduct examinations needed for identification/first contact care for sepsis and asphyxia [[6]](#footnote-7). ASHAs are expected to weigh and identify high-risk babies (low birth weight and/or premature) and use protocols to manage such babies. Also, ASHAs are expected to identify babies with sepsis, provide first level care and refer them to a health facility. ASHAs are expected to treat complicated child case of ARI/pneumonia and diarrhoea, counsel families for preventing malnutrition, and ensure complete immunizationError: Reference source not found.If family is unable to go to referral facility then Auxilliary Nurse Midwife (ANM) should make home visit on priority basis[[7]](#footnote-8). ASHA drug kit should include Oral Rehydration Solution (ORS), paracetamol, oral contraceptive pills, condoms, Iron-Folic Acid (IFA), cotrimoxazole, chloroquine, dicyclomine, albendazole, nischay kit (pregnancy detection kit), thermometer, bandages, cotton Swab, betadine and gentian VioletError: Reference source not found,Error: Reference source not found.

## 2.3 Online Mother and Child Tracking System (MCTS)

The Government of Gujarat has initiated online mother and child tracking system to monitor coverage of MCH interventions and outcomes. Medical officers at every level of health system are expected to use online mother and child tracking system (MCTS) to monitor programs. Data is recorded by the ANMs on E-Mamta card with help of ASHAs which is passed on to data entry operator who is located at PHC level. The collected data mainly pertains to health outcomes and services to be provided by the ANMs. A computer with internet connectivity is placed at every PHC. Data entry operator enters data submitted by ANM into the MCTS. The software system checks services provided against service that every pregnant woman and child is expected to receive. The system prepares a list of pending services automatically. Also, reports are generated by the system which helps medical officers at every level to monitor program [[8]](#footnote-9), [[9]](#footnote-10).

The Government of Gujarat was recognized for outstanding performance in citizen-centric service delivery by the Government of India in 2012 for implementing MCTS[[10]](#footnote-11). The MCTS has now been scaled up nationally.

# 3. Problem statement:

*3.1 Low coverage of MNCH interventions to be provided or facilitated by ASHAs.*

There have been 2 large evaluations of ASHA program since its initiationError: Reference source not found,[[11]](#footnote-12). These evaluations noted that coverage of selected MNCH interventions to be implemented and facilitated by ASHA is highly variable as briefly summarized in Table 3 and 4. Data from best and worst performing states are in bold letters in Table 3 and 4.

**Table 3 Coverage of selected maternal care services to be implemented or facilitated by ASHAError: Reference source not found**

| **Indicators** | **% of service users A who received three ANCs or more** | **% of service user A who got JSY money and whom ASHA helped for JSY payment** | **% of Service User A who went for institutional delivery and cited ASHA as a motivator** | **% of service users A who had an ANC card made** | **% of service users who were counseled on post partum care** | **ASHAs who knew about Foul smelling discharge as danger sign to look for.** | **% of pregnant women line listed who were escorted to the facility** | **% of service user A** |
| --- | --- | --- | --- | --- | --- | --- | --- | --- |
|  |  |  |  |  |  |  |  | **who had institutional delivery and whom ASHAs helped in arranging transport** |
| **National average** | **58.1** | **53.8** | **68** | **51.2** | **34** | **26.7** | **31.7** | **41** |
| **Kerala** | **89.4** | **29** | **49.9** | 53.1 | 56.8 | 25 | **7.7** | 14.3 |
| **Orissa** | 70.4 | 50.1 | **94.3** | 52 | **61.6** | **36.5** | **58.7** | **77.9** |
| **West Bengal** | 48.8 | 71.3 | 61.3 | **68.9** | 55 | 25.5 | 2.1 | **10.8** |
| **Assam** | 54 | 72.5 | 91.4 | 63.8 | 26.9 | **17.5** | 40.7 | 46.4 |
| **Rajasthan** | 52.2 | 23.7 | 55.8 | 42.7 | **9.2** | 26 | 43.7 | 32.2 |
| **Andra Pradesh** | 82.1 | **80.8** | 56.1 | 45.5 | 26.7 | 32.5 | 25 | 55.5 |
| **Bihar** | **20.8** | 41.8 | 74.6 | **31.6** | 13.3 | 26 | 54.2 | 45.1 |
| **Jharkhand** | 50.7 | 65.9 | 64.7 | 56.8 | 32.2 | 27.4 | 25.5 | 50.9 |

*Service A Users: Those who used ASHA’s services during pregnancy, child birth or in the newborn period.

**Table 4 Coverage of selected newborn care services to be provided by ASHAError: Reference source not found**

| **Indicators** | ***% of user A who were visited by ASHA on day of birth*** | **% of User A *who were visited more than two times in first month after delivery*** | ***% of User A where ASHA was present during weighing of the new born*** | ***% of user A reporting that ASHA was present and helped in early breast feeding*** | ***% of user A who reported that the newborn was sick in 1^st^ month and sought advice from the ASHA in seeking care for the sick newborn*** | ***% of user A who reported that the newborn was sick in 1^st^ month and reporting that ASHA helped in identifying danger signs of the new born*** |
| --- | --- | --- | --- | --- | --- | --- |
|
| **National average** | 34 | 43.2 | 62.7 | 28 | 52.3 | 39 |
| **Kerala** | **11** | 43.3 | **20.8** | 12 | 56.4 | 41.3 |
| **Orissa** | **59.9** | **57.1** | **93.4** | **63.7** | 58.9 | 43.4 |
| **West Bengal** | 12.4 | 36.6 | 49.5 | **5.8** | 41.5 | 29 |
| **Assam** | 41.1 | 48.8 | 55.2 | 17.8 | 49.5 | **26.1** |
| **Rajasthan** | 16.5 | 37.9 | 71.6 | 26.2 | 42 | 38.6 |
| **Andra Pradesh** | 46.1 | **57.1** | 66.9 | 39.3 | **78.7** | **56.2** |
| **Bihar** | 47 | **31.6** | 70.4 | 31.2 | **39.3** | 36.6 |
| **Jharkhand** | 40.9 | 37.6 | 78.3 | 31 | 56.3 | 47.5 |

*Service A Users: Those who used ASHA’s services during pregnancy, child birth or in the newborn period.

**Coverage of selected child care services to be provided by ASHAError: Reference source not found**

| Indicators | user Bs who had diarrhea the % to whom ASHA gave ORS from her kit | Of those User Bs with signs of ARI, the % in Whom ASHA helped in some way | % of User B who started complementary feeding at 6 months | % of service user B – child immunized for measles |
| --- | --- | --- | --- | --- |
|
| **National average** | 57 | 77 | 48 | 59 |
| **Kerala** | **82** | 93 | **72** | 82 |
| **Orissa** | **81** | **96** | **49** | **64** |
| **West Bengal** | 51 | 75 | 31 | **67** |
| **Assam** | 54 | 64 | 38 | 62 |
| **Rajasthan** | 56 | 64 | 38 | 49 |
| **Andra Pradesh** | 71 | **96** | 71 | 32 |
| **Bihar** | 26 | **67** | 40 | 53 |
| **Jharkhand** | 36 | 67 | 49 | 63 |

**Service B Users: Those who used ASHA’s services for their child.

Coverage of selected maternal, newborn and child services to be facilitated by ASHAs is low in Gujarat too as indicated in Table 5 [[12]](#footnote-13). Another study showed that only 17% neonates were visited by ASHA within 24 hours and 40% of neonates were visited within one week of births in rural areas respectively[[13]](#footnote-14).

**Table 5 Coverage of selected maternal, newborn and child interventions in Gujarat (DLHFS-3, 2007-08)**

| **Indicators** | **Total (%)** | **Rural (%)** | **Urban (%)** |
| --- | --- | --- | --- |
| Mothers who received any antenatal check-up | 71.5 | 66.4 | 88.4 |
| Mothers who had antenatal check-up in first trimester | 52.4 | 45.9 | 73.4 |
| Mothers who had three or more ANC | 56.8 | 50 | 79 |
| Mothers who had at least one tetanus toxoid injection | 68.6 | 63.1 | 86.4 |
| Mothers whose Blood Pressure (BP) taken | 51 | 44.8 | 71.3 |
| Mothers who consumed 100 IFA Tablets | 50.7 | 52.3 | 45.4 |
| Mothers who had full antenatal check-up | 19.9 | 16 | 32.8 |
| Mothers who received post-natal care within two weeks of delivery | 59.5 | 52.9 | 81.1 |
| Children under 3 years breastfed within one hour of birth | 48.3 | 47.4 | 51.6 |

Asd

**Table 6 Coverage of selected MNCH interventions in selected villages of Jhagadia block during 2014****[[14]](#footnote-15)**

| **Indicators** | **Prevalence (%)** |
| --- | --- |
| **ANC** |  |
| First antenatal examination during first trimester | 57% |
| Full antenatal examination | 19% |
| At least 4 ANC examination by ANM/doctor | 79% |
| At least 3 ANC home visit by ASHA | 65% |
| **DELIVERY** |  |
| Early initiation of breast feeding | 73% |
| **POSTPARTUM** |  |
| ASHA visited at home within 24 hours of delivery (in case of home delivery) or within 24 hours of return to home from hospital in case of hospital delivery | 32% |
| At least 5 home visit by ASHA within first month of delivery AND 2 home visit within first week (irrespective of place of delivery) | 2% |
| Mothers who received satisfactory education/counseling about caring for newborn baby from ASHA during her home visits after last delivery | 2% |
| **YOUNG CHILDREN (6-9 MONTHS)** |  |
| Exclusive breast feeding | 23% |
| Child was fed solid, semisolid or soft food at least once within last 24 hours | 74% |
| Mother knew status of child on WHO growth chart | 6% |
| **CARE SEEKING** |  |
| Sought help from ASHA for ANC complication | 54% |
| Sought help from ASHA for postnatal maternal complication | 9% |
| Sought help from ASHA for newborn complication | 27% |
| Received ORS | 43% |
| Child received ORS and ORS was supplied by ASHA | 6% |
| Sought help from ASHA for pneumonia/fever | 23% |

*3.2 Low care seeking from appropriate health personnel*

Large number of neonates and pregnant women with complications do not seek care at health facilityError: Reference source not found,[[15]](#footnote-16),[[16]](#footnote-17),[[17]](#footnote-18) and those who are unable to go to health facility do not receive any care at homeError: Reference source not found. Only 25% of neonates and 55% of pregnant women with complications sought treatment from a health facility in Jhagadia block, Gujarat (in-service data, SEWA Rural). 61% of women sought care for any complications during antenatal period whereas only 59% of women sought care for post-delivery complications in GujaratError: Reference source not found. Only 41% of newborns with complications got treatment from a health facility in study conducted in Wardha, India[[18]](#footnote-19). Poor care seeking could be due to delay in recognition of complications at community level because of lack of knowledge about danger signs and presence various barriers for seeking careError: Reference source not found^,Error: Reference source not found^,[[19]](#footnote-20),[[20]](#footnote-21). Only a quarter of households in Gujarat had knowledge about danger signs among newbornsError: Reference source not found. 46% of newborns with complications did not get any care in a study conducted at WardhaError: Reference source not found. Large numbers of complicated cases who stay home do not receive any care at home due to non-availability of doctors or other qualified health personnel at homeError: Reference source not found. Though the ANM is expected to attend to complicated cases at home in case the family is unable to go to health facility, there is no system in place where ANMs and medical officers could be notified immediately about such complicated cases at home. This limits their capacity to track and attend to such cases. ASHAs, who are trained to identify and manage selected minor newborn and maternal complications, find it difficult to remember complex algorithms and management guidelinesError: Reference source not found which limits their capacity to appropriately manage complicated cases at home.

3.3 *Inadequate information and institutional capacity for monitoring and supporting maternal, newborn and child care services to be provided through ASHA.*

There are some important challenges which limit MCTS’s and ASHA program’s potentially immense usefulness. Three of the most important challenges are: (1) delay in data entry, (2) Lack of clear understanding about how ASHAs’ performance will be recorded, tracked and reviewed (3) inadequate information to track complicated cases and (4) inadequate institutional capacity for ASHA’s performance monitoring, replenishment of supplies and timely payment of incentives[[21]](#footnote-22).

As of July 2011, only 50 lacs women were registered against expected 2.5 crores nationally[[22]](#footnote-23). According to the National Informatics Center (NIC), registration of pregnant women using MCTS ranged from 96% in Tamil Nadu to 0% in Mizoram. Only seven states were able to register more than 75% of all pregnant women using MCTS. For children, only four states registered more than 75% children using MCTS[[23]](#footnote-24). Verification and quality of data entered in MCTS are also important challengesError: Reference source not found^,Error: Reference source not found^. Additionally, though there is a system to send reminders for pending services, the system to track complicated maternal and child cases is inadequate.

There is a lack of regular and reliable supervisionError: Reference source not found. In a survey, 87% of ASHAs reported that there was lot of delay in payment of incentives and 25% of ASHAs felts that they are not incentivized enough Error: Reference source not found,[[24]](#footnote-25). Replenishment of supplies is often erraticError: Reference source not found,Error: Reference source not found. Reasons for above findings are:

1. Lack of clear understanding about how ASHAs’ performance will be recorded, tracked and reviewedError: Reference source not found.
2. Manual process of managing supplies and calculating and disbursing performance-based-incentives to ASHAs is difficultError: Reference source not found,Error: Reference source not found,Error: Reference source not found.
3. Inadequate information to track complicated cases.

# 4. Rationale for intervention

**Table 6: Rational for intervention****[[25]](#footnote-26)**

| **No** | **Reasons for problem** | **Solutions through ImTeCHO** |
| --- | --- | --- |
| *A. Low coverage of MNCH interventions to be provided or facilitated by ASHAs.* | | |
| 1 | Poor skills of ASHAs ( Do not remember the algorithm, triage system , poor counseling skills, inadequate training)Error: Reference source not found | Use of checklist, images, videos (for common counseling subjects), algorithms, automated risk stratification. Distance learning through mobile phone. |
| 2 | Inadequate understanding about ASHA’s roles and responsibilities Error: Reference source not found | Automated scheduling and reminder alerts based on prescribed roles and responsibilities |
| 3 | Poor motivation due to inadequate incentives considering work done by ASHAs Error: Reference source not found^,Error: Reference source not found^ | Records of services provided by ASHA will be automatically stored, which can be used to calculate incentive she deserves on time. A message will be displayed on screen stating incentive earned after ASHA provides every service. |
| 4 | Irregular suppliesError: Reference source not found | Automated supply management (as described below) |
| 5 | Barriers to behavior change at household level | Checklist to assess barriers and reminder to address those barriers |
| *B. Low care seeking from appropriate health personnel* | | |
| 6 | Difficult for ASHA to identify and triage complicated cases Error: Reference source not found | Mobile phone equipped with algorithms would provide diagnosis and risk stratification based on information entered by ASHA |
| 8 | Presence of various barriers (such as lack of knowledge about seriousness of morbidity, advanced planning to deal with complications etc) reduces the chances of referral of complicated cases to health facilityError: Reference source not found,Error: Reference source not found,Error: Reference source not found | Counseling videos about danger signs will be available in mobile phone to increase knowledge about complications  Use of checklist to encourage households to plan for complications (complication readiness) |
| 7 | Difficult for ASHA to manage selected complicated cases at home (who refuse to get referred), especially complicated newborn cases due to complexity of algorithms Error: Reference source not found | Mobile phone equipped with algorithms (based on ASHA modules) would provide management guidelines to ASHA for selected newborn and maternal complications |
| 7 | ANM and medical officer do not know in real time about mothers and newborns with complications | Automated alert would go to an ANM and medical officer instantly if complicated case is identified by ASHA during home visit; thus, ANM can plan to visit such cases in near future |
| *C. Inadequate information and institutional capacity for monitoring and supporting maternal, newborn and child care services to be provided through ASHA* | | |
| 9 | Information about maternal, newborn and child care services to be provided by ASHA is inadequate in MCTS Error: Reference source not found | This data will be available on proposed web interface. This web interface will be designed in such a way that it could be integrated in MCTS system ultimately. |
| 10 | Do not get timely information in MCTSError: Reference source not found^,Error: Reference source not found^ | Most of the information will be entered by ASHA using mobiles so that it will be available to medical officer on real time basis which would help him/her to supervise program. |
| 11 | Verification and quality of data is a challenge Error: Reference source not found^,Error: Reference source not found^ | Time-stamp and information about duration of visit along with photo of beneficiary will be available for every home visits made by ASHA and ANM. Additionally, features such as availability of GPS tools on mobiles can give information about position of ASHA and ANM. Such features will help medical officer to assess truthfulness of data. |
| 12 | Effort intensive process of managing supplies and calculating and disbursing incentives to ASHAError: Reference source not found^,Error: Reference source not found^ | System will send supply alerts to ASHAs and send information to PHC staff to re-supply stocks when necessary. Electronic record of ASHA’s performance along with automated calculation of incentives would reduce efforts required at PHC staff level. |
| 13 | Lack of real time information about complicated cases | List of complicated cases will be available on web interface as soon as complicated case is diagnosed by ASHA or ANM |

* mHealth will not be able to modify some of the reasons for poor coverage of care. These reasons include poor selection of ASHAs, poor training of ASHAs, weak community based monitoring, occupation with other health program/household duties, lack of money and access to hospital.

Table 6 states possible reasons for problem identified in Section 5 and its potential mHealth solutions. Most of the reasons pertain to complexity of providing evidence based solutions; complexity is related to series of simple inputs required at multiple level including quality of training, skills of ASHA, accountability, monitoring structure, payment of incentives and managing supplies. One of the solutions to manage such complexity is “*checklist*”[[26]](#footnote-27). Proposed intervention will integrate checklist (to insure standardization of services) with other features that mobile technology offers such as ability to transfer data instantly and apply algorithm automatically to data entered along with features to ensure check-and-balance for truthfulness and accuracy of collected information. Other features of mobile telephony such as capability of displaying multimedia and transferring information instantly via GPRS will be used.

Globally, many experiments are underway to improve maternal, newborn and child care using mobile phones in variety of ways. The Community Health Workers (CHWs) are using digital forms and protocols deployed in mobile phones in a pilot project in Tanzania for collecting and compiling data related to maternal health though the authors have noted need for assessment of impact on health outcomes[[27]](#footnote-28). Health workers in Rwanda are text messaging tools for faster communication with base hospital and early assessment shows positive impact on health outcomes[[28]](#footnote-29). A pilot project in Ghana is using Short Messaging System (SMS) based model where pregnant women would receive regular health education messages according to pregnancy period[[29]](#footnote-30). CommCare, mHealth platform developed by Dimagi Inc., uses multimedia in mobile phones to promote healthy behaviors[[30]](#footnote-31). Personal Digital Assistant (PDA) was used to implement Integrated Management of Childhood Illnesses (IMCI) protocol in Tanzania[[31]](#footnote-32).

There is evidence for effectiveness of using mHealth solutions to increase adherence to Anti-retroviral treatment, suppression of viral load, and case management of malaria by CHWs[[32]](#footnote-33),[[33]](#footnote-34)*.* Unfortunately, such strong evidence is not available in area of maternal health; this lack of evidence has prevented it to be used at scale[[34]](#footnote-35).

# 5. Objectives

## 5.1 Primary objectives and research questions

Primary objective 1: Examine effect of mHealth solutions in the form of job-aid to ASHAs during her scheduled home visit to increase coverage of selected MNCH interventions to be provided by her in tribal and rural areas of Gujarat.

Research question 1: Can mHealth solutions in the form of job-aid to ASHAs during her scheduled home visit increase coverage of following indicators compared with usual care among tribal areas of Gujarat?

There will be two primary outcomes of interest.

The first being proportion of neonates/mothers who receive at least two postnatal home visits within first week of delivery by ASHA. As most of the neonatal deaths occur during first few days of life, it is recommended that neonates should be visited three times during first week including day of delivery, third day and preferably seventh day[[35]](#footnote-36). However, most (78% according to coverage evaluation survey of UNICEF in 2009) of the deliveries in Gujarat now occur in facility where ASHAs’ role ends up being limited in presence of the facility based more qualified health workers[[36]](#footnote-37). Also, ASHAs’ visit on the day of delivery is influenced by variety of factors with limited role of the ImTeCHO intervention. Hence, we decided to focus on coverage of ASHAs’ two visits during first week after the mother/neonates returns home after discharge from the facility.

- Improve proportion of neonates/mothers who received at least two postnatal home visits within first week of delivery by ASHA from 46% to 66%.

The second primary outcome of interest will be a composite coverage index will be calculated using following formula and rationale.

Modified ASHA-centric composite coverage index (MACCI) = 0·25 × (0.33 × [Complete ASHA home visit during antenatal period +Full ANCS+SBA] + [Complete HBNC] + 0·5 × [DPT3 + EBF] + 0·33 × [Care seeking for newborn complications+ORT + ARI/febrile illness])

In which,

1. Maternal care domain

- Complete ASHA home visit during antenatal period = proportion of mothers who were visited at home by ASHA at least three times during last pregnancy including at least one visit during last trimester,
- Full ANCS = proportion of mothers with full antenatal examination (at least three antenatal examination, one Inj.TT and 100 IFA tablets)Error: Reference source not found
- SBA = proportion of mothers who delivered in a facility as most of the deliveries attended by skilled attendant are those taking place at a facility in GujaratError: Reference source not found

1. Newborn care domain

- Complete HBNC = proportion of neonates/mothers who received the recommended number of postnatal visits and at recommended times within first month of delivery by ASHAError: Reference source not found

1. Young infant care domain

- DPT3 = proportion of infants (6-8 months) who received three doses of diphtheria, pertussis, and tetanus vaccine or three doses of pentavalent vaccine
- EBF = proportion of infant (6-8 months) who were exclusively breast fed for first six months,

1. Morbidity management domain

- Care seeking for newborn complications = proportion of neonates who had complications within first month of last delivery and sought care from ASHA
- ORT = proportion of infants (6-8 months) who had diarrhea within last two weeks and received ORS from ASHA
- ARI/fever = proportion of infant (6-8 months) with ARI/fever within last two weeks and sought care from ASHA

The weight of morbidity domain will be nullified if no morbidity was found for a particular ASHA.

Improve MACCI from 36% to 51%.

Regarding the use of composite indicator, we were guided by Composite coverage index (CCI) which is now widely used to measure coverage of key MNCH interventions and strength of health-system[[37]](#footnote-38),[[38]](#footnote-39),[[39]](#footnote-40). It provides summary measure to assess continuum of care. Formula for calculating CCI is as following.

CCI= 0·25 × (FPS + 0.5 × [SBA + ANCS] + 0·25 × [2DPT3 + MSL + BCG] + 0·5 × [ORT + CPNM])Error: Reference source not found

In which FPS is family planning needs satisfied. Above interventions were selected because of its impact on mortality, measurability, availability of data, relevance with health system strength and as reflection across continuum of care. All above indicators were calculated using standard countdown 2015 definitions [[40]](#footnote-41).

For this study, a modified composite summary measure is required which can reflect (1) coverage of MNCH interventions to be provided by ASHA in India (2) scope of ImTeCHO intervention (3) relevance in India. Therefore, CCI was modified without violating basic concept of CCI. Based on evidence of effectiveness of postnatal home visits by ASHA to reduce neonatal mortality in India and national recommendations from the government, a measure of postnatal home visit was added in the modified MACCIError: Reference source not found. Also, post-natal care for newborns is a countdown 2015 indicatorError: Reference source not found. Considering high prevalence of malnutrition in India, and role of ASHA in promoting young infant feeding practices, exclusive breast feeding up to six months was included in MACCIError: Reference source not found. It is also a countdown 2015 indicatorsError: Reference source not found. Care seeking for newborn was included considering its important association with newborn mortality, role of ASHAs and ImTeCHO’s component for management complicationsError: Reference source not found^,Error: Reference source not found^. Management of ARI/febrile illness was added as it was done during DLHFS which is being used for this study as wellError: Reference source not found. Family planning needs satisfied (FPS) was removed as it FPS is not focus of ImTeCHO intervention. Coverage of measles was removed because the respondents for the endline survey will be limited to mothers of infants between ages of six to eight months. Also, coverage of measles and BCG vaccination is already highError: Reference source not found. Hence, formula for MACCI is designed as following:

Modified ASHA-centric composite coverage index (MACCI) = 0·25 × (0.33 × [Care Complete ASHA home visit during antenatal period +Full ANCS+SBA] + [Complete HBNC+] + 0·5 × [DPT3 + EBF] + 0·33 × [Care seeking for newborn complications+ORT + ARI/febrile illness])

Same weight was given to each of the all four main domains of interventions throughout the continuum of care which includes maternal, newborn, young infant care and care seeking for complications.

## 5.2 Secondary objectives and research questions

Secondary objective 1: Examine effect of ImTeCHO intervention in the form of job aid to ASHA and ANM to increase coverage of care among complicated maternal, newborn and child cases by facilitating referral to a health facility and managing at home for those cases that unable to get referred in tribal areas of Gujarat.

Secondary research question 1: Can ImTeCHO intervention in the form of job-aid to ASHAs and ANM increase coverage of care among complicated maternal, newborn and child cases in tribal areas of Gujarat?

Complete list of set of indicators for secondary objective 1 can be found in Appendix 1.

Secondary Objective 2: Examine effect of ImTeCHO intervention in the form of a job aid to medical officers and PHC staff to improve support and supervision of ASHAs.

Secondary research question 2: Can ImTeCHO intervention in the form of a job aid to medical officers and PHC staff improve support and supervision of ASHAs?

- Proportion of ASHAs whose performance was reviewed by mHealth facilitator (more about mHealth facilitator in section 10.2) over a phone call per month
- Proportion and number of ASHAs who were contacted by mHealth facilitator (mostly through phone) every month for non-adherence to intervention
- Proportion of ASHAs who were visited in field by ImTeCHO facilitator for quality improvement
- Proportion of PHC meetings when [data from] ImTeCHO was used at least once for supervision by PHC staff
- Medical officer’s login and task completion rate
- Average number of phone call by SEWA Rural’s ImTeCHO facilitator per ASHA per month towards providing support
- Proportion of beneficiaries with high risk complications who received guidance from SEWA Rural’s helpline
- Average number of phone call by SEWA Rural’s helpline per ASHA per month towards providing guidance for morbidity management
- Average number of motivational announcements sent by SEWA Rural to ASHAs per month
- Average amount of extra incentives paid by SEWA Rural per ASHA per month

Secondary objective 3: Examine process indicators to understand processes and level of adherence to intervention.

Selected process indicators are listed below and complete list of process indicators are given in Appendix 1. The process indicators will be measured for intervention area only. Please refer to Figure 2 and 2 to see conceptual frameworks which were used to identify process indicators.

| **Sr no** | **Process indicators** |
| --- | --- |
| *Intervention 1: Mobile as a job-aid to ASHAs during her scheduled home visit to increase coverage of selected MNCH interventions* | |
| 1.1 | ASHA attendance rate (login rate)* |
| 1.2 | Number of home visit forms filled using mobile phones against expected (Task completion rate)* |
| 1.3 | Number of pregnancy registration forms filled using mobile phones against expected number of registration |
| 1.4 | Time taken (Mean, median, range) to complete mobile based home visit forms (Rationale: indirect measure of quality of interview) |
| 1.5 | Proportion of live and still births reported on the day of outcome |
| 1.6 | Proportion of beneficiaries who attended VHND against expected number (VHND attendance rate) |
| 1.7 | Line listing of beneficiaries with various due services (eg. ANC examination, HBNC, Vaccination, growth monitoring, ) |
| *Intervention 2: Mobile phone as job aid to ASHA and ANM to increase coverage of care among complicated maternal, newborn and child cases by facilitating referral to a health facility and managing at home for those cases that refuse to get referred* | |
| 2.1 | Number of complicated maternal (severe anaemia), newborn (LBW) and child (severe underweight) cases identified against expected |
| *Intervention 3: mHealth solutions in form a web interface to provide tools and timely information to PHC staff for monitoring and supporting MNCH program* | |
| 3.1 | Proportion of days when web interface was reviewed by medical officer (Attendance of medical officer with use of web interface) |
| 3.2 | Stock-out rate (Proportion of times when a drug or equipment was not available when required. Eg. Non-availability of antibiotics in case of of child with pneumonia) |
| 3.3 | Timeliness of incentive payment to ASHA |

Secondary objective 4: Examine effect of ImTeCHO intervention in the form of job-aid to ASHAs, ANMs, medical officers and PHC staff to increase coverage of selected MNCH interventions and care to be provided for complicated maternal, newborn and child cases in tribal areas of Gujarat.

Complete list of set of indicators for secondary objective 4 can be found in Appendix 1.

## 5.3 Summary of participants, intervention, comparator and outcomes (PICO)

**Table 7: Summary of participants, intervention, comparator and outcomes (PICO)**

|  | **PICO components** | **Brief description** |
| --- | --- | --- |
| 1 | **Participants (complete description to follow in Section 9)** | ASHAs, ASHA facilitators, ANMs, Medical officers, PHC support staff, mHealth facilitators, help line, pregnant women and mothers of infants |
| 2 | **Intervention (complete description to follow in Section 10)** | *ASHA level:* mobile phone as job-aid to increase coverage of MNCH interventions. Job-aid tools that will be used are checklist, reminder, scheduling, videos, and algorithms to promote healthy behaviors.  *ASHA, ANM level:* Mobile phone as job-aid to facilitate care to complicated cases. This will include use of mobile phone as diagnostic aid, facilitate referral by calling emergency transport, send notification to ANM, and obtain management guidelines based on algorithms for those cases that are unable to go to health facility along with reminders to revisit complicated cases.  *Medical officer, ANMs and PHC staff including ASHA facilitators:* Web interface and mobile phone application to provide timely information to medical officer for monitoring and supporting maternal, newborn and child health program. Some of the features on web interface will include daily checklist to track complicated cases, weekly and monthly performance reports, reminders to pay incentives, incentive and supply management and ongoing surveillance data.  mHealth facilitator: This person, from SEWA Rural, will be first contact for ASHAs and PHC staff to report and solve any problem with the ImTeCHO mobile phone/web application and other related issues. The mHealth facilitators will monitor adherence to the intervention through use of set of process indicators. The mHealth facilitator would visit ASHAs in field to solve any technology issues and visit beneficiaries along with ASHAs for ongoing training/supervision/motivation. This person will not be involved in patient care and will not get in existing working arrangement within the govt. This person will facilitate use of ImTeCHO in government primary health care system only.  Help line: A counselor from SEWA Rural would provide help line services over phone. The help line would proactively contact and counsel all beneficiaries and ASHAs (ANMs and medical officers, if needed) to provide guidance for management of complications, including ensuring referral. ASHAs will be given help line number so that they can contact in case of any help required. |
| **3** | **Comparator (complete description to follow in Section 11)** | Usual practice in control area where current level of services provided by the government and other agencies will continue. |
| **4** | **Outcome** | - Improve proportion of neonates/mothers who received at least two postnatal home visits within first week of delivery by ASHA from 46% to 66% - Improve MACCI from 36% to 51% |

# 6. Study design

## 6.1 Design

This will be a two arm, parallel, stratified cluster randomized trial in which unit of randomization will be a PHC. Stratification is required to ensure that prevalence of primary outcomes are similar in the intervention and control groups along with almost equal selection of PHCs from from the most backward Dediyapada block. Stratification will be also done to improve power and precision. The randomization will be done after baseline survey. Along with primary outcomes, randomization would help to balance cluster size across both groups. The allocation ratio will be 1:1. Stratified randomization will be done by a statistician at AIIMS not involved in implementation of intervention. The baseline variable to be used for stratification will be the primary outcome indicator MACCI. The PHCs in each of the two strata will be randomly allocated into to intervention and control group. The randomization will be done using software nQuery. Due to the nature of the community-based intervention, blinding will not be possible.

Assessed for inclusion: 26 clusters

Randomized: 22 clusters

Did not meet inclusion criteria: 4 clusters

**Allocated to intervention**

11 clusters

Average number of ASHAs per cluster:

Average population size:

Range:

Enrolled for baseline survey: n1

**Allocated to intervention**

11 clusters

Average number of ASHAs per cluster:

Average population size:

Range:

Enrolled for baseline survey: n1

Enrolled for baseline survey: n2

**Follow up**

Clusters:

Lost to follow up (n= )

Discontinued intervention (n= )

ASHAs:

Lost to follow up (n= )

Discontinued intervention (n= )

Newly recruited

New pregnancy registrations: (n= )

Live births: (n= )

**Follow up**

Clusters:

Lost to follow up (n= )

Discontinued intervention (n= )

ASHAs:

Lost to follow up (n= )

Discontinued intervention (n= )

Newly recruited

New pregnancy registrations: (n= )

Live births: (n= )

**Analysis**

Clusters:

N clusters (number of clusters excluded)

Discontinued intervention (n= )

ASHAs:

N ASHAs (number of clusters excluded)

Enrolled for endline survey: n2

**Analysis**

Clusters:

N clusters (number of clusters excluded)

Discontinued intervention (n= )

ASHAs:

N ASHAs (number of clusters excluded)

Enrolled for endline survey: n2

**Figure 1 Enrolment and outcomes**

## 6.2 Rationale for design

Randomization is required to minimize risk of bias and to attribute effect to the intervention. Considering complex community based intervention, cluster randomization is required. The ImTeCHO web interface will be primarily used by the PHC staff, including medical officer, and other support staff; thus, unit of randomization will be a PHC to reduce contamination between clusters. A PHC is operational and administrative unit for providing curative and public health services for all villages covered by it. Each PHC has a team of two medical officers and 4 to 6 ANM along with one Lady Health Visitor (LHV) and male multi-purpose workers. Out of two medical officers, one has MBBS degree and is in-charge of PHC, including clinical services. Other medical officer is usually an AYUSH (Ayurveda, Yoga & Naturopathy, Unani, Siddha and Homoeopathy) and is involved in field based duties. Each ANM is in-charge of one sub center which serves population of 3,000 to 4,000. On an average, there are 20-25 ASHAs per PHC. There is one ASHA Facilitator for approximately every 10 ASHAs. Average size of population per PHC is approximately 20,000 in tribal areas[[41]](#footnote-42).

## 6.3 Inclusion and exclusion criteria

All clusters (PHCs) belonging to Valia, Netrang (except those where ImTeCHO is being implemented already as part of another project), Dediyapada, Nandod, Garudeshwar and Tilakwada blocks with 100% rural population and scheduled tribe population of more than or equal to 45% will be eligible to be included. Those PHCs will be excluded where all medical officer posts, and 20% post for ASHAs are vacant at the time of initiation of study. Those PHCs will be excluded whose more than 10% villages have no mobile signal most of the time. Although ImTeCHO mobile application can function without GPRS signal, lack of such signal in large areas of intervention will affect components of intervention to significant extent. PHCs where internet cannot be accessed reliably by medical officer and PHC staff to view web interface and an alternative arrangement is not possible will be excluded too as web interface is important part of the ImTeCHO intervention.

# 7. Study setting

This study will be conducted by SEWA Rural, which is a voluntary organization located in Jhagadia block of Gujarat. SEWA Rural is a voluntary development organization involved in health and development activities in rural, tribal area of Gujarat, India since 1980. From April 2003 to March 2011, SEWA Rural implemented a family centred safe motherhood and newborn survival project covering 171,000 population of Jhagadia block. This project included comprehensive community level interventions which were complemented by First Referral Unit (FRU) providing Comprehensive Emergency Obstetric and Newborn Care (CEmONC) services. Supported by the John D. & Catherine T. MacArthur Foundation, the main goal of this project was reduction of maternal and neonatal mortality and morbidity. There was 75% reduction in maternal mortality and 38% reduction in newborn mortality over project periodError: Reference source not found. A new, unexplored area in Gujarat will be required for conducting this research. We have identified four blocks as potential study area (intervention and control area); all being predominantly tribal. Area profile of all four blocks is described in Table 8. There are total 26 PHCs in these four blocks.

**Table 8: Area profile of Valia, Sagabara, Nandod and Dediyapada blocks (Census 201**1)

|  | Valia | Dediyapada | Nandod | Tilakwada | Netrang | Nandod | Garudeshwar |
| --- | --- | --- | --- | --- | --- | --- | --- |
| Number of Households | 31,591 | 35,271 | 50,839 | 13,240 | Newly created after census 2011 | 50,839 | Newly created after census 2011 |
| Average Household Size(per Household) | 5 | 5 | 5 | 5 |  | 5 |  |
| Population-Total | 145,400 | 174,449 | 241,053 | 63,871 |  | 241,053 |  |
| Population(0-6Years) | 17,281 | 27,670 | 27,919 | 7,927 |  | 27,919 |  |
| Sex Ratio | 962 | 977 | 935 | 924 |  | 935 |  |
| Sex Ratio(0-6 Year) | 961 | 961 | 923 | 891 |  | 922 |  |
| SC Population | 1,038 | 498 | 5,645 | 1,965 |  | 5,645 |  |
| ST Population | 113,771 | 168,181 | 176,658 | 35,396 |  | 176,658 |  |
| Sex Ratio (SC) | 1,031 | 844 | 976 | 919 |  | 976 |  |
| Sex Ratio (ST) | 967 | 979 | 940 | 922 |  | 940 |  |
| Proportion of SC (%) | 1 | 0 | 2 | 3 |  | 2 |  |
| Proportion of ST (%) | 78 | 96 | 73 | 55 |  | 73 |  |
| Literates | 93,707 | 94,725 | 166,198 | 44,215 |  | 166,198 |  |
| Illiterates | 51,693 | 79,724 | 74,855 | 19,656 |  | 74,855 |  |
| Literacy Rate (%) | 64 | 54 | 69 | 69 |  | 69 |  |

Valia block belongs to Bharuch district whereas Dediyapada, Nandod and Sagbara blocks are in Narmada district. The four blocks belong to eastern, tribal belt of Gujarat; this tribal belt stretches along the Satapuda mountain range which begins from Dahod in north to Dharampur in south. Most of the Dediyapada and Sagabara areas are covered by thick forest. Ahmadabad is the nearest city with an international airport which is located approximately 250 km from study area. Sagabara, Nandod and Dediyapada borders Maharashtra state on eastern side. Valia’s western part, due its proximity to mega-industrial area of Ankleshwar is rapidly getting urbanized whereas Dediyapada, Sagabara and most of the Nandod have remained rural and remote. The four block capitals are well connected by road to national highways though there are no existing railway facilities in any of the 4 blocks though block capital of Nandod will soon be connected via railways. Block headquarters-Valia, Nandod, Dediyapda and Sagabara are approximately 40 km, 60 km, 60 km and 80 km from SEWA Rural head-quarter which is located in Jhagadia town.

The tribal population residing in the study area is called “Vasava”. As with other tribal communities, Vasava tribe has its own language and customs which are different from mainstream culture though this is changing rapidly as they are increasingly connected with mainstream culture. Agriculture is the main occupation with large number of population working as landless laborers. The rate of industrialization is much slower in case of Dediyapada, Nandod and Sagabara compared to other parts of Gujarat. Dediyapada, Nandod and Sagabara belong to Narmada district which is named one of the *most backward districts* by the Ministry of Panchayati Raj in 2006 considering the lack of development and difficult terrain[[42]](#footnote-43).

# 8. Sample size calculation

Please see Table 9 for sample size calculation and associated assumptions. Based on assumptions listed in the Table 9, eleven clusters (PHCs) will be randomized in each arm with which we will be able to answer the above mentioned questions. The assumptions used for sample size calculation are based on data collected by SEWA Rural in a sample of study area and current area of pilot implementation along with other relevant wide scale surveys by the government. However, there is no existing data available from the whole study area about primary outcome of interest. Hence, sample size will need to be revised based on findings of baseline survey and definition of “success” if large discrepancy is found between current assumptions and baseline survey findings. Considering required sample size of 11 PHCs each in intervention and control area, six blocks (total 26 PHCs, out of which 23 meet all eligibility criteria) listed above should suffice to select study clusters.

As intervention is primarily affecting ASHAs, analysis will be done at ASHA level. Therefore, number of ASHAs per PHC will be used as “cluster size”. Based on indirect information before baseline, we assumed there will be 25 ASHAs in a PHC and MACCI of 36%. In absence of existing information regarding the intraclass correlation (ICC), we will assume ICC to be 0.02. Assuming loss of one cluster per arm and three ASHAs per PHC, for detecting 15% absolute improvement in MACCI in intervention arm compared to control arm at endline survey with 80% power and 5% two-sided significance level, we estimated that required sample size per arm will be 11 PHCs/clusters. Similarly, we assumed 46% of neonates/mothers would receive at least two postnatal home visits within first week of delivery by ASHA in control arm. Assuming loss of one cluster per arm and three ASHAs per PHC, we estimated that required sample size per arm will be six PHCs/clusters for detecting 20% absolute improvement in proportion of neonates/mothers who received at least two postnatal home visits within first week of delivery by ASHA in intervention arm compared to control arm at endline survey with 80% power and 5% two-sided significance level..

**Table 9: Sample size calculation and associated assumptions**

|  |  | Ave value in control | Expected outcome in intervention | Ave number of individual per cluster | Number of cluster required |
| --- | --- | --- | --- | --- | --- |
|  | Proportion of neonates/mothers who received at least two postnatal home visits within first week of delivery by ASHA | **0.46** | **0.66** | **22** | **6** |
|  | MACCI (based on following assumptions) | **0.36** | **0.51** | **22** | **11** |
|  | Complete antenatal examination[[43]](#footnote-44) | **0.45** | **0.6** | **22** |  |
|  | At least 3 ASHA visits during pregnancyError: Reference source not found | **0.65** | **0.9** | **22** |  |
|  | Skilled birth attendanceError: Reference source not found | **0.85** | **0.90** | **22** |  |
|  | Neonates/mothers who received the recommended number of postnatal visitsError: Reference source not found | **0.05** | **0.30** | **22** |  |
|  | Exclusive breast feeding[[44]](#footnote-45) | **0.5** | **0.7** | **22** |  |
|  | DPT3 coverageError: Reference source not found | **0.67** | **0.67** | **22** |  |
|  | Sought care from ASHA for newborn complicationsError: Reference source not found | **0.27** | **0.5** | **10** |  |
|  | Sought care from ASHA for fever/ARIError: Reference source not found | **0.27** | **0.50** | **6** |  |
|  | Received ORS from ASHA for diarrhea within last two weeksError: Reference source not found | **0.65** | **0.75** | **6** |  |

# 9. Study participants

## 9.1 ASHA

Detailed description of ASHA is provided in Section 2.2. Usually, the norm is to have one ASHA for 1,000 populations. There is to be relaxed for tribal and hilly areasError: Reference source not found. Considering the study area which is primarily tribal, there will be approximately 250 to 300 ASHAs in the intervention area. Most of the ASHAs are expected to belong to tribal community.

## 9.2 ASHA facilitator (AF)

ASHA facilitator is responsible for monitoring, facilitating and providing supportive supervision of ASHAs. AF performs above duty during her regular field visit. There is one AF for every 10-20 ASHAs, AF is a link between ASHAs and block level support structure[[45]](#footnote-46).

## 9.3 Auxiliary Nurse Midwife(ANM)

ANM is a qualified health provider who provides vital primary health care services including related to MNCH. Description of ANM is provided in section 2.2. In regards to ASHA programme, ANM has following role: ANM makes home visits and provide care to those complicated cases who are unable to go to a health facility. Such care will include confirming the diagnosis, encouraging referral, counselling, and administering drugs, including oral antibiotics. Such function is already a job responsibility assigned to the ANMsError: Reference source not found^,Error: Reference source not found^.

ASHAs are expected to identify, refer and escort complicated maternal, newborn and child cases to a health facility. For those complicated cases that are unable to go to health facility, ANM is expected to make home visit and provide treatment. ANM is allowed to provide range of drugs, including intramuscular antibiotics injections[[46]](#footnote-47). Along with AF, ANM also provides supportive supervision and support to ASHA. ANM verifies ASHA’s performance report which is used for calculating ASHA’s performance based incentive.

## 9.4 Medical officer

Detailed description of medical officer is provided in Section 2.2. medical officer. The medical officer is overall in-charge of PHC. The medical officer’s main responsibilities are to provide curative service, manage promotive and preventive programs, give training, monitor field level health workers and oversee administrative tasksError: Reference source not found. There will be at least 20 medical officers who will participate in this study.

## 9.5 mHealth facilitator (mHF)

mHF is a nodal person from SEWA Rural who will be facilitating and assisting above participants for using ImTeCHO. There will be one mHF for every two PHC. Detailed responsibility of mHealth facilitator can be found in section 13.1.4.

## 9.6 PHC support staff

Along with medical officers, ANMs, and AF, there are other members of PHC staff who play supporting role for ASHA program. These staff-members are data-entry operators, pharmacist, supervisors, and multi-purpose male health workers. PHC support staff are involved in calculation of ASHA’s performance based incentives, and replenishment of supplies.

## 9.7 SEWA Rural helpline/ telephone care

A helpline is established at SEWA Rural. The helpline will provide support to ASHAs to manage complicated cases over phone usually after receiving alerts from the ASHAs. The support would involve confirmation of diagnosis made by ASHA, counseling families to seek care at higher referral facility, adopt household level practices to care of complications and alerting medical officer in case of emergency cases.

## 9.8 Pregnant women and mothers of infants

Pregnant women and mothers of the infants will be the direct beneficiaries of the intervention described above. They will be respondents for the baseline and end line surveys which will be described in details in section 12.

# 10. Description of intervention and how it contributes to reducing mortality and morbidity through improving delivery of MNCH interventions

## 10.1 Conceptual framework

### 10.1.1 Mobile phone as job aid to increase coverage of MNCH care

As indicated in Section 2.2, large amount of ASHA’s job regarding MNCH is related to promoting healthy behaviors at household level. Conceptual framework for using mobile phone as job aid to ASHAs to increase coverage of MNCH is below in Figure 2. As indicated in Figure 2, ASHA (agent of change) will promote the households and pregnant women to adopt healthy behaviors (care seeking, breast feeding, and compliance with IFA etc). Mobile phone technology will play important role at every step of this process as indicated in the figure.

ASHA

Behavior change transaction (home visits)

Household

Adoption of healthy behaviors

ImTeCHO to address each constructs

Identify epidemiologically targeted key behaviors

Checklist on mobile phone to remind ASHAs to address every key behavior

Identify target groups

Checklist and reminder to involve individuals in target group (mother-in-law, husband etc) during visit

Develop behavior change transactions

- Contextual information

- Motivation

- Behavioral skills & self-efficacy

###

- Reminder alerts to schedule home visits

-Use of videos, images highlighting contextual information and improve self-efficacy

- Checklist to identify and address barriers to behavioral change

- Checklist to address external motivating factors (cash incentives etc)

Build mechanisms to sustain & institutionalize new behaviors

**Figure 2 Conceptual framework for increasing coverage of maternal, newborn and child care by ASHAs using mobile phones as job aid (Adopted from *Behavior change for newborn survival in resource-poor community settings: Bridging the gap between evidence and impact[[47]](#footnote-48)*)**

### 10.1.2 Mobile phone as job-aid to facilitate care to complicated cases

As indicated in Section 2.2, one of the important part of ASHA’s job regarding MNCH is to identify complication, risk stratify, refer cases with severe complications after providing first contact care and provide home based care for minor complications. As seen in Figure 3, mobile phone technology can assist ASHAs at every step of this process.

Management of morbidity by qualified health personnel

ASHA

Maternal, newborn, or child case with complication

Case management transaction

Identification of complication

Risk stratification and referral of cases with severe complications

Home based care of cases with minor complications (moderate anemia, feeding problems etc) and severe complications that are unable to go to health facility

ImTeCHO to address each constructs

Checklist and In-built algorithms to identify complications

Automatic risk stratification and tool to facilitate referral

- Notification alert and schedule home visit for ANM

- Manage complication by displaying customized management guidelines based on diagnosis

- Schedule follow visit of ASHAs to check status and compliance with treatment

**Figure 3 Conceptual framework for increasing coverage of care of maternal, newborn and child cases with complications using mobile phones as job aid**

###

## 10.2 Intervention:

1. **Use of ImTeCHO mobile and web based application by ASHAs, medical officers and PHC staff**

Please see Figure 4.

**Mobile phone as job aid to ASHA to increase coverage MNCH care**

- Schedule reminder to ASHAs to make home visit
- Home visit forms having checklist to remind scheduled tasks
- Multimedia to improve counseling
- Checklists to assess and address barriers to behavior change of families (e.g. birth preparedness, complication readiness)
- Checklist to remind tasks to be performed during VHND
- Log cases for un-expected events such as death, migration, early termination of pregnancy

**Mobile phone as job aid to ASHA and ANM to facilitate care for mother, newborn and child with complications**

- Diagnostic tool: Checklist and In-built algorithms to identify mother or newborn with complications and automatic risk stratification
- Tool to facilitate referral to functional referral facility (for calling 108/emergency transport etc)
- Notification alert for ANM once complicated case is identified by ASHA
- Manage morbidity by displaying customized management guidelines on mobile phone based on diagnosis to help ASHA manage complicated cases at home who refuse get referred to health facility
- Checklist for ASHAs to identify minor morbidity and in-built algorithm to manage such morbidity (anemia, breast-feeding issues, LBW mgmt)
- Help line/telephone care

INTERVENTION

**Web interface to provide timely information to medical officer to facilitate monitoring and supporting program**

- Daily follow-up of selected high risk cases
- Performance monitoring
- Monthly reports
- Supply management
- Calculation and timely payment of incentive to ASHAs
- Announcements
- Motivating ASHAs as the system will display a message about incentive earned after delivery of a service

**Figure 4 Intervention**

Please refer to table 6 to compare current situation and proposed mHealth solutions to be used in intervention.

At front end, there are 2 components:

1. At the mobile end- for ASHAs, ANMs, and

2. at the web end- for PHC staff, including medical officers, AF, ANM.

### 10.1.1 Mobile phone as job aid to ASHAs to increase coverage of maternal, newborn and child care

Every ASHA will be given a low cost phone which will be General Packet Radio Service (GPRS) enabled and have multimedia feature available. Based on SEWA Rural’s and Argusoft India Ltd’s research, Nokia ASHA 3115 which costs Rs. 6,000 (Rs, 6,500 with all accessories) will be used for this study. Mobile phone application will have following features: Home visit forms, log cases details, work log, announcements, SMS information channel. Detailed description of these features will follow in next paragraph. ASHA will fill out forms on her mobile during home visits. Data will be sent using GPRS network to a server where data will be stored. In case GPRS is not available, data will be stored in mobile phone and it will be sent to server when GPRS becomes available; thus, data will not be lost even in absence of GPRS. Data entry time will be “time stamped”.

Each mobile application will have following features:

- Home visit forms: These forms will be prepared based on existing forms and jobs that ASHAs need to complete. The forms will have checklists to remind ASHA to perform tasks that she is expected to perform. Some of the checklists to be used during antenatal period include registration information, educational information (regarding aspects of birth preparedness, complication readiness, nutrition counseling, danger sign recognition and essential newborn care), assessment for complications, assessment of barriers for behavior change and actionable information (reminders to get ANC check-up). Some of the checklists during postnatal period include assessment of complications (maternal, newborn and child complications), assessment of barriers for behavior change and educational information regarding essential newborn care (breast feeding, cord care, thermal care, danger sign recognition etc). There will be features to ensure data quality by not allowing ASHA to skip a question and only allowing logical entries. The application will have features to enhance truthfulness of information entered. These features include time stamp, audio and photograph of the client, and GPS tracking.
- Multimedia for behavior change communication and generating demand among beneficiaries: Selected multimedia will be integrated in the home visit forms. Some of the multimedia features that will be used include videos for counseling, images of danger signs, and voice prompts. To generate demand in community for health services, multimedia will prepared on topics such as danger sign recognition, services available at VHND, benefits of delivering in institution, advantage of immunization, and prepare beneficiaries for potential complications. These videos are created for behavior change communication with intended behavior being seeking care from health workers.
- Scheduled reminders and alerts: The system will generate a schedule once a pregnant woman is registered. The system will send schedule reminders and work plan for next 7 days to ASHA once a week. The system will keep sending reminders until the schedule visit actually occurs. Apart from regular scheduled home visit, extra visits will be scheduled in case a complication is diagnosed.
- Log cases details: This feature will assist ASHA to deal with un-expected and un-scheduled events such as death, interim visit for a problem, early termination of pregnancy and migration. Migration among pregnant women is common in study area; therefore, the application will provide flexibility to ASHA to transfer out and transfer in migrated cases.
- Announcements: ASHA will be able to receive announcements from PHC staff using this feature. Such announcement could be to inform about upcoming meeting etc.
- SMS information channel: This feature is to confirm certain actions that PHC staff might have taken to support activity of that particular ASHA. This includes mainly includes payment of incentive. ASHA will receive an alert when the PHC staff enters information on web interface stating that incentive has been paid. ASHA will have to respond to this alert to confirm such event.
- Administrative forms: these forms will include information that ASHA will enter regarding her performance for non-MNCH activities for calculating her incentives. Also, status of drug kit will also be entered by ASHA. ASHA will be asked to provide information about incentives received during previous month.

### 10.1.2 Mobile phone as job aid to ASHAs and ANMs to facilitate care of cases with complications

Mobile application will be designed to help with identification and referral of complicated cases along with management at home, in case required. Following is the description of how intervention will be used to perform above tasks.

- Mobile as diagnostic tool and risk stratification: ASHAs will receive scheduled alerts to make home visits. Home visit forms will have checklist to assess symptoms and signs which is required to perform diagnostic assessment. Mobile application will have logic to derive at a diagnosis which will be based on ASHA training module. Based on how the checklist is filled, a diagnosis will appear at the end of the form. Each diagnosis will be risk stratified in to one of the 3 categories: Severe complication (“Red alert” in process flow diagram) for which emergency referral is required, mild complication (“Yellow alert” in process flow diagram) for which care can be provided by ASHA at household level and no complications (“Green alert” in process flow diagram) for which no input is required.
- Tool to facilitate referral: ASHA will use mobile to facilitate referral of maternal, newborn and child cases with severe complications. ASHA can use mobile phone to call emergency transport services. For every case with severe complication, ASHA will receive alert everyday for next three days after diagnosis to remind her to revisit the complicated case to check whether the patient has actually left the village to go to a health facility and provide care in case patient is still at home.
- Tool to provide care by ANM: ANM will be notified as soon as a complication is diagnosed by ASHA. Based on complicated cases identified over last 24 hours, ANM’s schedule will be automatically generated. ANM will visit only those complicated cases at home that had refused to get referred. ANM will fill out a home visit form on ASHA’s mobile which will include a check list to confirm diagnosis, counsel to get referred and give treatment in case patient continues to refuse referral. Treatment will be displayed on mobile phone once diagnosis is confirmed. Treatment will be based on accepted guidelines for ANMs.
- Tool to provide care by ASHA: Mobile phone will display management guidelines to be implemented by ASHA under the guidance of ANM for those patients who have mild complications and those with severe complications who are unable to get referred. Scheduling alerts will remind ASHAs to re-visit the patient to assess status and compliance with prescribed treatment. These management guidelines will be mainly based on ASHA module 6 and 7.

### 10.1.3 Web interface as job aid to medical officer and PHC staff for monitoring and supporting program

Web interface is a computer screen where organized data is available at web-end for PHC staff. Data entered in mobile by ASHAs and ANM is transferred to a server using GPRS. The system organizes the raw data in to useful information (based on predefined logic) for PHC staff. Variety of filters will be available related to time, location and type of information needed. The web interface will be protected by password. Depending on the user, variety of restriction will be applied to allow restricted access to the information available online. The web interface will have 3 features.

- Individual case record: this will be available for all cases registered by the ASHA.
- Tracking of high-risk cases: Critical information about all complicated cases will be displayed separately on web interface so that they can be followed-up by PHC staff. Some of the critical information will include name and diagnosis of the case, current status (live or deceased), referral status and whether FHW and ASHA are making scheduled home visits in case the patient has refused to get referred. Detailed individual medical record will be available for all cases. The medical officer will review all high-risk cases on daily basis and will complete a checklist to ensure follow up actions are taken for all cases that have stayed at home.
- Reports: The system will automatically aggregate and tabulate data to create useful reports for medical officer and PHC staff. Report will be regarding health outcomes, performance of health workers, MCTS report, along with regular subcenter and PHC level reports such as form 6, 7 and 7A. Reports can be either downloaded by medical officer or will be emailed to him/her on monthly basis. The performance reports can be used during regular monthly PHC meetings to monitor health workers’ performance.
- Incentive, supply and migration management: The web interface will assist medical officer and PHC staff to support ASHA in her day to work by making incentive, supply and migration management easier. A “stock inquiry” alert will be sent to all ASHAs every month. This stock inquiry form will ask ASHAs about remaining balance of stock with her. If a particular stock is approaching zero than an alert will be sent to medical officer and PHC support staff, which will ensure supply of this particular item. Additionally, incentives will be automatically calculated by the system at the end of the month. The medical l officer will review the amount of incentive to be paid to ASHA and approve it online. The accountant will keep the incentives ready on the day of monthly PHC meeting so that ASHA will receive timely and complete incentive. Checklist will be used to ensure that above tasks are completed on time.
- A mobile based application is being developed for ANMs and/or AFs for better supportive supervision of ASHAs.

1. **Help line/telephone care from SEWA Rural**

Complete details are available in section 13.1.4.

1. **mHealth facilitators from SEWA Rural (one for every two to three PHCs)**

Please see section 5.3 for complete details.

1. **Training, and mentoring for use of mobile phone and refresher training for module 6 and 7**

- 10 to 7 day training of ASHAs which include refresher training for ASHA module 6/7 and training to use ImTeCHO. This may be divided into two phases. In most cases, training will take place in the district where program is being implemented.
- 2 to 3 days training of PHC staff for use of ImTeCHO web interface and mobile application. This may be divided into two sessions.

1. **Project team at SEWA Rural and district health societies**

- Monthly email and SMS to medical officer and Block Health Officer/Chief District Health Officer about their login and task completion rates.
- Intervene in emergency situation
- Supervise and support ImTeCHO facilitators, program coordinators and help line
- Coordinate and linkages with medical officers and higher level health officials
- Developing and improvising software application with help of the Argusoft India Ltd

As with other implementation research studies, the intervention may need undergo some minor changes based on the feedback received from the health workers during the course of the study. This is an acceptable or rather desired process in the implementation science.

## 10.3 Phases of intervention

Detailed description of implementation of intervention can be found in the section 13.1 of project plan and management. Table 10 shows phases of implementation of intervention. Development of ImTeCHO intervention has been already completed and formative evaluation is currently underway.

There will be 6 phases:

1. Phase 1 (Preparations): The recruitment and training of project staff along with revisions in protocol based on technical help from the experts from the ICMR and WHO will be done.
2. Phase 2 (Baseline data collection- 0-2 months): Baseline data will be collected from intervention and control area. Findings will be used for randomization to be done at the end of this phase.
3. *Phase 3 (Training and maturation phase- 5 - 10 months):* All ASHAs (approximately 250 in number) and PHC staff will be trained to use mobile phone and web interface respectively to implement intervention. After training, ASHAs will require extra support for initial weeks to get them used to working with mobile phones and initial technical troubleshooting. Based on SEWA Rural’s recent experience with introduction of mobile phone technology, it is essential to have adequate time period for maturation phase so that intervention become fully functional at the end of the maturation phase. All the ASHAs from control area will receive refreshers’ training during this period as well. The PHC staff will be trained for using web and mobile based application.
4. *Phase 4- Intervention implementation- 11-36 months):* At village level, existing ASHAs within the government set-up, who will be trained during the maturation phase, will use mobile phone to implement the intervention. Medical officer and PHC staff will use the web interface to monitor and support program.
5. Phase 5 (Endline data collection- 26-31 months): Endline data collection from intervention and control area will take place during this phase.
6. *Phase 6 (analysis and report writing- 32-36 months):* Data will be analyzed and complete report will be completed.

Table 10 Phase wise deployment of intervention

|  | Preparations | Baseline survey | **R**  **A**  **N**  **D**  **O**  **M**  **I**  **Z**  **A**  **T**  **I**  **O**  **N** | Training & Maturation phase | Intervention Implementation | Endline survey | Analysis & write-up |
| --- | --- | --- | --- | --- | --- | --- | --- |
|
|
|
|
|
|
| 0-2 months |  |  |  |  |  |  |
| 3 - 4 months |  |  |  |  |  |  |
| 5 - 10 months |  |  |  |  |  |  |
| 11 - 36 months |  |  |  |  |  |  |
| 26 - 31 months |  |  |  |  |  |  |
| 32 - 36 months |  |  |  |  |  |  |

Duration of study will be 36 months including time for analysis and report writing.

# 11 Control arm (Comparator)

The control area will continue to receive usual health services from government and other providers. All ASHAs in control and intervention area will be trained to get up-to-date to provide recommended maternal, newborn and child care. ASHAs will receive refreshers’ training based on ASHA module-6 and 7 which provides skills for the maternal care services that she is expected to deliver including counselling pregnant women, ensuring complete antenatal care through home visits and enabling care at monthly Village Health and Nutrition Days (VHND), assisting households to make birth plan and supporting households for safe delivery. Regarding newborn care, ASHAs will be trained to undertake at least 6 post-partum visits, counsel and problem solve on breastfeeding, keep the baby warm and identify and do basic management of LBW (Low Birth Weight) and pre-term baby, perform examinations needed for identification/first contract care for sepsis and asphyxiaError: Reference source not found. Size of control area will be same as intervention area (11 PHCs).

Currently, there are two e-health initiatives underway in both control and intervention areas. First one is provision of SIM card with CUG (Common User Group) at free of cost to all ASHAs. The government recharges balance of SIM on monthly basis. The ASHAs are expected to use SIM for making phone calls for patient care and other responsibilities. Mobile phones are not provided. Second initiative is Online Mother and Child Tracking System (MCTS) which is described in detail in section 2.3.

To avoid the duplication of data being entered separately into the MCTS and ImTeCHO systems, active efforts are being made to make both IT systems interoperable. There have been discussions with the state government officials to obtain necessary approval. The investigators are in contact with the developers of the MCTS, National Informatics Center (NIC), whose representatives have visited SEWA Rural to discuss this matter. The ImTeCHO system has been developed to ensure potential interoperability. We await the final decision by the state government in this regards.

The MCTS and ImTeCHO generates various reports for the PHC staff; this raises concern regarding duplication and increase in work load. However, the ImTeCHO intervention is developed so that it complements the MCTS reports. The ImTeCHO produces reports and alerts to inform the medical officer regarding a newly diagnosed high risk case; this report is not available in the MCTS. Similarly, there other reports which are not available in the MCTS and can be found only in the ImTeCHO web interface; thus, minimising duplication. Additionally, the reports from the ImTeCHO is created based on the data entered by ASHAs, whereas, the MCTS generates reports based on the data entry operator at the PHC. Therefore, there will be no additional burden at the PHC level towards entering data for generating the reports in the ImTeCHO intervention.

# 12 Measurement of outcomes and follow up

**Table 11 Measurement of outcomes**

| **What** | **Where** | **When** | **How** | **By Who** |
| --- | --- | --- | --- | --- |
| Coverage of proven maternal and newborn interventions, coverage of care among complicated maternal and newborn cases | At household level in community | 2 times: Pre-intervention (baseline), Post-intervention (end line) | Household survey as reported by mothers of infants age 1 to 3 months* | Data collection team |
| Coverage of proven young infant health interventions, coverage of care among complicated young infant cases | At household level in community | 2 times: Pre-intervention (baseline), Post-intervention (end line) | Household survey as reported by mothers of infants age 1 to 3 months | Data collection team |
| Adherence to intervention and Support and supervision received by ASHAs | From ImTeCHO program data (online) | Post-intervention (end line) | From web interface & log of ImTeCHO facilitator | Investigators |

* Mothers of infant age 6 to 8 months will be surveyed at baseline

*Data collection for all research questions*

Household surveys will be used for assessing set of indicators for primary objective, secondary objective-1 and secondary objective-4. The household survey tools is based on draft tool for district level health and facility survey (DLFHS-4) which is available online[[48]](#footnote-49). The survey tools are included in Appendix 2. Respondents will be

- For maternal, and newborn health interventions: All woman who are native of study village and is mother of an infant who is one to four months old at the time of survey will be considered as respondents for evaluation. All native women who resided most of the time (at least five months) in study cluster during antenatal period will be included. All women must be in the study cluster at the time of birth in case of home deliveries and in case of institutional deliveries if the she went to hospital from study cluster and came back to same study cluster immediately after delivery, and who provided consent for the evaluation will be included. All women who resided in study cluster for most of time during first month after delivery will be included. Women whose infant died before the survey will be excluded. Respondents will be identified through ongoing pregnancy and mortality surveillance which described later in this section.
- For child health interventions: All adult woman who are native of study village and is mother of an infant who is six to eight months old at the time of survey will be considered as respondents for evaluation. Women whose infant died before the survey will be excluded.
- For indicators reflecting supervision and support (secondary research question 2 and 3) received by ASHAs in the intervention area, program data will be used from the ImTeCHO web interface and documentation of mHealth facilitators.

All pregnancy registrations, its outcomes, any newborn and infant deaths in all clusters throughout the study period will be counted as part of ongoing pregnancy and mortality surveillance. For that, outcome of all pregnancies will be determined to ensure complete counting of all newborn deaths. This will be done by data collection team by conducting house to house survey of whole study area on regular basis. All live births to woman who are native of study village will included irrespective of place of the birth.

*Follow up*

All new pregnant women and their neonates will be enrolled through ongoing pregnancy and mortality surveillance described above. Each pregnant woman and neonate will be followed up to one year after pregnancy outcome in both arms unless the woman or neonate dies or family migrates out of study area. All clusters and ASHAs will be followed throughout the study by the team of data collectors and investigators.

# 13 Project plan and management

## 13.1 Implementation of intervention

### 13.1.1 Pre-intervention baseline household survey

- Identify validated tool such as DLHFS and modify as per need
- Train data collection team
- Pre-test data collection tool
- Create database
- Do a survey using smart-phone data collection application

### 13.1.2 Training and intervention maturation

There will be two kinds of training: refreshers’ training and secondly training of users to use ImTeCHO. SEWA Rural will provide training.

**Table 12 Type and duration of training**

| Type of training | Trainees | Duration | Timing |
| --- | --- | --- | --- |
| ASHAs’ Refreshers’ training for ASHA module 6 & 7 | All ASHAs of control and intervention area | 3 days | Training phase (5-10 months) |
| ASHAs’ training to use ImTeCHO application | All ASHAs of intervention area | 3-4 days | Training phase (5-10 months) |
| Accreditation followed by getting on production server | All ASHAs of intervention area | 3 days | Training phase (5-10 months) |
| PHC staff training to use ImTeCHO web interface and mobile application along with refresher training | All PHC staff of control and intervention area | 2-3 days | Training phase (5-10 months) |

There is a strong rationale for the requirement of “intervention maturation” phase. Using mobile phone technology requires not only acquiring new skills but organizational change about performing same tasks differently with use of technology. Such change will need to be nurtured and matured before its effectiveness can be evaluated. Based on SEWA Rural’s experience with use of mobile phone technology by frontline workers, each ASHA will have to undergo 3 phases during maturation period: initial training (approximately 3 days), intensive support for first month after training (on the job support) and maintenance support for rest of the intervention (in form of mHealth Facilitator). Considering the scale of intervention area, approximately four months will be required. Also, each ASHA needs to use mobile phone at reasonable times in field setting to get used to the newly acquired skill. Considering small number of pregnant women and newborn in a particular village, few months will be required to get her used to making home visits using mobile phones. Considering small number of training and support staff, training will need to occur in phases instead of all at one time.

The PHC staff will also need to get used to the new system. After initial training (of approximately 2-3 days), extra support will be required for first week to handle information about high risk cases and for downloading and using monthly reports to conduct performance monitoring. Also, support will be required to get PHC staff used to alerts related to supply management and payment of incentives.

### 13.1.4 Implementation of ImTeCHO intervention and field organization

As this study does not assign any new tasks to ASHAs beyond envisioned by the government, she will continue performing her duties as before except now she will use mobile phone. ASHAs will be making home visits and will use mobile phones as job aid. Detailed description of how ASHAs will use mobile phones can be found in section 10.1.1. ASHAs will assist ANMs during monthly VHND and enter information about blood pressure, haemoglobin, per abdominal examination, weight and urine examination as recorded by the ANM. ASHAs will come to PHC for monthly review meetings. She will report any technical problem to mHealth facilitator.

**Figure 5 Organogram**

**Investigator from govt**

Medical officers for Block 1

Medical officers for Block 2

Medical officers for Block 3

ANM for PHC 1

ANM for PHC 2

ANM for PHC 3

All ASHA for that PHC

Medical officers for Block 4

ASHA facilitators for that PHC

The mHealth facilitators will send SMSs to each ANM and medical officer every morning based on complicated cases identified by ASHAs over last 24 hours in addition to scheduled follow up of complicated cases identified over last few days. The ANM will make home visits or contact ASHA for cases with severe complications, confirm diagnosis, assess the status of the patient and try to convince household to refer the case a health facility. In case the family is unable to go to health facility then the ANM will provide treatment on the spot. Such treatment would include providing counseling, giving drugs, including approved antibiotics. The ANM will ensure that the ASHA is informed about the treatment plan and has clear understanding about what she needs to do over next few days, especially to ensure compliance with treatment. Along with attending to complicated cases, ANM will monitor activities of ASHAs.

The mHealth Facilitator will be first contact for ASHAs and other users to report any problem with mobile phone/application and other related issues arising from use of mHealth solutions. The mHF will NOT be involved in patient care or monitoring tasks and will not get in existing working arrangement within the govt. This person will facilitate use of mHealth solutions in existing govt system only.

Some of the specific tasks that mHF will undertake are:

- First contact person in case of any technology/mobile phone/ImTeCHO application related problems encountered by any users (ASHA, PHC staff). In such cases, ImTeCHO facilitator will troubleshoot the problem. If indicated, facilitator will escalate the problem to appropriate authority.
- Manage migration/duplicate cases/append case/death reporting through his/her login on web interface
- Attend PHC meetings for a brief time to troubleshoot any problems with mobile phones, motivation, including distribution of monthly ImTeCHO newsletter and update users about any changes in the application.
- Monitoring of adherence to the ImTeCHO intervention through use of innovative process indicators
- Remote supervision of ASHAs through phone in form of once a month performance review phone call by using information from ImTeCHO wed interface. Additionally, daily tracking of pre defined performance parameters available through web interface and follow up actions, if required. This includes contacting ASHAs for long overdue tasks, pending entry for mamta divas services and pending login for long stretch of time.
- Occasional field visit (support visit) to those ASHAs whose performance parameters within ImTeCHO is consistently poor and to provide ongoing training/supervision/motivation.
- SMS ANMs to inform about high risk cases
- Attend two-monthly ImTeCHO facilitators’ review meeting with program coordinator. There will be one program coordinator for ten facilitators.
- Facilitate calculation and disbursement of ImTeCHO related incentives
- Will NOT get involved in patient care, supply replenishment, grievance redressal etc.
- Overall, facilitator will spend 80% of time in office and 20% in field.

Medical officers will be responsible for tracking of high risk cases on daily basis in addition to presiding over routine monthly ASHAs meeting . The medical officer will assess performance of ANMs and ASHAs during these meetings in addition to supporting them in form of addressing any supply issues and payment of incentives. Complicated cases will be discussed during these meetings.

PHC support staff will use web interface to manage incentives and supplies. The staff will review and edit monthly ASHA activity report which will be used by ImTeCHO to automatically calculate incentives. The staff will enter information in web interface about payments made to ASHAs. Similarly, information about status of supplied and any “stocked out” items will be available on web.

SEWA Rural’s administrative staff and mHealth facilitators will manage migration, users, duplicate cases, amendment of any existing cases along with transcribe any information relayed through audio by ASHAs.

## 13.2 Timeline

Please see Section 10.3 and Table 10 for detailed time line.

## 13.3 Output of the project and deliverables

Some of the deliverables of this study are listed below:

- Complete software product- ImTeCHO including mobile phone application and web interface. ImTeCHO will be available for wider use without any development, and cost and source code would be made available on request for social good.
- Training tools, including training videos for ASHAs, medical officer and medical officers.
- Implementation plan for introducing ImTeCHO for MNCH care in larger context
- A comprehensive report
- Demonstration site

# 14. Budget

Please see Appendix 4 for budget and Appendix 3 budget justification. Total budget for this study is US $ 762, 951. The Indian Council of Medical Research has sanctioned US $ 407, 567. The proposal for obtaining funds for the remaining amount is being considered by the Department of Maternal, Newborn, Child and Adolescent Health of the World Health Organization and the MacArthur Foundation.

# 15. Data management and statistical analysis

## 15.1 Defining data needs and designing data collection tool

Data needs have been ascertained based on indicators to be measured. Dummy tables are prepared for final protocol. Data collection tool is designed based on survey tool for DLHFS-4 draft tool. Data collection team will be trained to administer tool to ensure standardization and to communicate data collection rules. The tool is already pretested and modified accordingly. Use of electronic data collection tool such as smart-phones is being considered to collect survey data.

## 15.2 Data flow process

Please see Figure 5 for data flow process.

**Figure 5 Data flow process for household surveys**

Data collector visits household

Data entry in smart phone using electronic household survey tool

Sync data from smart phone to interim database at the end of the interview

Data administrator reviews all data within 2 days of data collection date

Clarify inaccuracies with data collector within one week of data collection date

Data cleaning to be done by investigator and data administrator

“Release” data in main data center

Prepare final file for analysis

Data collection team will identify respondents for household survey using information from ongoing pregnancy registration and mortality surveillance as described in section 12. The data-collection team will enter data into smart-phones which will be loaded with data-collection tool with in-built checks for missing data and logical inconsistency. Data will be uploaded to a central server using GPRS where it will store in mysql database. A data-administrator will check for any inaccuracies and it will be clarified during review-meeting with data-collectors.

5% of households will be randomly selected and re-interviewed by an independent quality-assurance team using a truncated questionnaire. Data will be re-validated in case of more than 15% differences in key metrics are found. Data cleaning will be done as part of the ongoing process of identification of inaccuracies in the data, and subsequent rectification based on field inputs by the data-administrator, as per established protocols for editing and cleaning the data. Only investigators will have access to raw and personally identifiable data which will be protected by password. Any edits in the raw data-base will be allowed only after completing a query form. Any corrected data will be red-flagged. A separate form will be filled to state nature (who, when, why) for data corrections. Final data-set will be created once all surveys and surveillance activities are completed.

A data-center will be established at head-quarters. Protocol will be established ensuring security of data center. Data will be backed up on regular basis.

## 15.3 Data Safety and Monitoring Board

Data safety and monitoring board will be created made of independent scientists, ethicists, community member, and statistician and will meet on regular basis. DSMB will meet before initiation of study to review protocols.

## 15.4 Analysis plan

Data cleaning will be done using various relational checks and it will locked before analysis. Data analysis will be done as per the statistical analysis plan. Primary analysis will be intention to treat and secondary analysis would be per protocol. Data will be analysed taking tin to account cluster randomization. Analysis would be done at various levels i.e. at ASHA level, PHC level, subcenter level and at the beneficiary level. In the analysis at ASHA/subcenter/PHC level, cluster would be the PHC while for analysi at beneficiary level; outcomes would be considered correlated within the PHC in block and among the beneficiary under a fiven ASHA. Before comparing the primary and secondary outcomes at the ASHA or at the beneficiary level, both cluster level and the unit of analysis level characteristics would be compared. In case of any imbalance in any of the characteristics at cluster or at the beneficiary level, both cluster level and the unit of analysis level characteristics would be compared. In case of any imbalance in any of the characteristics at cluster or at the beneficiary level, adjustments will be done using Generalized Estimating Equation approach. Categorical variables will be summarized using frequency (5) and quantitative variables will be summarized at mean/median and SD/IQR, as appropriate. For each of primary and secondary outcomes, effect size (95% confidence interval, after adjustment if required, would be computed. STATA 13.0 will be used for data analysis. Results will be presented as per statistical analysis plan.

s

# 16. Quality assurance

## 16.1 For home surveys

Data collector will enter the data on a smart-mobile and a paper-based data collection tool. Regular checks with be done by quality assurance team to find any discrepancy between digital and paper records; such discrepancy will be clarified with data collector within 10 days Process indicators (forms per day, time taken to complete one form etc) will be put in place to track data collection. As described above, any inaccuracies will be dealt within one week of data collection; data collector might need to return to the source if inaccuracy can not be clarified off-site. A separate quality assurance team will re-interview 5% of households to check quality of data. Smart-phones will be GPS-enabled.

Protocols for data cleaning will be prepared. Protocols will be established for editing any data. Only investigators will have access to raw and personally identifiable data which will be protected by password. Any edits in raw data-base will be allowed only after completing a query form. Any corrected data will be red-flagged. A separate form will be filled to state nature (who, when, why) for data corrections. Procedures will be in place to transfer data files.

## 16.2 Service to be provided by ASHAs, ANMs and medical officer

Process indicators indicated in Appendix 1 will be used to ensure coverage and quality of intervention to be implemented. Investigators and medical officer will review process indicators three month during review meeting. Process indicators will be available at on-going basis on web interface using mHealth solutions. A sample process indicator report is available in Appendix 4.

## 16.3 Mobile application and server

The mHealth facilitator will be first contact for all mobile and web end issues that users might face. mHF will keep a log of all technical issues reported to him/her. Protocols will be prepared for escalation of technical issue identified by the users. Similarly, problem log will be created for the web interface and server. Technical issues will discussed during regular review meetings with Argusoft India Ltd.

## 16.4 General study implementation

This will be reviewed during once a month meeting at head-quarter to be attended by on-site investigators and mHealth facilitators. Progress of the implementation will be assessed against proposed timeline. These meetings will be a platform to address issues identified during implementation of the project.

Steering committee meeting will be organized every twelve months during which issues related to coordination among various stakeholders will be discussed. Schedule of all monitoring and logistic meeting is indicated Table 12. It should be noted that regular meetings similar to ASHA and ANM review meetings are expected to take place now (in absence of the study) as per the protocol from the Ministry of Health and Family WelfareError: Reference source not found.

**Table 12** Schedule of meetings for quality assurance

| Name of meeting | Goal of meeting | Location | Frequency | Attendees |
| --- | --- | --- | --- | --- |
| PHC meetings (control and intervention area) | Review and support ASHA activities | PHC | Monthly | ASHAs, ANMs, medical officer and PHC staff, mHF |
| Implementation review meeting | Review process indicators and implementation related issues | Chief District Health Officer Office | Quarterly | Investigators, medical officers |
| Data collection review meeting | Review data collection | Head quarter* | Monthly during data collection period | On-site investigators, data collectors |
| Steering committee meeting | Review implementation of intervention | Head quarter | Once every twelve months | Steering committee member, investigators |
| Data Safety and Monitoring Board meeting | Review issues related to data safety and monitor data | Head quarter | Once a year | DSMB member, investigators |

*Head quarter mean SEWA Rural main campus at Jhagadia

# 17. Ethical and safety considerations

## 17.1 Areas of risk and potential solution

1. Data collection team might come across other morbidities among respondents. Patients will be referred to hospital irrespective of intervention or control cluster. Referred individuals will be given choice to go to SEWA Rural hospital which is providing outpatient and inpatient care to local community for more than 3 decades. 60% of outpatient and 80% inpatient care at SEWA Rural is provided free, if patient is non-affording.
2. An information sheet will be prepared in vernacular language having details regarding goals of the study along with potential risks and benefits to the participants and society. The data-collectors will give and explain information sheet to all the participants and address any questions. A written consent will be obtained from all consenting participants.
3. All personally-identifiable information will be removed and unique-number will be assigned to participants. Only principal investigators will have access to data linking personally-identifiable information with unique-number. The database will be protected by a password.
4. Privacy during data collection from ASHA and confidentiality of collected data will be strictly enforced to protect them from any harm from their supervisors (medical-officers etc) for revealing uncomfortable information regarding supervision and support.
5. Confidentiality of medical information to be recorded and transmitted using mobile-phone technology will be strictly enforced. All sensitive data will be codified and encrypted. Firewall will be used. The web-interface will be protected by password. The database server will be installed at a Tier 1 high security data-center.
6. Individual medical information will be collected using mobile phone and will be stored on server. This raises a concern regarding confidentiality of medical information. The proposed application shall contain medical information of individuals and hence demands high levels of data security. In view of this, the application shall accept, transmit, process and store data using prudent security and data encryption practices as described below.
   - Any data that is transmitted using GPRS from the Mobile Phone to the server and vice versa shall be encrypted. In addition, the data shall also be codified before encryption to provide an additional layer of obfuscation.
   - A high level of security measures shall also be applied at the database level. The database server shall be protected by a firewall with strict access permissions such that there is no direct access to the database. Only the designated web application can connect to the database using a secured connection.
   - A web application firewall shall be used to protecting the site against cross-site scripting vulnerabilities and web site vandalism. It shall also protect the data from SQL injection attacks as well
   - All sensitive data elements shall be encrypted and stored in the database. Also a level of indirection in the form of encrypted user identities shall also be introduced so that a person's personal information and their medical information can never be co-related other than through the web application.
   - Web interface will be protected by password; hence, only authorized personnel will be able to access information.
   - The database server shall also be configured behind the firewall and shall have network access only to the webserver. The application shall be able to access the database server to prevent any other application or system to try and break into this data.
   - The database server as well as the web application shall be installed at a Tier 1 high security data centre to prevent unauthorized physical access to the webservers and the database servers.

## 17.2 Areas of benefit

Whole study patient population will benefit by this study. Pregnant women, newborns, and children in intervention area will receive all components of interventions which are proven to improve health outcomes. In control area, ASHAs will be re-trained to provide traditional care without use of mHealth solutions. ImTeCHO will provide support to all health workers at PHC level to make their existing job easier, effective and enjoyable. ASHAs stand higher chance of receiving timely and accurate incentives using mHealth solutions. ASHAs will have access to ANM and referral hospital in case a complicated case is identified. PHC staff will be benefitted as calculation of incentives and supply management will be less effort intensive. ANMs will have their monthly reports prepared automatically and on timely basis; thus, saving large number of man-hours.

## 17.3 Ethical review

Informed consent will be obtained from all respondents prior to household survey. Also, consent will be requested from all study participants (ASHAs, ANMs, medical officer, medical officers). Study is being registered at the Clinical Trial Registry of India (ctri.nic.in) and acknowledgement number for the submitted application is REF/2015/05/008920. The Multi-institutional Ethics Committee, Mumbai (of which SEWA Rural is a member) has reviewed and approved the study. Appendix 5 has institutional ethical clearance from Multi-institutional Ethics Committee. Appendix 6 contains patient information and consent form.

# 18. Dissemination of results and policy implications of study:

## 18.1 Uniqueness of ImTeCHO compared to other models

There are hundreds of mHealth pilots projects being implemented worldwide; However, ImTeCHO is unique in following ways. (1) Leverages upon SEWA Rural’s three decades of grass roots experience and insight generated from implementing various community health programs. (2) Active involvement of the government’s health department in all areas including planning, developing, implementing and evaluating ImTeCHO (3) Led and supported by in-country team in India regarding all aspects of ImTeCHO including its development, implementation and evaluation (4) A comprehensive mHealth solution to support all aspects of ASHA program management and addresses critical implementation challenges such as regular payment of incentives, replenishment of supplies, and performance management (5) Use of state of art mobile phone technology encompassing multimedia, decision tree, patient support, emergency management and more (6) Potentially interoperable with online mother and child tracking system (MCTS) and can compliment MCTS in future (7) Strong focus on robust and high-quality research and evaluation (8) Complete package of implementation plan/roll out plan is field tested and ready for systematic and effective scale up. (9) ImTeCHO is being implemented by government’s health workers/ASHAs instead of SEWA Rural’s own health workers.

## 18.2 Implications on policy

Insufficient evidence regarding effectiveness to improve outcomes, cost-effectiveness and feasibility has been one of the most important barriers for scaling mHealth solutionsError: Reference source not found, [[49]](#footnote-50). This study will help answer some critical questions about effectiveness and feasibility of implementing mHealth solution in area of MNCH. If ImTeCHO is found to be effective and feasible then investment of resources at scale for replicating this model can be justified. More research after appropriate modification to proposed intervention will be advised if intervention is found not to be effective and/or not feasible in real-life situation. Some of the specific areas of impact are listed below.

1. Web interface can be easily integrated into existing MCTS system which can help to further enhance inherent usefulness of MCTS.
2. More importantly, this study will guide “how” mHealth solutions can be implemented in existing public health system to strengthen community processes for areas other than MNCH such as Tuberculosis, malaria case management, chronic diseases etc. Hence, ImTeCHO could be useful for promoting universal health coverage through ASHAs.
3. This study and its deliverables will provide exact road-map to be followed for implementing mHealth solutions for community based MNCH. Deliverables of this study such as mobile phone applications, implementation plan, training modules, and videos can be adapted in other states immediately for wider use as these will be based on existing policies and health system in place.
4. Using mobile phone technology successfully to collect data regarding maternal, newborn and child health process indicators and outcomes along with reporting of births and deaths would be a very useful for surveillance.

## 18.3 Plan for dissemination

The goal of dissemination would be to inform all potential users about effectiveness of using ImTeCHO and advocate use of ImTeCHO among other underprivileged communities. Potential audience for dissemination will be government officials, policy makers, academics, researchers, local community and other voluntary organizations involved with community based MNCH services. To reach these varies audience, a multipronged dissemination and advocacy strategy will be required.

We would publish the findings of the study in peer reviewed journals. We will invite above audience to a dissemination seminar which will be organized at the end of project. We will submit abstracts in variety of conferences. Gujarat has couple of vibrant conglomerates of voluntary organizations involved in health sector; we will disseminate information about ImTeCHO whenever opportunity presents during meetings of this groups. We would use press and television media to share findings with local community.

# 19. Problems anticipated:

We need to assess risk of some un-planned events during the course of study which can have impact on it. Some of the possibilities are:

- New schemes from government such as starting hotline, voice messaging system etc.
- New service provider/NGO starts serving either control or intervention area which can impact outcomes.
- New regulations regarding use of mobiles from the government.
- Major staffing issues can impact outcomes.

If there is major reorganization in PHCs by the government then drop out of selected PHCs might be inevitable. Appropriate decision will be taken on case by case basis and record will be maintained to document actions taken.

SEWA Rural will closely work with the government and other stakeholders to mitigate or minimize above and other unanticipated risks while preserving ethical and moral values.

# 20. Strengths of research team and collaborations

## 20.1 About investigators

CVs of Drs. Pankaj Shah, Shrey Desai, Dhiren Modi, Kapil Dave, Shobha Shah, and Gayatri Desai are attached with the proposal.

The principal investigator currently is not involved in any other research project.

**Dr. Nishith Dholakia**

Dr. Dholakia is a pediatrician and serving in government for three decades in various capacities. Now, Dr. Dholakia is deputy-director at Commissioner of Healthâ€™s office and is leading maternal and child health division for whole state. Dr. Dholakia oversees implementation of MCH programs and drafts policies for further consideration. For this study, Dr. Dholakia will lead implementation of intervention within existing ASHA-program. He will provide technical input for development of intervention. He will be one of the main people responsible for scaling-up the intervention eventually.

**Ravi Gopalan**

President & CEO of ArguSoft India Ltd, Ravi has been a serial entrepreneur and has over 21 years of experience encompassing Systems Engineering, Technology, Finance and Operations, wearing different hats as CEO, CTO, COO. Having founded ArguSoft, he has had hands on experience in building the team, infrastructure and managing the operations of the company while playing the key role in the Strategic Vision and Business Development.

He has been instrumental in creating numerous ICT solutions for various large and medium businesses over the past several years. In recent years, his focus has been to create technology solutions as platforms and deliver applications on small devices such as mobile phones and tablets for mobile workforces specially in the life sciences and public health care domains. Some of the recent projects in this space include an ICT solution for the PPTCT program to collect, track, and monitor and provide health services for HIV + individuals. Another recent solution was for the collection of field data for the analysis and representation of human development index. The field level data collection was done using low cost mobile phones as the point of contact device.

Ravi has graduate degrees in MS (Management Science) and MS (Computer Science) from Florida Institute of Technology/USA.

## 20.2 Roles and responsibilities of all investigators

Roles and responsibilities of all investigators are included in justification of budget in Appendix 3.

## 20.3 About organizations involved in the study

### 20.3.1 SEWA Rural

Following section is regarding strength of the 3 organizations involved in this study.

SEWA Rural is a voluntary development organization involved in health & development activities in rural, tribal area of Gujarat, India since 1980. Aiming for overall development of the rural, poor and tribal population Bharuch Dist., the organisation has encompassed various fields including hospital, community based outreach health care, comprehensive eye care, health training centre, vocational training institute for rural youth and women development center for promoting women empowerment. Some more information about SEWA Rural will be available in our website [www.sewarural.org](http://www.sewarural.org/). It is important to note that SEWA Rural implemented Comprehensive Eye Care (CEC) project in 8 surrounding blocks from 1997 to 2012 including 3 blocks to be included in this study. SEWA Rural has good relationship with local communities of these blocks because of eye care project which will be vital for implementing this study. Also, a state of art training centre will be helpful to provide training to participants of this study.

In the area of community health, SEWA Rural implemented two major projects: The government handed over responsibility of one PHC to SEWA Rural in 1985 to 1999 under public-private partnership model for the first time in history. SEWA Rural was able to achieve all target for “Health for all” well before completing the project. From April’2003 to March’2011, SEWA Rural implemented a family centered safe motherhood and newborn survival project covering 171,000 population of Jhagadia block. This project included comprehensive community level interventions which were complemented by First Referral Unit (FRU) providing Comprehensive Emergency Obstetric and Newborn Care (CEmONC) services. Supported by the John D. & Catherine T. MacArthur Foundation, SEWA Rural was able to reduce Maternal Mortality Ratio by 75% and NMR by 38%Error: Reference source not found. It received the prestigious “MacArthur Award” in 2007 for being a “*creative and effective institution*” for this project.

Evaluation reports for above projects are available in SEWA Rural’s website.

Over last one year, SEWA Rural has been in contact with few IT companies which are involved in mHealth arena. SEWA Rural has closely studied critical aspects of each of these companies including cost, capability to develop software, experience and most importantly passion for the social cause. Though all IT companies were exceptional, Argusoft India Ltd was chosen to be IT partner for this study after all considerations. SEWA Rural and Argusoft have already taken initial steps for working toward developing the intervention.

### 20.3.2 Argusoft India Ltd.

Argusoft is a growing software development organization with a solid value system and clear business practices. Argusoft believes that the purpose of technology is human convenience. Its mission is to enable organizations and individuals to benefit from cutting edge software and web-based technologies as an extension of their team. Argusoft is fortunate to have an advisory board consisting of industry luminaries both in India and the USA. Argusoft has prepared two mobile phone applications in the area of mHealth. Argusoft’s other e-health initiative is in tele-ophthalmology for Tripura Vision Center.

More information about Argusoft can be found at their website http://www.argusoft.com

### 20.3.3 Department of Health and Family Welfare, Government of Gujarat

The Gujarat state health department will be leading implementation of the intervention within primary health care system. The government health functionaries from PHC to block to district level will be involved in implementation with nodal officer being Dr.Nishith Dholakia at state commissionerate office at Gandhinagar.

20.3.4 ICMR and WHO

The ICMR will provide technical and financial support. The experts from WHO and ICMR will provide technical assistance.

# 21. Links to other projects

A few nested studies are being planned during the course of the trial. Some of the potential studies might be as following.

- Examine effect of ImTeCHO intervention in the form of job-aid to ASHAs, ANMs, medical officers and PHC staff to reduce NMR and IMR in tribal areas of Gujarat.
- Examine cost-effectiveness of ImTeCHO intervention in the form of job-aid to ASHAs, ANMs and medical officers to increase coverage of selected MNCH interventions and care to be provided for complicated maternal, newborn and child cases in tribal areas of Gujarat.
- Improving coverage of Kangaroo Mother Care (KMC) using an innovative mHealth intervention
- Examine effectiveness of mHealth intervention on skills and knowledge of ASHAs
- Examine completeness of pregnancy and delivery records among ImTeCHO
- Examine effectiveness of ImTeCHO towards reducing prevalence of underweight
- Comparison of coverage of proven MNCH services between areas using ImTeCHO within a government health system and a voluntary organization

# 21. Feasibility of completing the study in time

Please see Section 10.3 for detailed timeline.

Total study time period will be 36 months. Considering ability and experience of teams involved, it will be feasible to complete the study in time. Two of the most crucial stages for successfully completing study are development and piloting of intervention in addition to maturation phase. It should be noted that SEWA Rural and Argusoft has completed development of software and piloting is almost complete. A period of 4 months has been proposed for intervention maturation based on SEWA Rural’s previous experience while working on mHealth solutions. Therefore, early preparations and enough duration for intervention maturation increase the chances of completing study on time.

# 22. Abbreviations

ANC- Antenatal Care

ANM- Auxiliary Nurse Midwife

ASHA- Accredited Social Health Activist

AWW- Anganwadi Worker

AYUSH (Ayurveda, Yoga & Naturopathy, Unani, Siddha and Homoeopathy)

BF- Exclusive breast feeding for six months

BCG- Bacille Calmette Guerin

CCI- Composite Coverage Index

CEmONC- Comprehensive Emergency Obstetric and New Born Care

CF- Initiation of complimentary solid, semi-solid or soft foods

DLHFS- District Level House Hold and Facility Survey

DPT- Diphtheria, Pertussis and Tetanus

ERT- Emergency Response Team

FPS- Family Planning needs satisfied

FRU- First Referral Unit

HQ- Headquarters

ICMR- Indian Council of Medical Research

IFA- Iron-Folic Acid

IMCI- Integrated Management of Childhood Illnesses

IMNCI- Integrated Management of Neonatal and Childhood Illnesses

IMR- Infant Mortality Rate

MACCI - Modified ASHA-centric composite coverage index

MCH- Maternal Child Health

MCTS- Maternal and Child Tracking System

MMR- Maternal Mortality Ratio

MNCH- Maternal , New Born and Child Health

MSL- Measles vaccination

NGO- Non Government organization

NIC- National Informatics Center

NICU-Neonatal Intensive Care

NMR- Neonatal Mortality Rate

NRHM-National Rural Health Mission

PHC- Primary Health Center

PNC-Postnatal Care

SBA- Skilled birth attendance

SC- Schedule Caste

SEWA-Rural- Society for Education, Welfare and Action- Rural

ST- Schedule Tribe

TBA- Trained Birth Attendant

TT Injection-Tetanus Toxoid Injection

URL- Uniform Resource Locator

VHND- Village Health and Nutrition Day

WHO- World Health Organization

# 23 Appendices

Appendix 1: Indicators for primary and secondary objectives

Appendix 2: Data collection tool for survey at baseline and endline

Appendix 3: Justification of budget

Appendix 4: ASHA performance indicators

Appendix 5: Institutional Review certificate

Appendix 6: Patient information and consent form

Appendix 7: Comments by the ICMR review committee and point-by-point response from investigators

1. Jennifer Bryce, Sham el Arifeen, George Pariya, Claudio F Lanata et al. Reducing child mortality: can public health deliver? *Lancet* 2003; 362: 159-64. [↑](#footnote-ref-2)
2. Kumar, V.P., Sachdev, H., Mavalankar, D., Ramachandran, P., Sankar, M., Bhandari, N., et al. Reproductive health, and child health and nutrition in India: meeting the challenge. *Lancet* 2011;377 (9762): 332-349. [↑](#footnote-ref-3)
3. SEWA Rural. Report on Family Centered Safe Motherhood and Newborn Care Project. Jhagadia, 2011 [↑](#footnote-ref-4)
4. Columbia University. Improving the performance of Accredited Social Health Activists in India: Working papers series. Mumbai. 2011 [↑](#footnote-ref-5)
5. Guidelines on ASHA. http://www.mohfw.nic.in/NRHM/Task_grp/Guidelines%20on%20ASHA.pdf. Accessed on 7 June, 2012. [↑](#footnote-ref-6)
6. National Rural Health Mission. ASHA module 6: Skills that saves lives. [↑](#footnote-ref-7)
7. NRHM. Home based newborn care, operational guidelines. New Delhi, 2011. [↑](#footnote-ref-8)
8. Department of Health and Family Welfare, Government of Gujarat. National Rural Health Mission. Online mother and child tracking system. Operational manual. [↑](#footnote-ref-9)
9. http://www.thelancetstudent.com/legacy/category/articles/ (accessed June 5, 2012) [↑](#footnote-ref-10)
10. <http://dst.gujarat.gov.in/awards-2012.htm> (accessed June 5, 2012) [↑](#footnote-ref-11)
11. National Health Systems Resource Centre (NHSRC) and National Rural Health Mission (NRHM). ASHA: Which way forward...? New Delhi, 2011. [↑](#footnote-ref-12)
12. District Level Health and Facility Survey-3 (DLHFS-3), Gujarat [↑](#footnote-ref-13)
13. articles.timesofindia.indiatimes.com/2011-10-22/varanasi/303 - 40k. Accessed on 17/5/12. [↑](#footnote-ref-14)
14. Results of mid-term evaluation done by SEWA Rural among 70 villages of Jhagadia block in June, 2014. The data shown above is for the comparison area. [↑](#footnote-ref-15)
15. Bang AT, Bang RA, Morankar VP, et al. Pneumonia in neonates: can it be managed in the community? *Arch Dis Child* 1993; **68:** 550–56. [↑](#footnote-ref-16)
16. Sutrisna B, Reingold A, Kresno S, et al. Care-seeking for fatal illness in young children in Indramayu, West Java, Indonesia. *Lancet* 1993; **342:** 887–89. [↑](#footnote-ref-17)
17. Bhandari N, Bahl R, Bhatnagar V, Bahn MK. Treating sick young infants in urban slum setting. *Lancet* 1996; **347:** 1174–75 [↑](#footnote-ref-18)
18. Dongre AR, Deshmukh PR, Garg BS. Perceptions and health care seeking about newborn danger signs among mothers in rural

    Wardha. Indian J Pediatr 2008;75:325-329.). [↑](#footnote-ref-19)
19. Kumar R, Jaiswal V, Tripathi S, Kumar A, Idris MZ. Inequity in health care delivery in India: the problem of rural medical practitioners. Health Care Anal 2007;15:223-233. [↑](#footnote-ref-20)
20. Save the children, 2008. http://www.savethechildren.org.uk/news/2008/02/the-life-or-death-lottery-inequality-and-injusticein-the-fight-to-save-childrens-lives (accessed November 29, 2011). [↑](#footnote-ref-21)
21. NHSRC. An update on ASHA programme. 2013. [↑](#footnote-ref-22)
22. [http://www.mohfw.nic.in/WriteReadData/l892s/9612225483Minutes%20of%20State%20Health%20Secretary%20&%20MD%20Meeting%20held%20on%207-8th%20July%202011.pdf](http://www.mohfw.nic.in/WriteReadData/l892s/9612225483Minutes of State Health Secretary & MD Meeting held on 7-8th July 2011.pdf) (accessed June 5, 2012) [↑](#footnote-ref-23)
23. <http://informatics.nic.in/Lead_Story1.php> (accessed June 5, 2012) [↑](#footnote-ref-24)
24. The solution exchange for maternal and child health community. Payment of incentives to ASHAs- bottlenecks and good practices. Available from *ftp://ftp.solutionexchange.net.in/public/mch/cr/cr-se-mch-06071201.pdf (*Accesses on 12 September, 2012). [↑](#footnote-ref-25)
25. Dhiren Modi, Ravi Gopalan, Shobha Shah, Sethuraman Venkatraman,Gayatri Desai, Shrey Desai and Pankaj Shah.[Development and formative evaluation of an innovative mHealth intervention for improving coverage of community-based maternal](http://sewarural.org/sewa/wp-content/uploads/2015/02/ImTeCHOdevelopment.pdf), newborn and child health services in rural areas of India. Glob Health Action 2015, 8: 26769. [↑](#footnote-ref-26)
26. Gawande, Atul. Checklist Menifesto. New Delhi: Penguin India, 2011. [↑](#footnote-ref-27)
27. Svoronos, T., Mjungu, D., Dhadialla, P., et al. (2010) CommCare: Automated Quality Improvement To Strengthen Community- Based Health. Available at: http://d-tree.org/wp-content/ uploads/2010/05/Svoronos-Medinfo-CommCare safepregnancy1. pdf [↑](#footnote-ref-28)
28. Holmes, D. (2010) Rwanda: an injection of hope. *Lancet,* 376, 945–946 [↑](#footnote-ref-29)
29. Grameen Foundation. Mobile phone technology for community health (MOTECH) in Ghana. 2011. Available on <http://www.grameenfoundation.org/sites/default/files/MOTECH-Early-Lessons-Learned-March-2011-FINAL.pdf>. Accessed on 7 June, 2012. [↑](#footnote-ref-30)
30. Derek Treatmean, Neal Lesh. Strenghthening Community Health Systems with Localized Multimedia. Proceedings of M4D2012, New Delhi, Editors Vikas Kumar and Jakob Svensson. Karlstad University Studies. p 7-22 [↑](#footnote-ref-31)
31. Brian DeRenzi, Neal Lesh, Tapan Parikh, Clayton Sims, Marc Mitchell, Werner Maokola, Mwajuma Chemba, Yuna Hamisi, David Schellenberg and Gaetano Borriello, e-IMCI: Improving Pediatric Health Care in Low-Income Countries, Proceedings of CHI '08. [↑](#footnote-ref-32)
32. Richard T Lester, Paul Ritvo, Edward J Mills *et al.* Effects of a mobile phone short message service on antiretroviral treatment adherence in Kenya (WelTel Kenya1): a randomised trial *Lancet* 2010; 376: 1838–45. [↑](#footnote-ref-33)
33. Dejan Zurovac, Raymond K Sudoi, Willis S Akhwale, *et al*. The effect of mobile phone text-message reminders on Kenyan health workers’ adherence to malaria treatment guidelines: a cluster randomised trial *Lancet* 2011; 378: 795–803. [↑](#footnote-ref-34)
34. Noordam, C., A., Kuepper, B., M., Stekelenburg, J., Milen, A. (2011) Improvement of maternal health services through the use of mobile phones. [*Trop Med Int Health*](http://www.ncbi.nlm.nih.gov/pubmed?term=Improvement of maternal health services through the use of mobile phones)*,* 16(5), 622-6. [↑](#footnote-ref-35)
35. WHO and UNICEF. Home visits for newborn child: a strategy to improve survival: WHO and UNICEF joint statement. 2009. [↑](#footnote-ref-36)
36. Government of Gujarat. Government resolution regarding roles of ASHA, Anganwadi worker and ANM. 24/9/2012. [↑](#footnote-ref-37)
37. # [**Victora CG**](http://www.ncbi.nlm.nih.gov/pubmed?term=Victora CG%5BAuthor%5D&cauthor=true&cauthor_uid=22999433), [**Barros AJ**](http://www.ncbi.nlm.nih.gov/pubmed?term=Barros AJ%5BAuthor%5D&cauthor=true&cauthor_uid=22999433), [**Axelson H**](http://www.ncbi.nlm.nih.gov/pubmed?term=Axelson H%5BAuthor%5D&cauthor=true&cauthor_uid=22999433), [**Bhutta ZA**](http://www.ncbi.nlm.nih.gov/pubmed?term=Bhutta ZA%5BAuthor%5D&cauthor=true&cauthor_uid=22999433), [**Chopra M**](http://www.ncbi.nlm.nih.gov/pubmed?term=Chopra M%5BAuthor%5D&cauthor=true&cauthor_uid=22999433), [**França GV**](http://www.ncbi.nlm.nih.gov/pubmed?term=França GV%5BAuthor%5D&cauthor=true&cauthor_uid=22999433), [**Kerber K**](http://www.ncbi.nlm.nih.gov/pubmed?term=Kerber K%5BAuthor%5D&cauthor=true&cauthor_uid=22999433), [**Kirkwood BR**](http://www.ncbi.nlm.nih.gov/pubmed?term=Kirkwood BR%5BAuthor%5D&cauthor=true&cauthor_uid=22999433), [**Newby H**](http://www.ncbi.nlm.nih.gov/pubmed?term=Newby H%5BAuthor%5D&cauthor=true&cauthor_uid=22999433), [**Ronsmans C**](http://www.ncbi.nlm.nih.gov/pubmed?term=Ronsmans C%5BAuthor%5D&cauthor=true&cauthor_uid=22999433), [**Boerma JT**](http://www.ncbi.nlm.nih.gov/pubmed?term=Boerma JT%5BAuthor%5D&cauthor=true&cauthor_uid=22999433). How **changes** in **coverage** **affect** **equity** in **maternal** and **child health** **interventions** in **35** **Countdown** to **2015** **countries**: an **analysis** of **national** **surveys**. [**Lancet.**](http://www.ncbi.nlm.nih.gov/pubmed/?term=How+changes+in+coverage+aff+ect+equity+in+maternal+and+child+health+interventions+in+35+Countdown+to+2015+countries%3A+an+analysis+of+national+surveys) 2012 Sep 29;380(9848):1149-56. doi: 10.1016/S0140-6736(12)61427-5.

    [↑](#footnote-ref-38)
38. Boerma JT, Bryce J, Kinfu Y, Axelson H, Victora CG. Mind the gap: equity and trends in coverage of maternal, newborn, and child health services in 54 Countdown countries. *Lancet* 2008; **371:** 1259–67. [↑](#footnote-ref-39)
39. Barros AJ, Ronsmans C, Axelson H, et al. Equity in maternal, newborn, and child health interventions in Countdown to 2015: a retrospective review of survey data from 54 ountries. *Lancet* 2012; **379:** 1225–33. [↑](#footnote-ref-40)
40. Countdown to 2012: *Building a Future for Women and Children: The 2012 Report*. 2012. [↑](#footnote-ref-41)
41. The Ministry of Health and Family Welfare. Indian Public Health Standards (IPHS) For Primary Health Centres. http://mohfw.nic.in/NRHM/Documents/IPHS_for_PHC.pdf. New Delhi. 2006. [↑](#footnote-ref-42)
42. Ministry of Panchayati Raj (September 8, 2009). ["A Note on the Backward Regions Grant Fund Programme"](http://www.nird.org.in/brgf/doc/brgf_BackgroundNote.pdf). National Institute of Rural Development. Retrieved September 27, 2011. [↑](#footnote-ref-43)
43. UNICEF. Coverage evaluation survey-2009. New Delhi, India. [↑](#footnote-ref-44)
44. District Level Health and Facility Survey-3 (DLHFS-3), Narmada [↑](#footnote-ref-45)
45. National Rural Health Mission. Handbook for ASHA Facilitators. New Delhi. [↑](#footnote-ref-46)
46. National Rural Health Mission (NRHM) and Ministry of Health and Family Welfare. A handbook for Auxiliary Nurse Midwife (ANM), Lady Health Visitor (LHV) and staff nurses. New Delhi. 2010. [↑](#footnote-ref-47)
47. Kumar V, Kumar A, Darmstadt G. Behaviour change for newborn survival in resource-poor community settings: Bridging the gap between evidence and impact. *Semin Perinatol 34:446-461.*  [↑](#footnote-ref-48)
48. International Institute for Population Sciences District Level Household and Facility Survey (DLHS-4). Bid Document [http://www.rchiips.org/pdf/DLHS4%20Bid%20for%20field%20agency%20at%201IIPS,Mumbai..pdf](http://www.rchiips.org/pdf/DLHS4 Bid for field agency at 1IIPS,Mumbai..pdf). Accessed on 7 June, 2012. [↑](#footnote-ref-49)
49. Center for Global Health and Economic Development Earth Institute, Columbia University Barriers and Gaps Affecting mHealth in Low and Middle Income Countries: Policy White Paper. New York. 2010. [↑](#footnote-ref-50)
