# Supplementary material for: mHealth intervention “ImTeCHO” to improve delivery of maternal, neonatal, and child care services—A cluster-randomized trial in tribal areas of Gujarat, India
Source: PLoS Med. 2019 Oct 24;16(10):e1002939. doi: 10.1371/journal.pmed.1002939 (PMC6812744; doi:10.1371/journal.pmed.1002939)
Supplement: S2 Text — (DOCX) [file pmed.1002939.s004.docx]

1. Proportion of neonates/mothers who were visited at home by ASHA at least twice within first week of delivery.

Three home visits within first week of birth by frontline health workers, including on the day of delivery, the third day and preferably the seventh day is known to reduce neonatal mortality. However, ASHAs’ home visit on the day of birth was no more relevant in most of the cases because majority of the deliveries in Gujarat now occur at facilities. Hence, the coverage of two home-visits within first week of delivery was chosen as a primary outcome of interest.

2. Modified ASHA-centric Composite coverage index (MACCI) [23]

To measure effectiveness of ImTeCHO throughout the continuum of care including pregnancy, postpartum and infancy period, a composite coverage index was developed and calculated. The development of new index was guided by another Composite coverage index (CCI) which had already been used as a measure of coverage of key RMNCH services. We modified the formula for calculating CCI to develop a modified ASHA-centric Composite Coverage Index (MACCI) by focusing on the (i) ASHA’s role towards provision of MNCH services in India (ii) ImTeCHO intervention’s scope and (iii) relevance for India.

MACCI was calculated using following formula.

Modified ASHA-centric composite coverage index (MACCI) = 0·25 × (0.33 × [Complete ASHA home visits during antenatal period +Full ANCS+FD] + [Complete HBNC] + 0·5 × [DPT3 + EBF] + 0·33 × [Care seeking for neonatal complications + ORT + ARI/febrile illness])

In which,

1. Maternal care services
   - Complete ASHA home visit during antenatal period = proportion of mothers who were visited at home by ASHAs at least three times during last pregnancy
   - Full Antenatal care service (ANCS) by ANM or doctor at community or facility= proportion of mothers with full antenatal examination (at least three antenatal examinations by a qualified health personnel, one Injection Tetanus Toxoid and consumption of 100 Iron, Folic Acid (IFA) tablets)
   - Health facility delivery (FD) = proportion of mothers who delivered in a health facility
2. Neonatal care services
   - Complete home based newborn care (HBNC) = proportion of neonates who received the recommended number (five) of postnatal visits and at recommended times within first month of delivery by ASHA
3. Young infant care services
   - Three doses of Diphtheria, Pertussis and Tetanus Toxoid (DPT3) by ANM or other qualified health provider at community or facility= proportion of infants (6-8 months) who received three doses of diphtheria, pertussis, and tetanus vaccine or three doses of pentavalent vaccine
   - Exclusive Breast Feeding (EBF) = proportion of infants who were exclusively breast fed during the previous day
4. Care seeking during complications
   - Care seeking for neonatal complications = proportion of neonates who had complications within first month of last delivery and sought care from ASHA
   - Oral rehydration treatment (ORT) = proportion of infants (6-8 months) who had diarrhoea within last two weeks and received ORS from ASHA
   - Acute respiratory illness (ARI)/fever = proportion of infants (6-8 months) with ARI/fever within last two weeks and sought care from ASHA

Each of the four main domains were given same weight.
